# Modular micro-PCR system for the onsite rapid diagnosis of COVID-19

**DOI:** 10.1038/s41378-022-00400-3

**Published:** 2022-07-19

**Authors:** Phuong Quoc Mai Nguyen, Ming Wang, Nelisha Ann Maria, Adelicia Yongling Li, Hsih Yin Tan, Gordon Minru Xiong, Meng-Kwang Marcus Tan, Ali Asgar S. Bhagat, Catherine W. M. Ong, Chwee Teck Lim

**Affiliations:** 1 Institute for Health Innovation and Technology (iHealthtech), National University of Singapore, MD6, 14 Medical Drive #14-01, Singapore, 117599 Singapore; 2 Department of Biomedical Engineering, National University of Singapore, 4 Engineering Drive 3, Block 4, #04-08, Singapore, 117583 Singapore; 3 Advanced MedTech, 2 Venture Drive, #23-18 Vision Exchange, Singapore, 608526 Singapore; 4 Infectious Diseases Translational Research Programme, Department of Medicine, Yong Loo Lin School of Medicine, National University of Singapore, NUHS Tower Block, 1E Kent Ridge Road Level 11, Singapore, 119228 Singapore

**Keywords:** Chemistry, Electrical and electronic engineering

## Abstract

Effective containment of the COVID-19 pandemic requires rapid and accurate detection of the pathogen. Polymerase chain reaction (PCR) remains the gold standard for COVID-19 confirmation. In this article, we report the performance of a cost-effective modular microfluidic reverse transcription (RT)-PCR and RT-loop mediated isothermal amplification (RT-LAMP) platform, Epidax®, for the point-of-care testing and confirmation of SARS-CoV-2. This platform is versatile and can be reconfigured either for screening using endpoint RT-PCR or RT-LAMP tests or for confirmatory tests using real-time RT-PCR. Epidax® is highly sensitive and detects as little as 1 RNA copy per µL for real-time and endpoint RT-PCR, while using only half of the reagents. We achieved comparable results with those of a commercial platform when detecting SARS-CoV-2 viruses from 81 clinical RNA extracts. Epidax® can also detect SARS-CoV-2 from 44 nasopharyngeal samples without RNA extraction by using a direct RT-PCR assay, which shortens the sample-to-answer time to an hour with minimal user steps. Furthermore, we validated the technology using an RT-LAMP assay on 54 clinical RNA extracts. Overall, our platform provides a sensitive, cost-effective, and accurate diagnostic solution for low-resource settings.

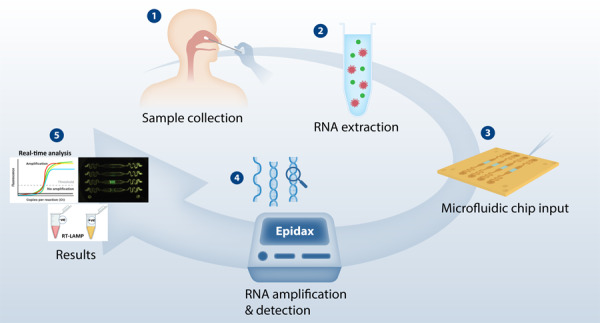

## Introduction

Since its emergence in December 2019, severe acute respiratory syndrome coronavirus 2 (SARS-CoV-2) has been spreading worldwide, causing deaths, illness, and disruption to lives and businesses.

Effective control of the COVID-19 pandemic requires rapid and accurate detection of the virus. The initial publication of the SARS-CoV-2 genome in January 2020 enabled companies and laboratories worldwide to develop various assays and techniques for COVID-19 diagnosis^1–3^. Real-time reverse transcription-polymerase chain reaction (RT–PCR) remains the gold standard for the identification of SARS-CoV-2. As a comprehensive testing program would likely require both widely accessible and rapid screening in combination with highly sensitive PCR confirmatory tests, there is a need for alternative methods to identify infected individuals that are sufficiently low cost, portable and effective to enable rapid diagnosis at the point of use. These point-of-care (POC) tests are an important tool to be deployed at borders for opening up international travel and economies.

Various commercial lab-based and POC tests have been deployed globally: Cepheid® Xpert® Xpress SARS-CoV-2^4^, Roche’s cobas® SARS-CoV-2^5^, GenMark ePlex® SARS-CoV-2^6^, DnaNudge’s COVIDNudge test^7,8^, Abbott ID Now COVID-19^9^, and others^10^. The first three are fully automated systems for multiple sample testing with high sensitivity, e.g., it was reported that Xpert® Xpress achieved 98.3% sensitivity in their 46-minute PCR test^11^. However, the equipment is expensive and has a large footprint, which constrains scale-up testing and uses in low-resource settings. COVIDNudge is a lab-free RT–PCR platform that can detect seven viral targets (RdRp1, RdRp2, E-gene, N-gene, N1, N2, and N3) and one control (Ribonuclease P), with 94% sensitivity in a 90-min test^8^. However, the excessive number of wells required for one sample (72 wells) increases operation complexity and makes the test prone to handling and interpretation errors. FDA-approved POC tests employing isothermal amplification, such as Abbott ID Now COVID-19, can detect positive samples in as little as 5 min and negative results in 13 min using a small portable device^12^. Although this approach can obtain quick results, there have been reports of limited test accuracy and sensitivity^11,13^.

The use of microfluidics for diagnostic testing, particularly in a POC setting, is advantageous because it enables testing using a minute amount of sample, potentially enabling quicker detection and deployment with fewer reagents^14^. There have been numerous developments in microfluidic PCR systems^15^. Some designs provide multiple on-chip functions for sample preparation and detection. However, these systems are not modular and still have challenges to overcome for field deployment. Most PCR chips are made of glass, PDMS, or plastic, which have low thermal conductivity and are not suitable for applications requiring fast and precise temperature control within the microfluidic channel^16^. To overcome this limitation, reagents could be moved to regions on the chip that are preheated to the desired temperature for PCR. This was achieved either by using a flow-through system with multiple preheated zones^17,18^ or by cycling reagents between preheated zones^16^. However, these methods require high-precision programmable syringe pumps that make the system bulky and unsuitable for POC settings. Furthermore, multizone heating increases chip complexity, which poses a challenge for mass production. This issue is amplified if multiple reaction chambers (for example, to accommodate negative, positive, and internal controls) are needed on a chip. Microfluidics also enables the use of minute volumes, which reduces the volume of reagents needed. This is especially important in a pandemic that disrupts the supply chain of testing reagents. A microfluidic lab-on-chip has been demonstrated to be able to perform PCR on samples with volumes in the nanoliter range, down to 30 nL^19,20^. Although a small volume could reduce reagent usage, too low a volume would adversely affect the sensitivity of the test. This is more pronounced when attempting to detect asymptomatic cases in which the input sample has a very low titer of virus. These nanoliter-range chips cannot be scaled up to microliters due to their system design. In addition to PCR, portable lab-on-chip systems have been used to perform reverse transcription-loop mediated isothermal amplification (RT-LAMP) for diagnostic testing^21–26^. These systems cannot perform PCR due to their simplified thermal control units. Most cannot measure the dynamics of the reaction and capture only the end results. Furthermore, RT-LAMP has lower sensitivity than RT–PCR for COVID-19 detection^1,11,27,28^.

We developed a cost-effective patent-pending modular microfluidic RT–PCR and RT-LAMP platform, Epidax®^29^, for the rapid onsite diagnosis of SARS-CoV-2. This LEGO-like platform includes an on-chip temperature module, a detection module and analysis software and can be easily reconfigured to perform either COVID-19 screening by endpoint RT–PCR or RT-LAMP tests or confirmatory tests by real-time RT–PCR (RT–qPCR). We established its performance in detecting SARS-CoV-2 viruses from 81 and 43 clinical RNA extracts using our endpoint RT–PCR and RT–qPCR configured assays, respectively, to be comparable to the results obtained using a commercial system, but with half the amount of reagents used. In addition, we demonstrated rapid direct RT–PCR detection of SARS-CoV-2 viruses in 42 nasopharyngeal swab samples without RNA extraction, which reduced the sample-to-result testing time to an hour. Finally, we performed SARS-CoV-2 detection in 54 clinical RNA extracts using our reconfigured RT-LAMP platform. The modularity of the platform enables different assays to be performed depending on the use-case need and speed.

## Materials and methods

**Fig. 1:**
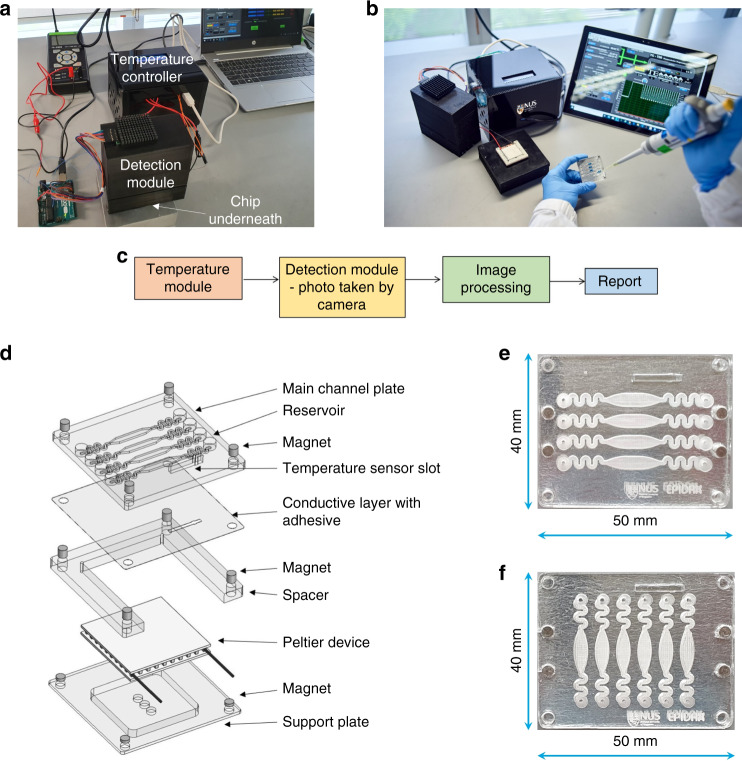
Epidax®, a microfluidic PCR platform for SARS-CoV-2 detection. **a** Epidax® in operation with the detection module placed on top of the microfluidic chip and temperature module (for real-time RT–PCR in a SARS-CoV-2 confirmatory test in which an image is taken at the end of each amplification cycle), and an image analysis package deployed using a laptop; **b** Epidax® in operation with the detection module detached (for endpoint RT–PCR or RT-LAMP in SARS-CoV-2 screening). Samples and reagents are loaded onto the chip and placed in the temperature module for processing based on required protocols; **c** Schematic method of performing the whole process of detection and diagnosis **d** Exploded view of the chip detailing its components. The channel plate containing the channel profile could be easily changed for new applications. Shown in the figure is a channel plate comprising four channels as four independent PCR chambers. This is a disposable component of the chip, whereas other components can be reusable. The channel plate consists of a slot for installing a temperature sensor for feedback control to the Peltier element; **e** A four-channel chip for RT–PCR. Each channel consists of an inlet feeding into a serpentine region, followed by a curved expansion and curved contraction region forming an eye-shaped chamber in which the reaction mix will reside, followed by another serpentine region leading to an outlet; **f** A six-channel chip for RT-LAMP, demonstrating the capability of testing more samples in one test.

The Epidax® system comprises a temperature module, a microfluidic chip, a detection module, and image processing and analysis software (see Fig. 1a–c). The chip can be easily mounted onto the temperature module and self-aligned by using magnets. The temperature of the sample in the chip is controlled over a period of time to enable an amplification reaction. The detection module comprises a CMOS camera to capture fluorescent images of the chip at defined time points (i.e., at start and end cycles for endpoint PCR or at the end of each amplification cycle for real-time PCR). The image processing and analysis software then analyzes the images taken. The reactions are quantified by the fluorescence intensity of the samples. The modularity of the platform offers flexibility in creating detection platforms for different sample types, detection targets, protocols, and deployment requirements. Here, we successfully employed Epidax® for various applications: COVID-19 screening by using endpoint RT–PCR or RT-LAMP, a confirmatory test using real-time RT–PCR on RNA extracts, and a screening or confirmation test using a direct RT–PCR assay on nasopharyngeal samples without RNA extraction.

### On-chip temperature module

The on-chip temperature module consists of the microfluidic chip, a Peltier element assembled with a spacer plate and a support plate (see Fig. 1d). Magnets are used for self-aligned assembly of the chip onto the thermal module, enabling quick changes of testing samples. Only the chip is disposable, whereas the entire module is reusable, which reduces the operating cost of the system.

The chip comprises a main channel plate and a thin aluminum adhesive film serving as a thermally conductive layer and enhancing the fluorescent signal collected by the detection module. Aluminum has a thermal conductivity of 200–237 W/mK, which is a few orders of magnitude higher than those of glass, PDMS, and plastic (generally less than 2 W/mK), which are traditionally used to fabricate microchannels. The low thickness and high thermal conductivity enable a high rate of heat transfer into and out of the microchannel. This is very important, as some PCR protocols require fast temperature cycling. By using the reflective aluminum film, light emitted by fluorescent samples will be reflected back up to the detection module, increasing the signal that can be collected compared to that obtained with a transparent wall (see Fig. S1 for the comparison of fluorescence intensity with different substrate materials, such as transparent glass and plastic). The excellent thermal and optical characteristics of the chip allow various applications, including both RT–PCR (cyclic thermal control) and RT-LAMP (isothermal control), for which the use of traditional plastic or PDMS chips with transparent walls is suboptimal.

The channel plate was fabricated using polymethyl methacrylate (PMMA) with dimensions of 50 mm × 40 mm (L × W). A sensor slot and holes for magnets were cut through using a CO_2_ laser (Universal laser systems). The temperature sensor used was MP-3176 (TE Technology, Inc.). The number of microchannels on the channel plate could be easily adjusted for various testing needs. Here, we demonstrate four or six microchannels on one chip, each used as an independent chamber, enabling multiple sample testing. For RT–PCR experiments, four identical channel profiles (see Fig. 1e) were engraved using a CO_2_ laser (Universal Laser Systems) to a depth of ~720 μm, and the total volume of each channel was ~60 μL. For RT-LAMP experiments, six-channel profiles (see Fig. 1f) were engraved to a depth of ~1.71 mm, giving a total channel volume of ~ 98 μL. Each channel consists of serpentine regions acting as flow resistors and an eye-shaped chamber in which the reaction mix will reside. During PCR, the temperature of the sample can be increased to over 90 °C. The flow resistors prevent the sample from moving out of the chamber due to the increase in the vapor pressure of the liquid sample. The serpentine region also acts as a mixer to enhance sample-reagent mixing when injected into the microchannel. The eye-shaped chamber, with its curvature along with the presence of oil, prevents the sample from breaking up into smaller portions under thermal cycling conditions. Additionally, the small reservoir at each inlet and outlet acts as a catchment to contain fluid (oil, etc.) that might flow out from the channel due to the vapor pressure caused by high temperatures. These designs can be easily customized by varying the depth and width of the channels to adapt to different volumes. Here, we use half of a standard PCR volume, which is 10–12.5 μL, for the on-chip detection of SARS-CoV-2.

The channel plate was soaked in RNase Away (Thermo Fisher), followed by thorough washing in nuclease-free water and air drying in a clean hood. An aluminum adhesive film with a thickness of 36 μm (Excel Scientific) was used as the conductive film and secured to the channel plate by manual application. The spacer was produced by laser cutting of PMMA. The support plate was machined by using copper material. The Peltier device was placed within the spacer, backed by the support plate. An optional layer of thermal paste (such as MX-4, Arctic) was applied to the contact surface between the chip and the Peltier device and to the contact surface between the Peltier device and the heatsink to enhance heat transfer.

### Detection module

The detection module comprises a slot for receiving the chip, a CMOS camera (Arducam OV5642) and an LED light source (see Fig. S1). It has dimensions of 110 mm × 60 mm × 90 mm and weighed ~250 g. The camera arrangement has a large field of view of ɸ 40 mm, which is larger than the sample area; thus, signals from all reaction channels can be captured simultaneously without mechanical movement of the light source and detection system, as in other commercial systems. This advantageously decreases the total detection time and thus the overall test time. The detection module further comprises a filter cube (including a dichroic mirror, an excitation filter, and an emission filter). The casing of the detector module is 3D printed with designated slots for the LEDs, filter cube, chip and camera using polylactic acid. For the FAM signal, LED light with a 492 nm peak wavelength, an excitation filter (446–486 nm), and an emission filter (cutoff at 520 nm) were used. For direct RT–PCR tests, two additional detection modules to detect the HEX signal (excitation peak at 538 nm, emission peak at 555 nm) and Texas Red signal (excitation peak at 596 nm, emission peak at 613 nm) were fabricated.

### Image processing and analysis software

We developed accompanying processing and analysis software for Epidax® to process the images taken by the detection module for real-time or endpoint detection. The in-house package can be run as a daemon for RT–qPCR experiments (for confirmatory SARS-CoV-2 test). As new images are captured at the end of each cycle, they are run through the procedure in Fig. S2a to generate the amplification plot and determine the Ct value. The ability to obtain these metrics nearly in real time is important in POC diagnostics, as it provides flexibility to hasten the time from sample to result. For example, there may be a clinical need to correlate Ct values to the presence of infectious virus (i.e., at the Ct of 20), and higher Ct values may indicate prolonged viral RNA shedding by a noninfectious individual^30^. If the testing scenario requires a positive diagnosis only for infectious patients, the test run can be programmed to stop when the detection threshold level is reached before cycle 20 or at a predetermined higher Ct value. This greatly enhances the detection efficiency.

This analysis package can also be run as a web application for endpoint RT–PCR (for COVID-19 screening) to provide quick screening for SARS-CoV-2, where the dynamics of the amplification are not of interest. In this mode, one image is obtained before an amplification reaction (Cycle 0) and compared with one image after a number of amplification cycles (e.g., Cycle 45). The intensity difference between these images (after processing as shown in Fig. S2e) can be used to conclude that the sample is positive for SARS-CoV-2 when it is higher than a screening threshold and negative otherwise.

### Test layout for detection of SARS-CoV-2

**Fig. 2:**
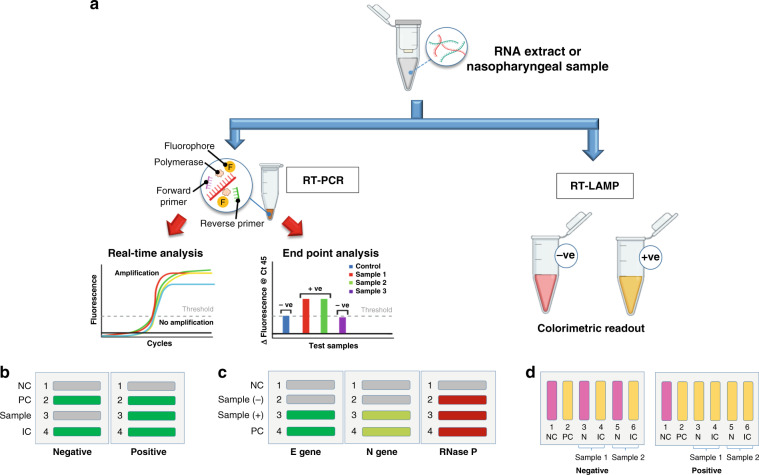
Test layouts for various SARS-CoV-2 detection methods. **a** Demonstration of different applications using Epidax® including: SARS-CoV-2 confirmatory test using RT–qPCR, and screening using endpoint RT–PCR and RT-LAMP; **b** Layout for RT–qPCR and endpoint RT–PCR test using 4-channel chip with singleplex RT–PCR reagent and RNA extract sample; and interpretation of test results. One sample was tested for each chip using FAM as fluorescence probe. Channels 1 and 2 for NC and PC, respectively; channel 3 for N1 gene of SARS-CoV-2; and channel 4 for human RP gene (IC). Green color represents positive detection while gray color represents a negative one; **c** Layout for direct RT–qPCR and endpoint RT–PCR tests using 4-channel chip with multiplex direct RT–PCR reagent and nasopharyngeal sample in UTM. Two samples (in channels 2 and 3, respectively) were tested in each chip (demonstrating the ability of Epidax® to test multiple samples); channels 1 and 4 for NC and PC. For each channel, three target genes were detected using different fluorescence probes, particularly E gene of SARS-CoV-2 (FAM), N gene of SARS-CoV-2 (HEX) and RNase P (Texas Red). Gray represents a negative signal, and other colors (green, light green, and red) represent a positive signal; **d** Layout for RT-LAMP using 6-channel chip with RNA extract; and interpretation of results. Two samples were tested for each chip. Channels 1 and 2 for NC and PC, respectively. Channels 3, 4, 5, and 6 tested the N gene of SARS-CoV-2 and human rActin gene (IC) of two different samples, respectively. Yellow color indicates a positive detection, and pink color a negative reaction.

Figure 2a shows various SARS-CoV-2 detection methods by using Epidax®, including (a) real-time RT–qPCR analysis for the SARS-CoV-2 confirmatory test; (b) endpoint RT–PCR; and (c) RT-LAMP for screening. In each application, experiments were performed on the Epidax® system and benchmarked against commercial PCR systems (i.e., the Bio–Rad C1000 Touch Thermal Cycler and CFX96 qPCR Detection system from Bio–Rad, referred to as “Bio–Rad” in this paper, and the ProFlex PCR system from Applied Biosystems, referred to as “ProFlex” in this paper).

Nasopharyngeal swabs were first collected from patients for clinical use and tested by local hospitals (National University Hospital and Ng Teng Fong General Hospital, Singapore); this test result is referred to as the “reference standard” in this paper. The leftover samples were heat-inactivated according to national biosafety guidelines and then sent to our lab for testing with Epidax®. The use of clinical samples was approved by the Domain Specific Review Board (DSRB 2020/00581 and 2020/00106) and the National University of Singapore Institutional Review Board (NUS-IRB-2020–666). All negative samples in UTM tested negative on the Cobas 6800 platform, which is a sample-to-result platform. All positive samples (POS01 to POS28) were collected in UTM, and then viral RNA was extracted by using abGenix viral DNA and RNA extraction on an abGenix viral DNA and RNA extraction system (AITbiotect), followed by RT–qPCR on a Bio–Rad system. The remaining negative and positive samples in the UTM were heat-inactivated by the corresponding hospitals at 60 °C for 30 min and 70 °C for 30 min, respectively, before being transferred to our lab. For our tests on Epidax®, viral RNA was first extracted from these inactivated leftover UTM samples using Qiagen QIAamp® Viral RNA Mini (Cat No 52906) according to the manufacturer’s spin protocol. The purified RNA samples were divided into aliquots in single-use tubes and stored in a -80 °C freezer for further testing with either RT–PCR reagents or RT-LAMP reagents.

Twenty of the clinical samples (RNA67 to RNA86) transferred to us were positive RNA extracts, which were extracted by Qiagen EZ1 Advanced platform and followed by RT–qPCR to confirm that they were positive for SARS-CoV-2. The Ct cutoff value used by the local hospital was 37. These remaining positive RNA extracts were not heat-inactivated before being transferred to our lab. They were stored in a −80 °C freezer.

Depending on the application, either RT–PCR, direct RT–PCR or RT-LAMP reagent was added to the RNA extract or the UTM sample. The reaction mix was then loaded onto the microfluidic chip using an oil sandwiching method (see Fig. S3), and the chip was then mounted onto the system for nucleic acid amplification.

### Singleplex RT–PCR assays on synthetic RNA and clinical RNA extracts from COVID-19 patients

For all RT–PCR tests using synthetic RNA or clinical RNA extract, endpoint or real-time monitoring of the fluorescence emission during amplification was performed on a 4-channel chip (see Fig. 1e). As shown in Fig. 2b, each chip tested one sample (N1 gene of SARS-CoV-2) and three controls, a negative control (NC), a positive control (PC) and an internal control (IC – the human RNase P gene). FAM was used as a fluorescence probe; green represents a sample with fluorescence (positive detection), while gray indicates low or no fluorescence (negative detection). Concurrently, a separate set of reaction mixtures was prepared and loaded onto the Bio–Rad system for benchmarking tests. At the end of the reaction, the mixtures from the Bio–Rad system were immediately loaded onto a clean empty chip for detection with the Epidax® detection module by using the oil sandwiching method. Note that for each experiment, an image of the empty chip was first taken, and no fluorescence was detected.

A commercially available one-step RT–PCR reagent (TaqPath™ 1-Step RT–qPCR Master Mix, Thermo Fisher Scientific) was used. The protocol on the Bio–Rad system comprises heating the sample to 50 °C and holding for 15 min, 92 °C for 2 min, followed by 45 cycles of 92 °C for 3 s and 55 °C for 30 s. A slightly modified protocol was used on Epidax® considering the temperature sensor accuracy: heating the sample to 51 °C for 15 min and then 92 °C for 2 min, followed by 45 cycles of 92 °C for 5 s and 56 °C for 30 s (see Fig. S4a). Primers for N1 and human RNase P genes with a FAM probe (2019-nCov CDC EUA Kit, Product code 10006770 from IDT) were used (see Table S1 for the sequences). NC represents nuclease-free water (HyPure™ Molecular Biology Grade Water, Nuclease free, HyClone™), while PC represents the 2019-nCoV N Positive Control at 10,000 copies per μL (IDT, Product Code 1000 6625). The 10 µL reaction mixture contained 0.75 µL of primer and probe, 2.5 µL RT–PCR master mix, 4.25 µL nuclease-free water, and 2.5 µL RNA template (see Table S2).

### Direct multiplex RT–PCR assay on nasopharyngeal samples in UTM from COVID-19 patients

For multiplex direct RT–PCR using nasopharyngeal samples (Fig. 2c), two samples were tested on each 4-channel chip, demonstrating the capability of testing multiple samples with our platform. For each channel, three target genes were detected by three fluorescence probes, i.e., the E gene (FAM), N gene (HEX) and RNase P (Texas Red). The benchmarking test was performed on a Bio–Rad system. The commercial direct RT–PCR reagent used was Resolute 2.0 (Advanced MedTech Holdings). The temperature profile on the Bio–Rad system included heating the sample to 55 °C and holding for 15 min, then 92 °C for 2 min, followed by 45 cycles of 92 °C for 3 s and 62 °C for 30 s. The protocol on Epidax® was heating the sample to 56 °C and holding for 15 min, then 92 °C for 2 min, followed by 45 cycles of 92 °C for 5 s and 63 °C for 30 s. The reaction mixture contained 9.65 µL of reaction mix, 0.35 µL of enzyme mix, and 2.5 µL of sample (see Table S3). The total reaction volume was 12.5 µL.

### RT-LAMP assays on synthetic RNA and clinical RNA extracts from COVID-19 patients

For all RT-LAMP tests, a six-channel chip (see Fig. 1f) was employed to test two samples per chip. No fluorescent dye was added, and the color change of the reaction mix was detected with the naked eye. Channels 1 and 2 were for NC and PC, respectively. Channels 3, 4, 5, and 6 tested the N gene of SARS-CoV-2 and the human rActin gene (IC) of two different samples. As shown in Fig. 2d, yellow indicates positive detection, and pink indicates negative detection. The benchmarking test was performed on a Proflex system.

A 10x primer mix was prepared according to Zhang et al.^31^: 16 µM forward inner primer (FIP) and backward inner primer (BIP) primers (each), 2 µM F3 and B3 primers (each), and 4 µM forward loop (LF) and backward loop (LB) primers (each). RT-LAMP was performed using primers for Gene N-A and rActin as shown in Table S4^32^, LAMP master mix (WarmStart Colorimetric Lamp 2x Master Mix, NEB, #M1800L), nuclease-free water (HyPure™ Molecular Biology Grade Water, Nuclease free, HyClone™), and a 2019-nCoV N positive control at 10,000 copies per μL (IDT, Product Code 1000 6625, stock concentration 200,000 copies per μL). The reaction mixture contained 2 µL of 10x primer mix, 10 µL of LAMP master mix, 5 µL of nuclease-free water and 3 µL of RNA template (see Table S5). The reaction was performed at 65 °C for 30 min on both Epidax® (see Fig. S4b) and Proflex systems.

## Results

### Analytical test for Epidax® benchmarking using synthetic RNA

**Fig. 3:**
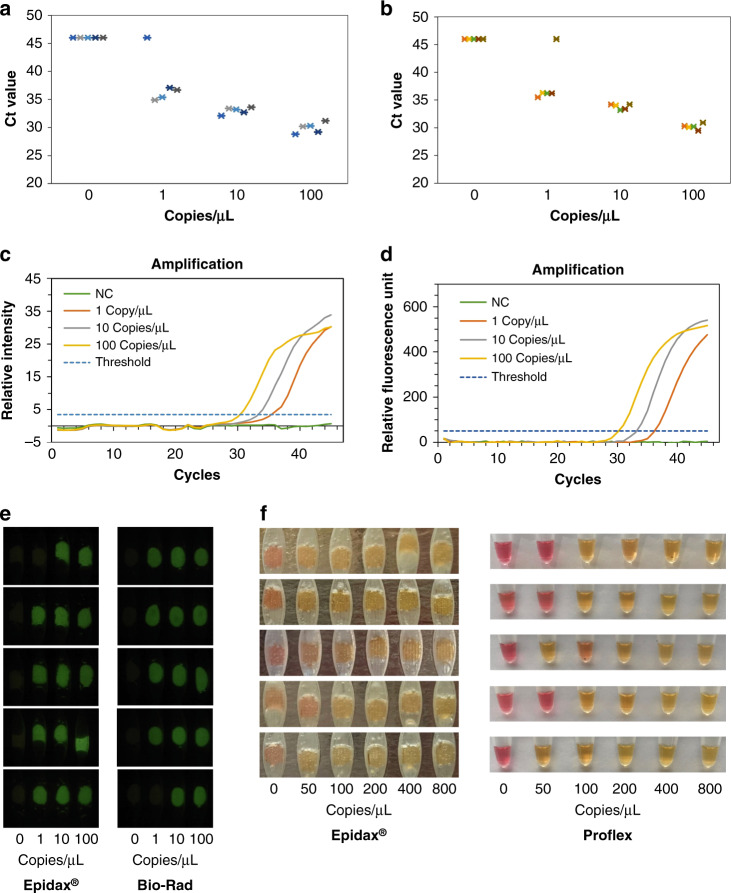
Epidax® RT–PCR and RT-LAMP benchmarking using synthetic RNA. **a** RT–PCR Ct values that were processed with Epidax®. **b** RT–PCR Ct values that were processed with Bio–Rad system. **c** Amplification plot of one run using Epidax®. **d** Amplification plot of one run using Bio–Rad system. **e** Fluorescence images of the samples after 45 PCR cycles (Cycle 45) on Epidax® and Bio–Rad systems; **f** Colorimetric images of the samples after RT-LAMP reaction on Epidax® and Proflex systems.

Tenfold serial dilutions of ultralow synthetic RNA control (AcroMetrix™ COVID-19 positive control, Cat No 954519, Thermo Fisher Scientific, stock concentration of 100 copies per μL quantified using Bio–Rad Droplet Digital™ PCR) were first prepared in nuclease-free water to obtain concentrations of 100, 10, and 1 RNA copies per μL. Subsequently, 2.5 µL of RNA sample at each concentration was mixed with 7.5 µL of PCR master mix and N1 primer for a reaction volume of 10 µL (refer to subsection test layout for detection of SARS-CoV-2 for details). Forty-five PCR cycles were performed on both the Epidax® and Bio–Rad systems. Real-time fluorescence images at the end of each cycle for all concentrations (100, 10, 1, and 0 RNA copies per μL) are shown in Movie S1. The experiments were repeated five times to show the reproducibility of the system. Figure 3a, b compare the Ct values for each concentration obtained from Epidax® and Bio–Rad. Figure 3 c, d shows amplification plots of one run processed by Epidax® and Bio–Rad, respectively, while Fig. 3e compares the fluorescence images of the samples after 45 cycles. On both Epidax® and Bio–Rad systems, 4 out of 5 replicates gave amplification for 1 copy per µL of synthetic SARS-CoV-2 RNA. This illustrates that Epidax® performs as well as Bio–Rad at the amplification of 1 RNA copy per µL. The limit of detection (LOD) of this assay using both Epidax® and Bio–Rad systems for both qPCR and endpoint PCR was 10 copies per μL (or 25 copies per reaction, with a 10 µL reaction volume), as all 5 replicates showed positive amplification. LOD is defined here as the lowest concentration detectable, with 95% of samples (true positive) showing positive detection.

**Fig. 4:**
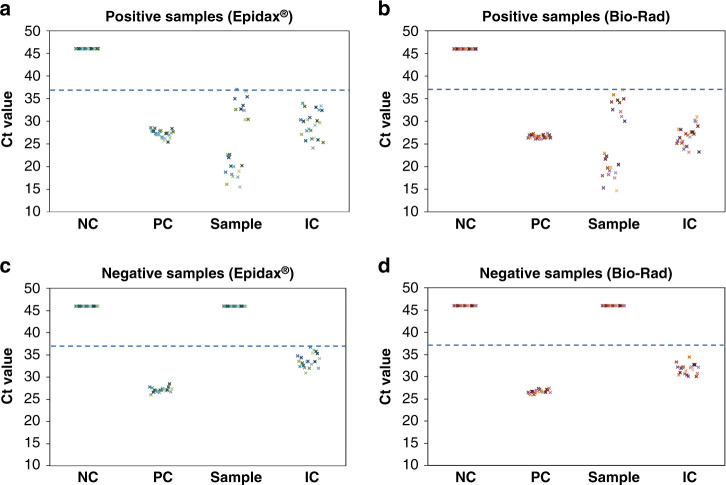
Ct values of 23 SARS-CoV-2 positive samples and 20 known negative samples obtained by Epidax® and benchmarked against Bio–Rad system. **a** Ct values of 23 positive samples detected by Epidax®; **b** Ct values of 23 positive samples detected by Bio–Rad; **c** Ct values of 20 negative samples detected by Epidax®; **d** Ct values of 20 negative samples detected by Bio–Rad. Ct values that were not detected are plotted as 46 for visualization purposes. Dashed line represents the cutoff line (Ct value of 37) used by local hospitals, i.e., Ct value below 37 is considered as a positive sample while negative otherwise. All controls including NC, PC, and IC of all samples performed as expected.

In RT-LAMP analytical tests, 2-fold serial dilutions of synthetic RNA carrying the gene N of SARS-CoV-2 (VR-3276SD, ATCC) were prepared in nuclease-free water to achieve concentrations of 800, 400, 200, 100, and 50 RNA copies per μL. Subsequently, 3 µL of RNA sample at each concentration was mixed with 17 µL of RT-LAMP master mix and primers for a 20 µL reaction (refer to subsection test layout for detection of SARS-CoV-2 for details). Isothermal amplification was performed on Epidax® and Proflex systems for 30 min at 65 °C. The experiments were repeated five times to show the reproducibility of the system. As shown in Figs. 3f, 4 out of 5 replicates gave amplification (i.e., yellow color) for 100 copies per μL of synthetic SARS-CoV-2 RNA on both systems. The LOD of this RT-LAMP assay was 200 copies per μL. This shows that Epidax® is as good as Proflex.

For the analytical specificity test, please refer to Appendix B, Tables S6–S8 in the Supplementary Information.

### SARS-CoV-2 detection of clinical RNA extracts using RT–qPCR

In this section, we demonstrated the use of Epidax® for SARS-CoV-2 detection with RT–qPCR for 43 clinical samples (23 known positives and 20 known negatives). For each clinical sample, 2.5 μL of purified RNA extract was mixed with 7.5 μL of singleplex RT–PCR master mix (refer to subsection test layout for detection of SARS-CoV-2 for reaction mix preparation). Forty-five cycles were run with each channel loaded with 10 μL of the reaction mix (see Fig. 2b for the test layout and result interpretation).

Real-time fluorescence images at the end of each cycle were captured and analyzed using our system (see Movies S2 and S3 for a negative and a positive sample, respectively). The Ct values of all three controls and samples processed using Epidax® were similar to those processed using the Bio–Rad system (see Fig. 4 – Ct values that were not detected are plotted as 46 for visualization purposes).

Applying a cutoff Ct value of 37, which was used by local hospitals, showed that all controls performed as expected. Among the samples (channel 3), all known positive samples and known negative samples were correctly identified using both the Epidax® and Bio–Rad systems, showing a Ct range of 14.7 to 37 for positive samples (Fig. 4a, b); and 46 (i.e., not detected) for negative samples (see Fig. 4c, d). Hence, we report comparable performance of Epidax® with the Bio–Rad system in a SARS-CoV-2 confirmatory test by using the RT–qPCR pathway.

### SARS-CoV-2 detection of clinical RNA extracts using endpoint RT–PCR tests

**Fig. 5:**
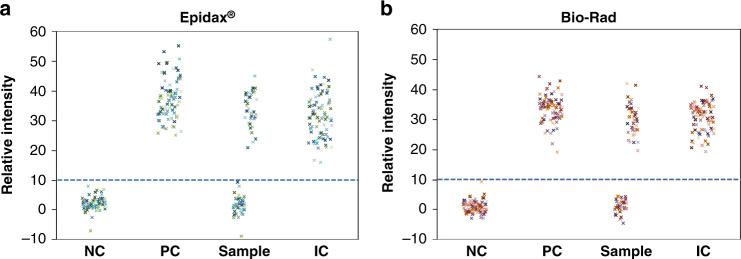
End-point RT–PCR analysis of 81 clinical samples (40 SARS-CoV-2 positives and 41 known negatives). **a** Detected by Epidax®; **b** Obtained by running 45 PCR cycles on Bio–Rad system, then the samples were loaded onto an empty chip and the fluorescence images captured using Epidax® detection module and the images similarly analyzed as that of Epidax®. By using the same threshold of relative intensity 10 (dashed line), 40 positive samples (with relative intensities in the range of 20–42), and 41 negative samples (all with relative intensities below 10) were accurately detected. All controls performed as expected: all NCs had relative intensities below 10, while all PCs and ICs had relative intensities above 10 (in the range of 20 to 44).

We performed an endpoint RT–PCR test with Epidax® on 81 clinical samples (40 SARS-CoV-2 positives and 41 known negatives). Here, the detection module was attached only to capture fluorescence images of the chip at Cycle 0 (before reaction) and Cycle 45 (at the end of thermal cycling – see Fig. S5). SARS-CoV-2 screening was conducted by intensity thresholding in which a sample was considered positive if the relative intensity was more than the screening threshold of 10 and negative otherwise. All 40 known positive samples and 41 known negative samples were correctly identified as positive and negative, respectively, by both the Epidax® and Bio–Rad systems (Fig. 5). Thus, the sensitivity and specificity of our test for SARS-CoV-2 were 100%, with false negative and false-positive rates of 0%. The sensitivity here is defined as true positives/(true positives + false negatives), and specificity is defined as true negatives/(true negatives + false-positives)^33^. Fig. S6 plots the relative intensities obtained for each channel separately. It is clear that the signals obtained from Epidax® are comparable to those from Bio–Rad.

Generally, for the purpose of COVID-19 screening in patients, Epidax® is as good as the Bio–Rad.

### Duration of tests

To investigate the duration of the endpoint RT–PCR test, the fluorescence intensities of 23 positive samples and 20 negative samples (used in the RT–qPCR test in Fig. 4) were measured at the ends of Cycles 37, 40, and 45. Fig. S7 plots the relative intensities of three controls and samples at Cycles 37, 40, and 45. We would need at least 40 cycles of PCR amplification for the endpoint RT–PCR test. At the end of Cycle 37, the intensities of some positive samples and ICs were still below the screening threshold. At the end of Cycle 40, there was only one false negative, and the other 42 samples were all correctly identified. The best performance was achieved at Cycle 45, where all samples were correctly identified as positive and negative.

### Rapid sample-to-result testing using direct RT–PCR

Significant savings in time and cost could be achieved with direct RT–PCR protocols in which RNA extraction is not needed. Here, we demonstrate quick sample-to-answer detection of SARS-CoV-2 using our platform with a multiplex direct RT–PCR kit on nasopharyngeal swab samples (see Fig. 2c for the test layout and subsection test layout for detection of SARS-CoV-2 for the reaction mix preparation). We performed both endpoint and real-time direct RT–PCR tests on 44 clinical samples in UTM (24 known positives and 20 known negatives). Two samples were tested on each chip, and the test duration from sample-to-result was approximately one hour.

For SARS-CoV-2 screening, our platform correctly identified 23/24 positive samples and 19/20 negative samples, while Bio–Rad accurately detected 24/24 positives and 17/20 negatives (see Fig. S8 and Table S9 for detailed results). The samples that were not correctly identified had Ct values of ~37, which was the cutoff value used by local hospitals.

For real-time detection, we collected only fluorescence images (for Ct value determination) from the N gene of SARS-CoV-2 (HEX), as shown in Table S10. Our system detected 23/24 positives and 20/20 negatives, while Bio–Rad detected 24/24 positive samples and 18/20 negative samples.

### SARS-CoV-2 detection using RT-LAMP

Epidax® was used to analyze 54 clinical RNA extracts (30 known positives, 24 known negatives) using RT-LAMP. The samples with RT-LAMP reagents and primer sets targeting the N gene of SARS-CoV-2 were loaded onto the chip for Epidax® and into tubes for the Proflex system (see Fig. 2d for test layout). Two samples were tested together in each run (see Fig. S9 for test results). Generally, the color change on the chip was comparable to the results obtained from the Proflex system. We observed that this RT-LAMP assay reliably detected positive samples with RT–qPCR Ct values of up to 30, which is in good agreement with the findings from the study of Dao Thi et al.^34^. However, for positive samples with Ct values above 30, we report 12 out of 16 samples with false negative results.

## Discussion

The current gold standard for SARS-COV-2 detection from nasopharyngeal swabs is based on RT–qPCR, which requires laboratory-based protocols for viral extraction. Most commercial COVID-19 diagnostic tests are intended for laboratory use on devices with large footprints and high costs and are not tailored for POC settings^35^. While they are automated and high throughput, they are not suitable for onsite deployment for screening purposes. POC systems often necessitate affordability, portability, and the ability to function in low resource settings.

Here, we demonstrated a modular platform with flexibility for multiple applications with a wide range of sensitivity (see Table 1 and Table S11 for the comparison of POC devices for nucleic acid detection of SARS-CoV-2). This POC device can be used for both SARS-CoV-2 screening and confirmatory testing. Our system accurately identified 40 positive and 41 negative RNA extracts using endpoint PCR (for SARS-CoV-2 screening) and 23 positive and 20 negative RNA extracts using real-time PCR (for SARS-CoV-2 confirmatory test), demonstrating complete agreement with a commercial platform (Bio–Rad system), with 100% sensitivity and 100% specificity. To enable time and cost savings that aided the expansion of testing, we employed an RNA extraction-free direct RT–PCR kit to correctly detect 23 positive and 19 negative nasopharyngeal samples using endpoint PCR and 23 positive and 20 negative nasopharyngeal samples using real-time PCR showing 95% specificity and 96% sensitivity. The system can also function as a colorimetric RT-LAMP assay, identified 30 positive and 24 negative RNA extracts with 100% specificity, and accurately detected 14/14 positive samples with Ct values below 30 (note that we do not calculate the overall sensitivity of the RT-LAMP assay here due to the limited spread of Ct values of the positive samples available – as shown in Fig. 4a, b, there are two distinct groups of Ct values).

**Table 1:** Clinical performance of Epidax® benchmarked against commercial PCR platforms.

			Reference standard			
			True positive	True negative	Sensitivity (%)	Specificity (%)
RT–qPCR	Epidax®	Positive	23	0	100	100
		Negative	0	20		
	Bio–Rad	Positive	23	0	100	100
		Negative	0	20		
End-point RT–PCR	Epidax® - Cycle 45	Positive	40	0	100	100
		Negative	0	41		
	Bio–Rad - Cycle 45	Positive	40	0	100	100
		Negative	0	41		
Direct RT–qPCR	Epidax®	Positive	23	0	96	100
		Negative	1	20		
	Bio–Rad	Positive	24	2	100	90
		Negative	0	18		
Direct End-point RT–PCR	Epidax® - Cycle 45	Positive	23	1	96	95
		Negative	1	19		
	Bio–Rad - Cycle 45	Positive	24	3	100	85
		Negative	0	17		
RT-LAMP	Epidax®	Positive	18	0	–	100
		Negative	12	24		
	Proflex	Positive	18	0	–	100
		Negative	12	24		

Note that the overall sensitivity of the RT-LAMP assay is not calculated due to the limited spread of Ct values of the positive samples available (as shown in Fig. 4a, b, there are two distinct groups of Ct values)

This POC platform demonstrated an LOD of 10 copies per μL (or 25 copies per reaction with a 10 µL reaction volume) with synthetic RNA for RT–PCR and 200 copies per μL of LOD for RT-LAMP. Studies on the viral load of SARS-CoV-2 in clinical samples indicate that this LOD corresponds to clinical needs^36,37^.

Using this platform, we established both screening and confirmatory tests for SARS-CoV-2. RT–PCR or RT-LAMP can be performed at desired temperature profiles, and the signal can be detected either by a fluorescence signal or color change. As demonstrated, the system has high configurability that enables it to run a variety of nucleic acid amplification protocols (RT–PCR, RT-LAMP, etc.) on various types of samples (RNA extracts, nasopharyngeal swabs in UTM, etc.). It can also be extended to run other protocols (such as recombinase polymerase amplification and direct RT-LAMP^34,38–41^) on other types of samples, such as saliva^42–44^, with minimal changes to the chip design.

To increase testing throughput, the chip can be extended to have more microchannels to process additional samples concurrently. The sample-to-answer processing time on our system is one hour for direct RT–PCR and could be further reduced by optimizing the PCR protocol. Our system is compact and deployable in a POC setting at ports of entry, field or community clinics, schools, nursing homes, workplaces, etc. Furthermore, the thermal unit and detection module can enable tests to be scaled up. For example, we can perform multiple endpoint RT–PCR or RT-LAMP tests in parallel, and result readout can be performed using the detection module. This detection module can easily function as a standalone unit for image-based detection and analysis. This is in contrast to the traditional laboratory qPCR system, which has integrated thermal and detection units and must be operated as a single unit.

Taken together, the system offers a modular, cost-effective, compact, and portable detection platform for point-of-care diagnostics in both clinical and field settings. This diagnostic platform provides a suite of capabilities that reduces the constraints and complexity of a laboratory-based facility and ensures greater success in the design of a more effective testing strategy for the community.

## Supplementary information

Modular microPCR system_supplementary info_revised_marked up

Movie S1_Limit of detection

Movie S2_SARS-CoV-2 detection of a negative sample

Movie S3_SARS-CoV-2 detection of a positive sample

IFU_CDC 2019-nCoV diagnostic rt-PCR

IFU_Resolute 2.0 Rev 1

## References

[1] Carter LJ (2020). Assay techniques and test development for COVID-19 diagnosis. ACS Cent. Sci..

[2] Vandenberg, O., Martiny, D., Rochas, O., van Belkum, A. & Kozlakidis, Z. Considerations for diagnostic COVID-19 tests. *Nat. Rev. Microbiol.*10.1038/s41579-020-00461-z (2020). 10.1038/s41579-020-00461-z PMC7556561 33057203

[3] Zhu N (2020). A novel coronavirus from patients with pneumonia in China, 2019. New Engl. J. Med..

[4] Cepheid. *US Food and Drug Administration, Coronavirus (COVID-19) Update: FDA Issues First Emergency Use Authorization For Point Of Care Diagnostic*. https://www.fda.gov/news-events/press-announcements/coronavirus-covid-19-update-fda-issues-first-emergency-use-authorization-point-care-diagnostic. Accessed 14 Nov 2021. (2020).

[5] Roche. *Roche’s cobas SARS-CoV-2 Test To Detect Novel Coronavirus Receives FDA Emergency Use Authorization and is Available in Markets Accepting the CE Mark*. https://diagnostics.roche.com/global/en/news-listing/2020/roche-receives-fda-emergency-use-authorization-for-cobas-sars-co.html. Accessed 14 Nov 2021. (2020).

[6] GenMark. *GenMark Receives FDA Emergency Use Authorization for its ePlex® SARS-CoV-2 Test*. https://ir.genmarkdx.com/news-releases/news-release-details/genmark-receives-fda-emergency-use-authorization-its-eplexr-sars. Accessed 14 Nov 2021. (2020).

[7] DnaNudge. *COVID Nudge: Rapid, Lab-free COVID-19 Test*. https://www.dnanudge.com/en/COVID-Nudge. Accessed 14 Nov 2021. (2020).

[8] Gibani MM (2020). Assessing a novel, lab-free, point-of-care test for SARS-CoV-2 (CovidNudge): a diagnostic accuracy study. Lancet Microbe.

[9] Abbott. *Abbott ID NOW COVID-19*. https://www.globalpointofcare.abbott/en/product-details/id-now.html. Accessed 14 Nov 2021. (2020).

[10] FDA. *In Vitro Diagnostics EUAs* https://www.fda.gov/medical-devices/coronavirus-disease-2019-covid-19-emergency-use-authorizations-medical-devices/vitro-diagnostics-euas#individual-molecular. Accessed 14 Nov 2021. (2020).

[11] Zhen W, Smith E, Manji R, Schron D, Berry GJ (2020). Clinical evaluation of three sample-to-answer platforms for detection of SARS-CoV-2. J. Clin. Microbiol..

[12] Abbott ID (2020). NOW COVID-19 test. 20 min and Dry Nasal Swabs in a New York City Academic Institution. J. Clin. Microbiol..

[13] Basu A (2020). Performance of Abbott ID Now COVID-19 rapid nucleic acid amplification test using nasopharyngeal swabs transported in viral transport media and dry nasal swabs in a new york city academic institution. J. Clin. Microbiol..

[14] Zhuang J, Yin J, Lv S, Wang B, Mu Y (2020). Advanced “lab-on-a-chip” to detect viruses – Current challenges and future perspectives. Biosens. Bioelectron..

[15] Ahrberg CD, Manz A, Chung BG (2016). Polymerase chain reaction in microfluidic devices. Lab Chip.

[16] Lee SH, Kim SW, Kang JY, Ahn CH (2008). A polymer lab-on-a-chip for reverse transcription (RT)-PCR based point-of-care clinical diagnostics. Lab Chip.

[17] Schneegass I, Brautigam R, Kohler JM (2001). Miniaturized flow-through PCR with different template types in a silicon chip thermocycler. Lab Chip.

[18] Hashimoto M (2004). Rapid PCR in a continuous flow device. Lab Chip.

[19] Kim H, Dixit S, Green CJ, Faris GW (2009). Nanodroplet real-time PCR system with laser assisted heating. Opt. Express.

[20] Ahrberg CD, Ilic BR, Manz A, Neužil P (2016). Handheld real-time PCR device. Lab Chip.

[21] Hui J (2018). Multiplex sample-to-answer detection of bacteria using a pipette-actuated capillary array comb with integrated DNA extraction, isothermal amplification, and smartphone detection. Lab Chip.

[22] Ganguli A (2020). Rapid isothermal amplification and portable detection system for SARS-CoV-2. Proc. Natl Acad. Sci. USA.

[23] Sun F (2020). Smartphone-based multiplex 30-minute nucleic acid test of live virus from nasal swab extract. Lab Chip.

[24] Xun G, Lane ST, Petrov VA, Pepa BE, Zhao H (2021). A rapid, accurate, scalable, and portable testing system for COVID-19 diagnosis. Nat. Commun..

[25] Nguyen HQ, Bui HK, Phan VM, Seo TS (2022). An internet of things-based point-of-care device for direct reverse-transcription-loop mediated isothermal amplification to identify SARS-CoV-2. Biosens. Bioelectron..

[26] Li N (2021). Multiplexed detection of respiratory pathogens with a portable analyzer in a “raw-sample-in and answer-out” manner. Microsyst. Nanoeng..

[27] Subsoontorn P, Lohitnavy M, Kongkaew C (2020). The diagnostic accuracy of isothermal nucleic acid point-of-care tests for human coronaviruses: A systematic review and meta-analysis. Sci. Rep..

[28] Giri B (2021). Review of analytical performance of COVID-19 detection methods. Anal. Bioanal. Chem..

[29] Lim, C. T. et al. Microfluidic chip and system. Patent Application No. 10202008413X – (2020).

[30] Gniazdowski, V. et al. Repeat COVID-19 molecular testing: correlation of SARS-CoV-2 culture with molecular assays and cycle thresholds. *Clin. Infect. Dis*. 10.1093/cid/ciaa1616 (2020). 10.1093/cid/ciaa1616 PMC7665437 33104776

[31] Zhang, Y. et al. Rapid molecular detection of SARS-CoV-2 (COVID-19) virus RNA using colorimetric LAMP. *medRxiv*10.1101/2020.02.26.20028373 (2020).

[32] Butler, D. J. et al. Host, viral, and environmental transcriptome profiles of the severe acute respiratory syndrome coronavirus 2 (SARS-CoV-2). *bioRxiv*10.1101/2020.04.20.048066 (2020).

[33] Parikh R, Mathai A, Parikh S, Chandra Sekhar G, Thomas R (2008). Understanding and using sensitivity, specificity and predictive values. Indian J. Ophthalmol..

[34] Dao Thi, V. L. et al. A colorimetric RT-LAMP assay and LAMP-sequencing for detecting SARS-CoV-2 RNA in clinical samples. *Sci. Transl. Med.*10.1126/scitranslmed.abc7075 (2020). 10.1126/scitranslmed.abc7075 PMC7574920 32719001

[35] 360dx. *Coronavirus Test Tracker: Commercially Available COVID-19 Diagnostic Tests*. https://www.360dx.com/coronavirus-test-tracker-launched-covid-19-tests. Accessed 14 Nov 2021. (2020).

[36] Pujadas, E. et al. SARS-CoV-2 Viral Load Predicts COVID-19 Mortality. *medRxiv*10.1101/2020.06.11.20128934 (2020). 10.1016/S2213-2600(20)30354-4 PMC7836878 32771081

[37] Jones, T. C. et al. An analysis of SARS-CoV-2 viral load by patient age. *medRxiv*10.1101/2020.06.08.20125484 (2020).

[38] Durner J (2020). Fast and simple high-throughput testing of COVID 19. Dent. Mater..

[39] Wee, S. K., Sivalingam, S. P. & Yap, E. P. H. Rapid direct nucleic acid amplification test without RNA extraction for SARS-CoV-2 using a portable PCR thermocycler. *Genes*10.3390/genes11060664 (2020). 10.3390/genes11060664 PMC7349311 32570810

[40] Ladha, A., Joung, J., Abudayyeh, O. O., Gootenberg, J. S. & Zhang, F. A 5-min RNA preparation method for COVID-19 detection with RT-qPCR. *medRxiv*10.1101/2020.05.07.20055947 (2020).

[41] Smyrlaki I (2020). Massive and rapid COVID-19 testing is feasible by extraction-free SARS-CoV-2 RT-PCR. Nat. Commun..

[42] Ranoa, D. R. E. et al. Saliva-based molecular testing for SARS-CoV-2 that bypasses RNA extraction. *bioRxiv*10.1101/2020.06.18.159434 (2020).

[43] Vogels, C. B. F. et al. SalivaDirect: a simplified and flexible platform to enhance SARS-CoV-2 testing capacity. *Med*10.1016/j.medj.2020.12.010 (2020). 10.1016/j.medj.2020.12.010 PMC7836249 33521748

[44] Wei, S. et al. Field-deployable, rapid diagnostic testing of saliva samples for SARS-CoV-2. *medRxiv*10.1101/2020.06.13.20129841 (2020).

