# Supplementary material for: Modular micro-PCR system for the onsite rapid diagnosis of COVID-19
Source: Microsyst Nanoeng. 2022 Jul 19;8:82. doi: 10.1038/s41378-022-00400-3 (PMC9294789; doi:10.1038/s41378-022-00400-3)
Supplement: Supplementary file 1 — Modular microPCR system_supplementary info_revised_marked up [file 41378_2022_400_MOESM1_ESM.docx]

# Supplementary information

Modular micro-PCR system for the onsite rapid diagnosis of COVID-19

Phuong Nguyen Quoc Mai^a,b^ (https://orcid.org/0000-0002-9802-7003),

Ming Wang^a^ (https://orcid.org/0000-0002-4639-9817),

Nelisha Ann Maria^a^ (https://orcid.org/0000-0002-3783-5023),

Adelicia Yongling Li^a^,

Hsih-Yin Tan^a^ (<https://orcid.org/0000-0002-3328-0489>),

Gordon Minru Xiong^a^,

Meng-Kwang Marcus Tan^c^,

Ali Asgar Saleem Bhagat^a,b^ (https://orcid.org/0000-0002-4695-8762),

Catherine W.M Ong^a,d*^ (<https://orcid.org/0000-0003-1103-0451>) – email: [catherine_wm_ong@nuhs.edu.sg](mailto:catherine_wm_ong@nuhs.edu.sg),

Chwee Teck Lim^a,b*^ (<https://orcid.org/0000-0003-4019-9782>) – email: [ctlim@nus.edu.sg](mailto:ctlim@nus.edu.sg)

^a^ Institute for Health Innovation and Technology (iHealthtech), National University of Singapore, MD6, 14 Medical Drive #14-01 Singapore 117599

^b^ Department of Biomedical Engineering, National University of Singapore, 4 Engineering Drive 3, Block 4, #04-08, Singapore 117583

^c^ Advanced MedTech, 2 Venture Drive, #23-18 Vision Exchange, Singapore 608526

^d^ Infectious Diseases Translational Research Programme, Department of Medicine, Yong Loo Lin School of Medicine, National University of Singapore, NUHS Tower Block, 1E Kent Ridge Road Level 11, Singapore 119228

* Corresponding authors

# Contents

# List of Supplementary Appendices

**Appendix A.** Details of image processing and signal analysis.

**Appendix B.** Analytical specificity test.

# List of Supplementary Tables

**Table S1.** Primers and probes used for RT-PCR experiments.

**Table S2.** Reaction mix preparation for each channel for singleplex RT-PCR experiments.

**Table S3.** Reaction mix preparation for each channel for multiplex direct RT-PCR test using Resolute 2.0 kit.

**Table S4**. RT-LAMP Primer Sequences.

**Table S5.** Master mix preparation for each channel for RT-LAMP experiments.

**Table S6.** RT-PCR Primers specificity check.

**Table S7.** RT-LAMP Forward Inner Primer homology check.

**Table S8.** RT-LAMP Backward Inner Primer homology check.

**Table S9.** Result interpretation of 22 SARS-CoV-2 positive and 20 known negative nasopharyngeal samples using multiplex direct RT-PCR kit on Bio-Rad and Epidax®. Intensity thresholding of 15, 11 and 3 were used for E gene (FAM), N gene (HEX) and RNase P (Texas Red) respectively.

**Table S10.** Ct values of 22 SARS-CoV-2 positive and 20 known negative nasopharyngeal samples using direct RT-PCR kit (N gene assay by HEX channel) on Bio-Rad and Epidax®.

**Table S11**. Comparison of POC devices for nucleic acid detection of SARS-CoV-2.

# List of Supplementary Figures

**Fig. S1.** Detection module.

**Fig. S2.** Image processing pipeline for RT-PCR.

**Fig. S3.** Loading sample on the chip.

**Fig. S4.** Temperature profile of (a) an RT-PCR reaction on Epidax® (b) an RT-LAMP reaction on Epidax®.

**Fig. S5.** Fluorescence images at cycle 45 obtained by Epidax® and Bio-Rad system using singleplex RT-PCR reagent.

**Fig. S6.** Relative intensities obtained for each sample on each channel separately (negative control or no template control, positive control, sample and internal control) from Epidax® (blue colour) and Bio-Rad system (orange colour). The signal from Epidax® system is comparable to that from Bio-Rad system.

**Fig. S7.** Epidax® end-point analysis for 23 SARS-CoV-2 positive samples and 20 known negative samples at various PCR cycle including Cycles 37, 40 and 45.

**Fig. S8.** Fluorescence images at cycle 45 of direct RT-PCR tests obtained by Epidax® and Bio-Rad systems.

**Fig. S9.** Clinical tests using RT-LAMP on Epidax® and Proflex.

# List of Supplementary Movies

**Movie S1.** Analytical test for Epidax® benchmarking using synthetic RNA. The movie shows real-time fluorescence images at the end of each amplification cycle for all concentrations. In each image, from left to right: 0, 1, 10, and 100 RNA copies per μL.

**Movie S2.** SARS-CoV-2 detection of a negative clinical RNA extract using RT-qPCR on Epidax® system. The movie shows real-time fluorescence images at the end of each cycle using Epidax®. In each image, from left to right: negative control, positive control, sample, and internal control.

**Movie S3.** SARS-CoV-2 detection of a positive clinical RNA extract using RT-qPCR on Epidax® system. The movie shows real-time fluorescence images at the end of each cycle using Epidax®. In each image, from left to right: negative control, positive control, sample, and internal control.

| **a**  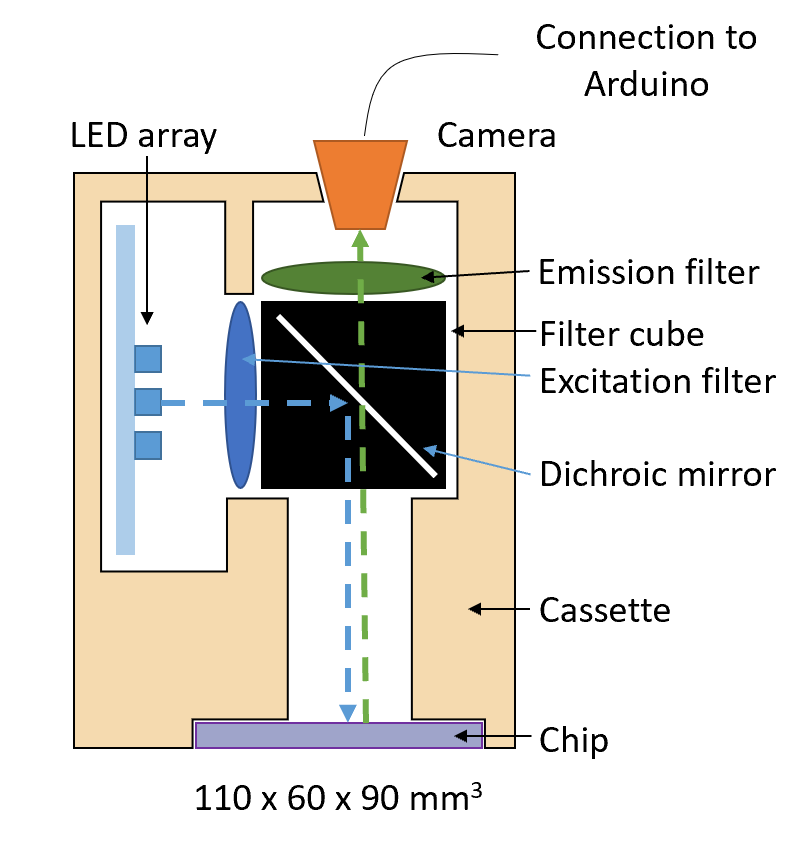 |
| --- |
| 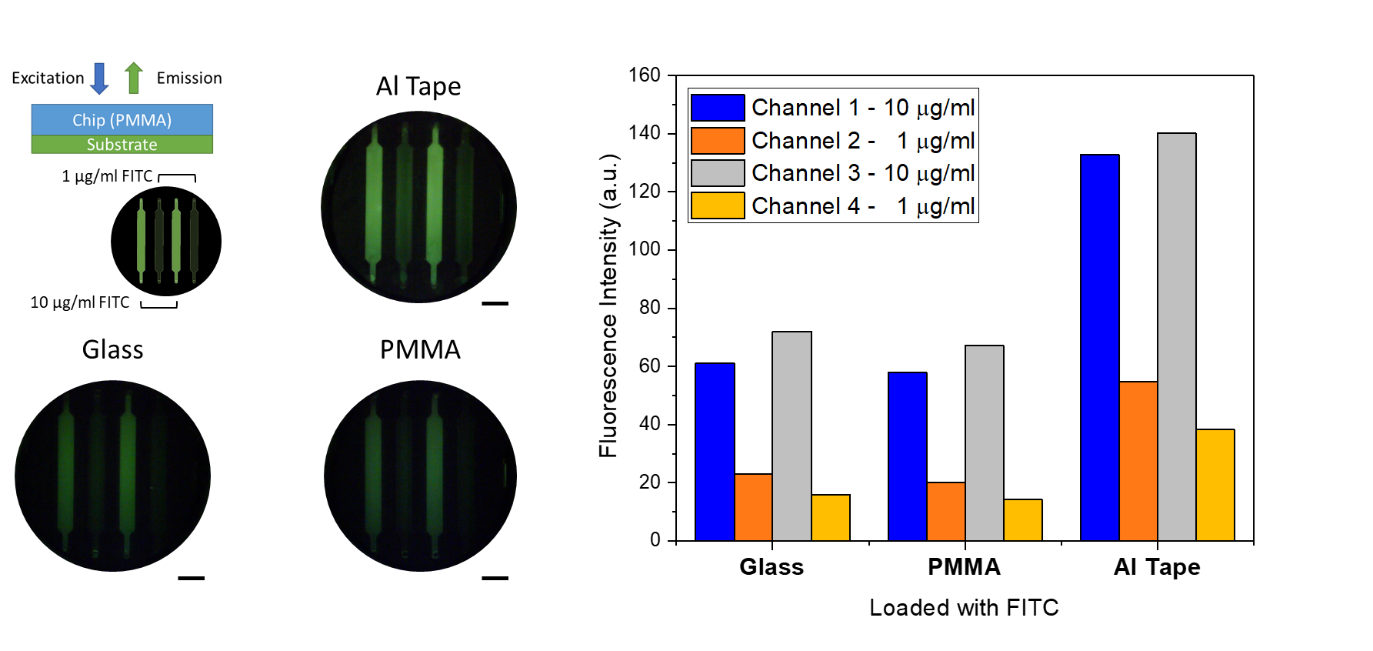  **b** |
| **Fig. S1. Detection module. a** Schematic of detection module; **b** Comparison of fluorescence intensity with different substrate materials. Left: Fluorescence images using FITC dye with concentration of 10 µg/ml and 1 µg/ml in microfluidic chip bound to different types of substrates. Scale bar: 5 mm. Right: Mean intensities of FITC in individual channels on glass, PMMA, and aluminium film. It is shown that a two-fold increase in fluorescence signal could be observed from the aluminium film in comparison to the glass and PMMA substrates. This is likely due to the reflection of the fluorescence signal by the aluminium surface, which may serve as a signal amplifier and hence help to increase the sensitivity of the system. |

## Appendix A. Details of image processing and signal analysis.

| a  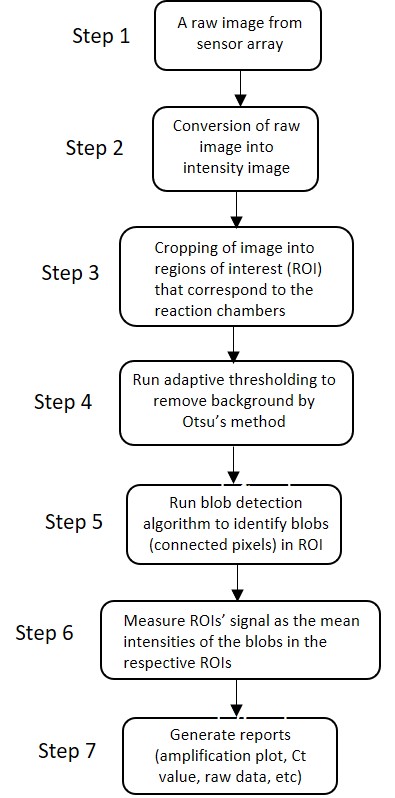 | e  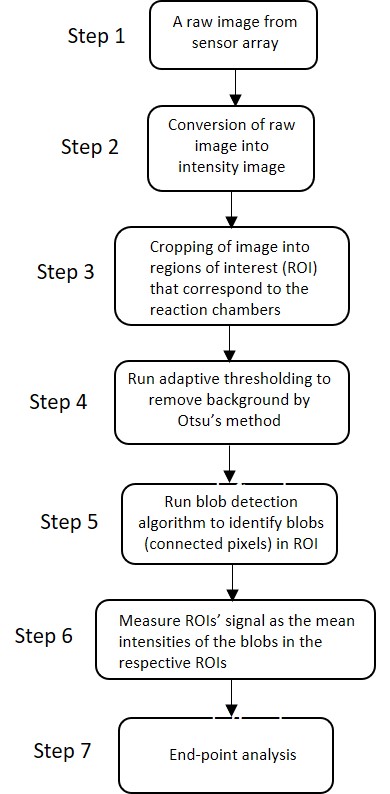 |
| --- | --- |
| b  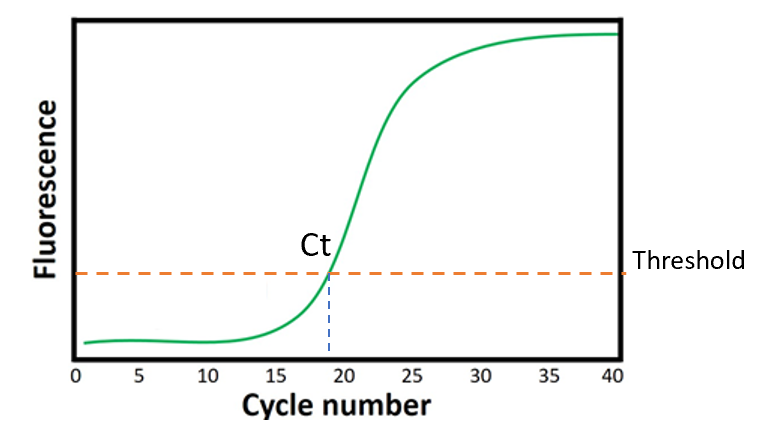 | |
| c  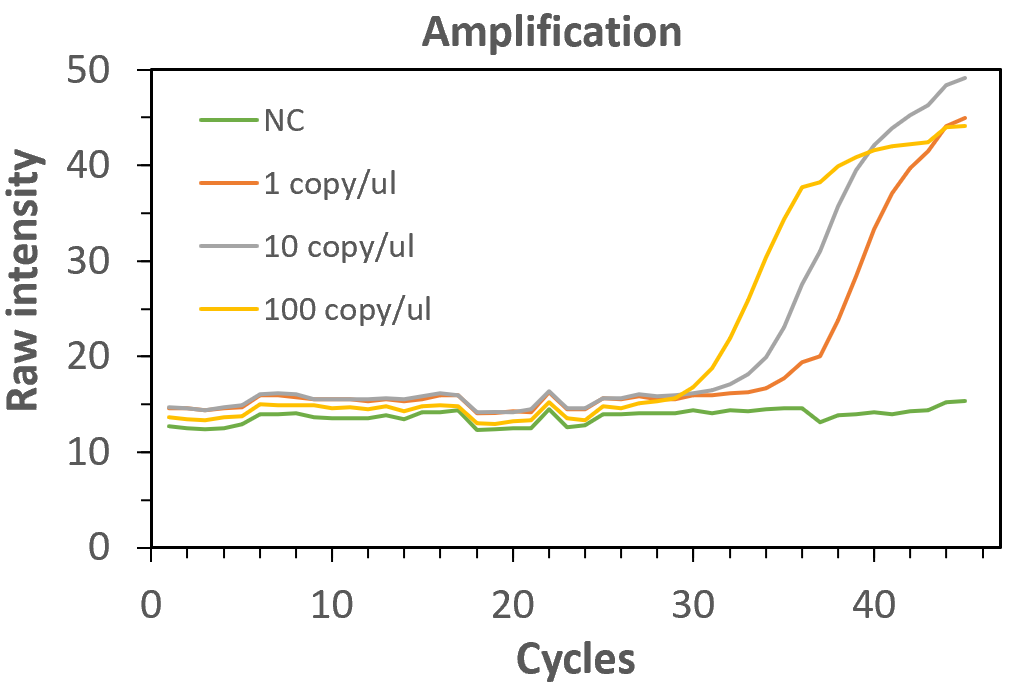 | d  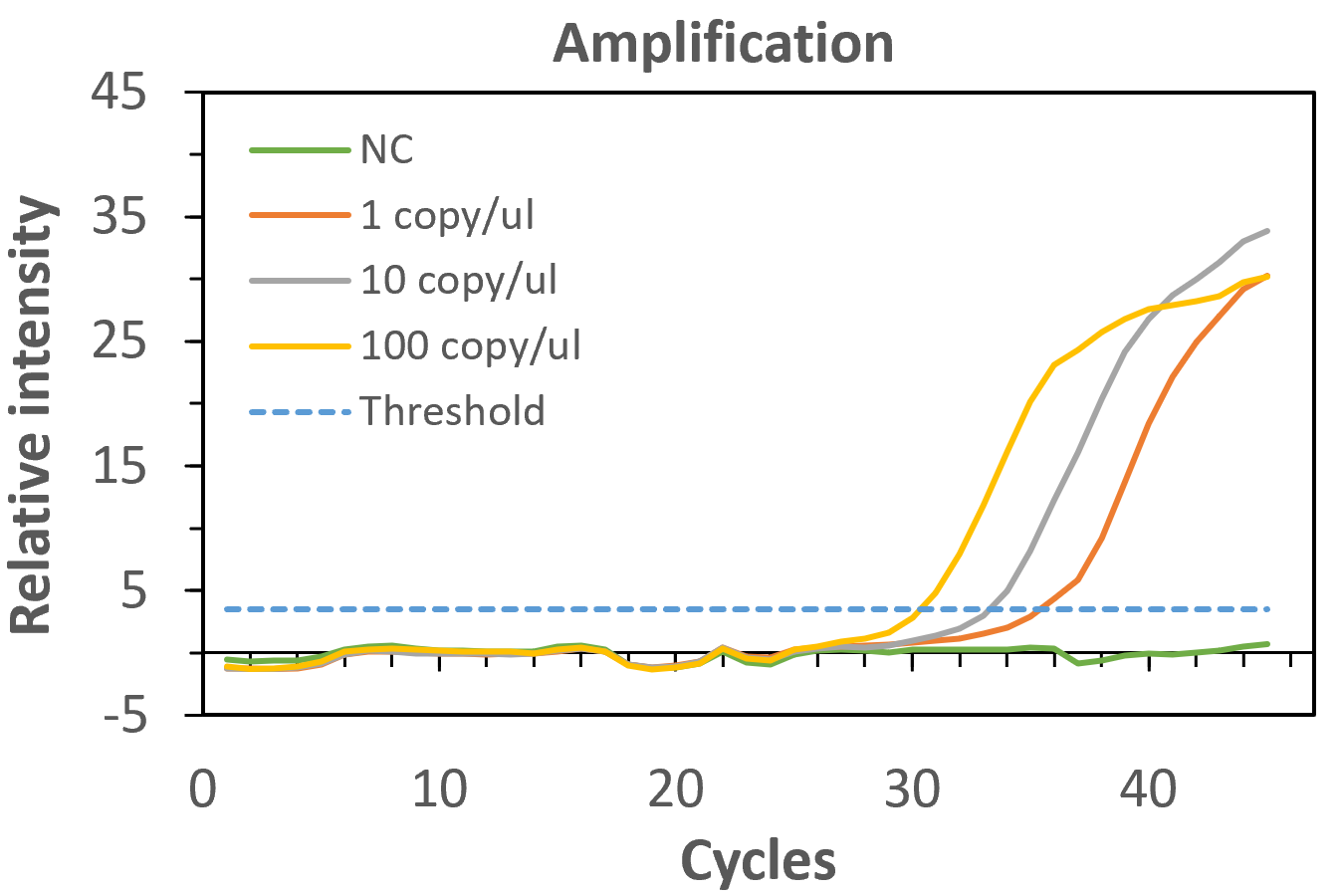 |
| **Fig. S2. Image processing pipeline for RT-PCR. a** Process flow chart for image processing for RT-qPCR; **b** Determination of Ct value from baseline subtracted amplification plot, i.e., the cycle at which fluorescence signal exceeds a pre-set threshold value (in this example, Ct = 19); **c** Amplification plot before baseline subtraction (the charts show raw intensities); **d** Amplification plot after baseline subtraction and smoothening (the plots show relative intensities); **e** Image processing pipeline for end-point RT-PCR analysis. | |

Image processing flow chart for qPCR is shown in Fig. S2a. In Step 1, the image processing pipeline takes a raw image taken from the CMOS camera after each amplification cycle as input. The raw image can be a multichannel image, such as RGB image, or a single channel image such as a greyscale image. In Step 2, for an RGB image, it is converted into an intensity image by taking the L2 norm of the image pixels. In Step 3, it is cropped into regions of interest (ROI) that correspond to the reaction channel, i.e., four regions of interest that correspond to four channels on the chip. In Step 4, for each ROI, background noise is removed by Otsu’s method. In Step 5, blob detection in each ROI is achieved by detecting connected pixels. The degree of connectivity of 2 was used, i.e., a bright pixel is considered connected to another bright pixel if the position of the second bright pixel is related to the position of the second pixel by offsetting the *x* or *y* position at most once in each direction. In Step 6, signals in each ROI are measured as mean intensity of the blobs. In Step 7, using these signals, raw amplification plots are generated.

Raw amplification plot of a sample is generated by plotting the mean intensity for ROI that corresponds to its reaction chamber for each reaction cycle. The amplification plot is then smoothened (using 1D Gaussian filter) and baseline subtracted in which the baseline is determined by fitting a linear regression line over the flat portion of the amplification plot before the exponential region. Ct value of a sample is determined from the baseline subtracted amplification plot as the cycle at which the intensity exceeds the Ct threshold set as illustrated in Fig. S2b. Amplification is considered to occur when there is an exponential growth region in the amplification plot.

For qPCR, the threshold value (or threshold line) is usually user-defined based on the application. It is adjusted to fall within the exponential phase. Here a fixed threshold value was defined and used for each assay. We first run an RT-PCR calibration experiment by using only the positive control as the template for amplification. The threshold value for Epidax® system was determined such that it would return the Ct value result close to that produced by Bio-Rad system. This is because Bio-Rad system was employed to benchmark our system. For example, for singleplex RT-PCR assay, the Ct values obtained on Bio-Rad system for 2019-nCoV N Positive Control at 10,000 copies per µL (IDT, Product Code 1000 6625) were 27.5 ± 0.5 with a threshold value of 50 (refer to the dash line in Fig. 3d of the main text). The threshold value (as mean intensity) used on Epidax® system to achieve such Ct values for the positive control was 3.5. Note that this threshold value was applied on the baseline subtracted amplification plot (please refer to the dash threshold line in Fig. 3c or Fig. S2d). After this calibration test, we used this threshold value to perform analytical performance and to test all clinical samples for singleplex RT-PCR.

For end point analysis, an image is processed similarly to steps 1 to 6 of qPCR procedure. The end point signal is determined by subtracting the intensities of end cycle (for e.g., Cycle 45) with the start cycle (Cycle 0) and compared against a threshold. For example, if the intensity difference between Cycle 45 and Cycle 0 of channel 3 (sample channel), is higher than 10, the clinical sample is concluded to be positive to SARS-CoV-2 while negative to SARS-CoV-2 otherwise. Here the threshold value (i.e. 10) was selected to be slightly above the highest relative intensity of all negative controls. This is because the negative control signal is the reference for no amplification of the RNA template.

| a  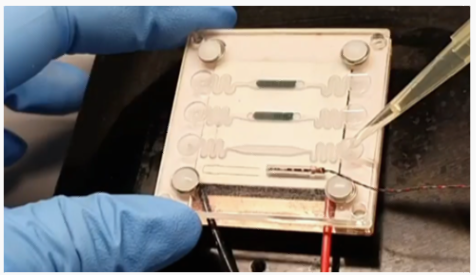 | b  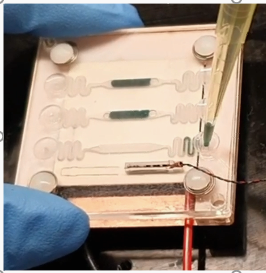 | c  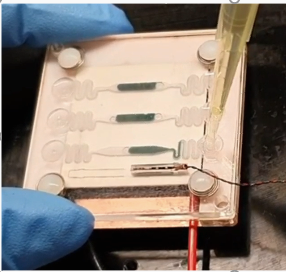 |
| --- | --- | --- |
| **Fig. S3. Loading sample on the chip. a** Add mineral oil; **b** Add reaction mix (in dark green colour for visualization purpose); **c** Add mineral oil. For RT-PCR, 22 µl of mineral oil was used for a 10 µL or 12.5 µL PCR reaction volume. The mineral oil was used to prevent evaporation issue during the denaturation step of the PCR cycle. For RT-LAMP, 40 µl of mineral oil and 20 µl of RT-LAMP reagent were loaded. | | |

| a  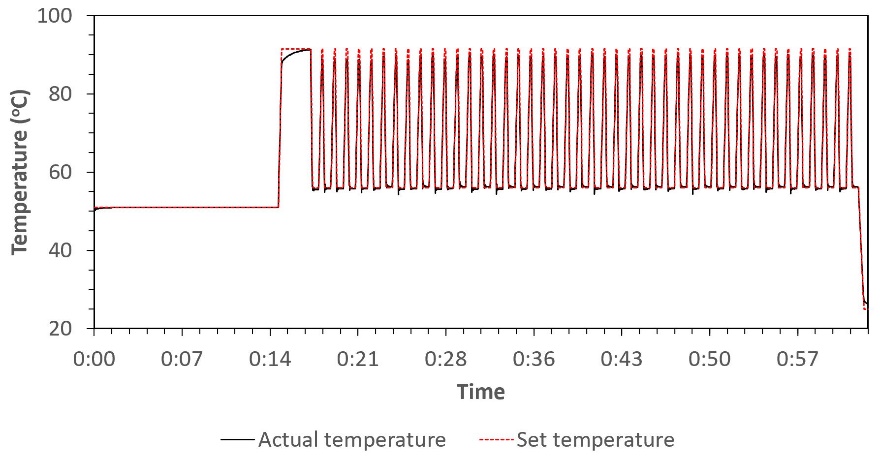 |
| --- |
| b  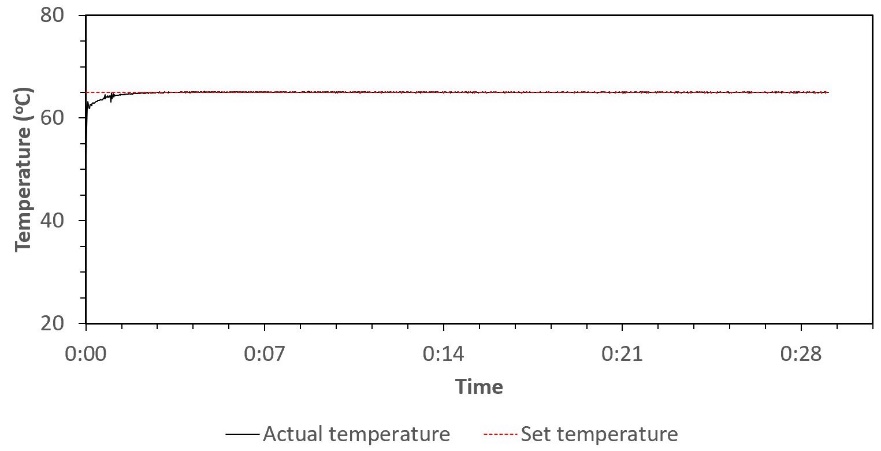 |
| **Fig. S4.** Temperature profile of (**a**) an RT-PCR reaction on Epidax® (**b**) an RT-LAMP reaction on Epidax®. The temperature set by the temperature controller is indicated by a dotted line, while the actual temperature detected by a control sensor is indicated by a solid line. The actual temperature matches that of the set temperature very closely, therefore is precise and suitable for a range of applications with a small ramp time. |

| a  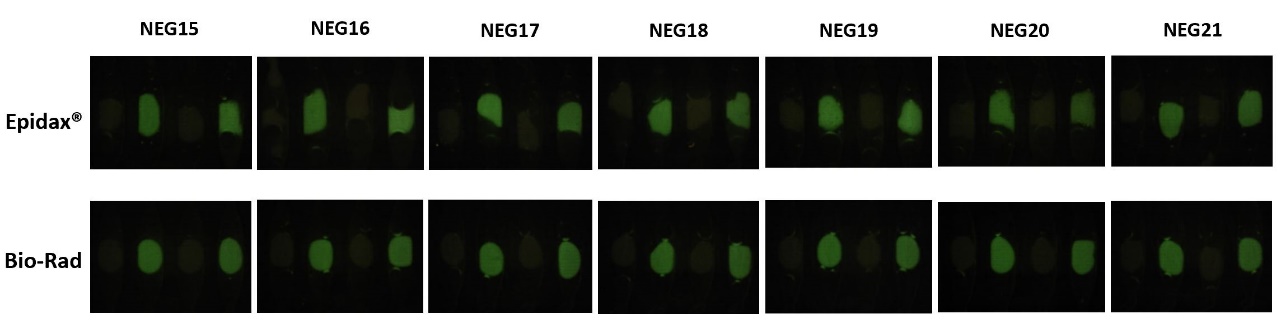 |
| --- |
| b  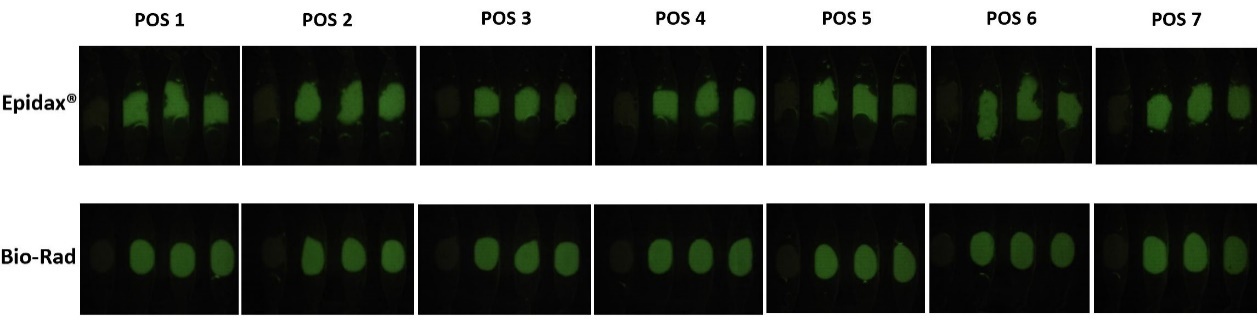 |
| **Fig. S5. Fluorescence images at cycle 45 obtained by Epidax® and Bio-Rad system using singleplex RT-PCR reagent.** **a** Seven negative samples; **b** Seven positive samples. For each image, from left to right: negative control, positive control, sample and internal control. The samples remained in the reaction chamber and did not break up. This shows that the flow resistors and oil provided high enough resistance to prevent sample from flowing or breaking up. Temperature profile of the 45 PCR cycles on Epidax® is shown in Fig. S4a. At the end of the reaction, the mixtures from Bio-Rad system were loaded onto an empty chip to be detected with our detection module. |

| a  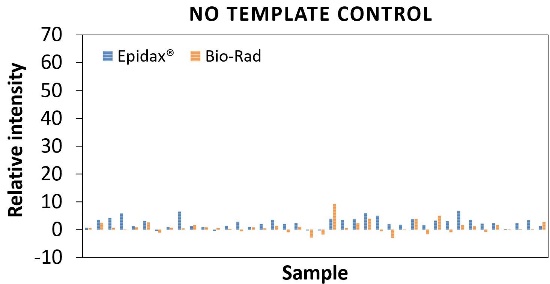 | 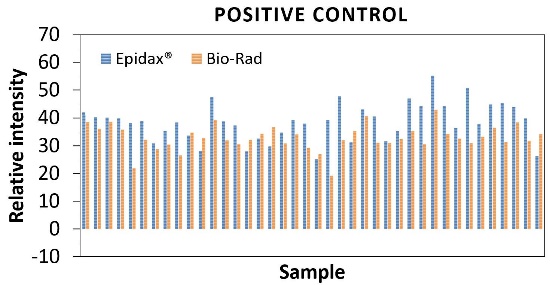 |
| --- | --- |
| 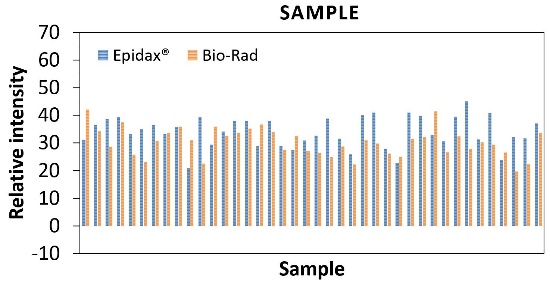 | 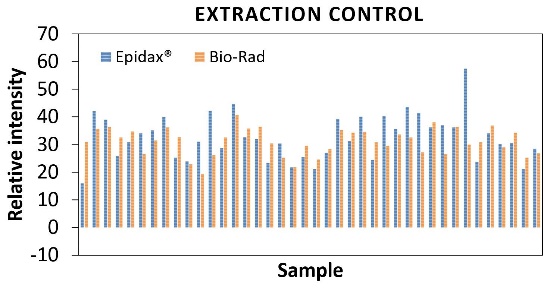 |
| b | |
| 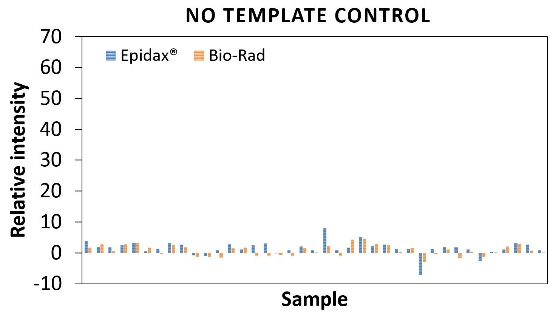 | 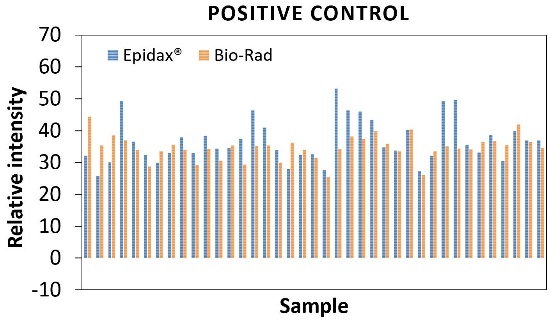 |
| 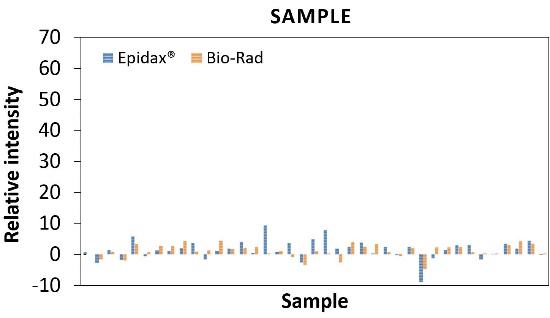 | 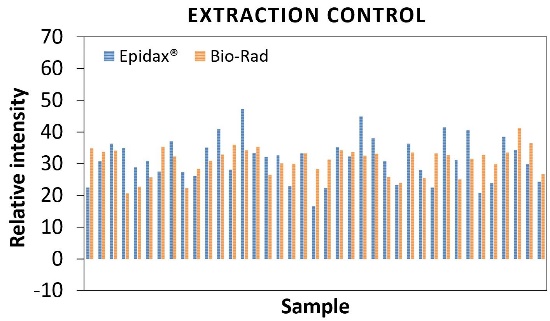 |
|  | |
| **Fig. S6. Relative intensities obtained for each sample on each channel separately (negative control or no template control, positive control, sample and internal control) from Epidax® (blue colour) and Bio-Rad system (orange colour). The signal from Epidax® system is comparable to that from Bio-Rad system. a** 40 SARS-CoV-2 positive samples; **b** 41 known negative samples. | |

| a | b |
| --- | --- |
| 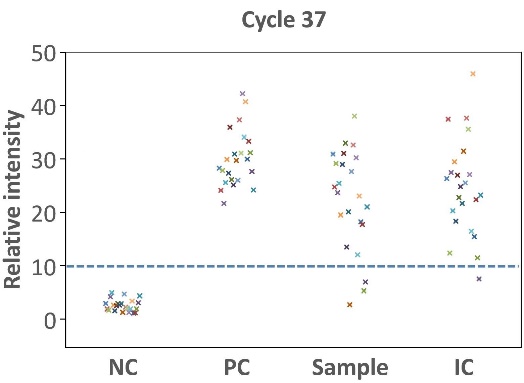 | 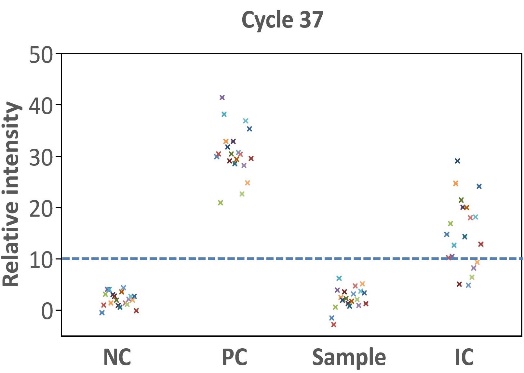 |
| 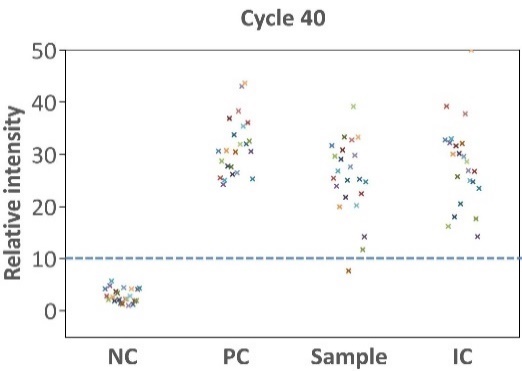 | 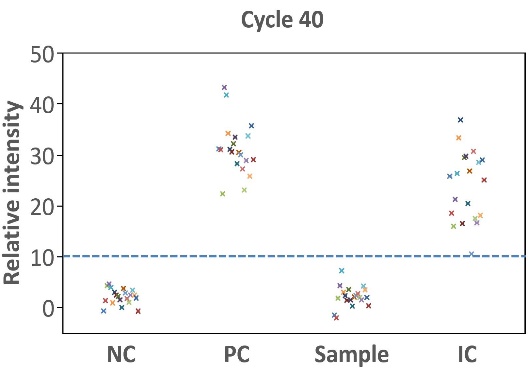 |
| 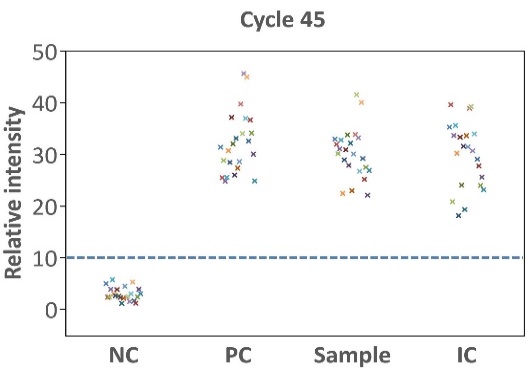 | 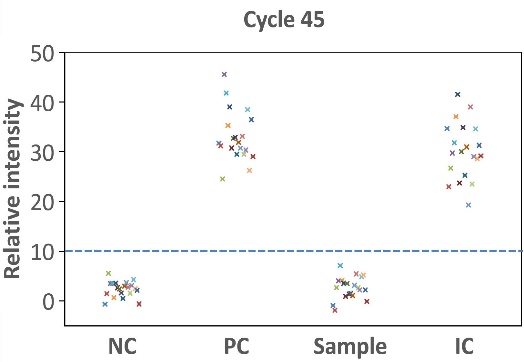 |
| **Fig. S7. Epidax® end-point analysis for 23 SARS-CoV-2 positive samples and 20 known negative samples at various PCR cycle including Cycles 37, 40 and 45. a** Positive samples; **b** Negative samples. Dashed line (at relative intensity 10) represents the single threshold applied to distinguish a negative sample from a positive one. | |

| a  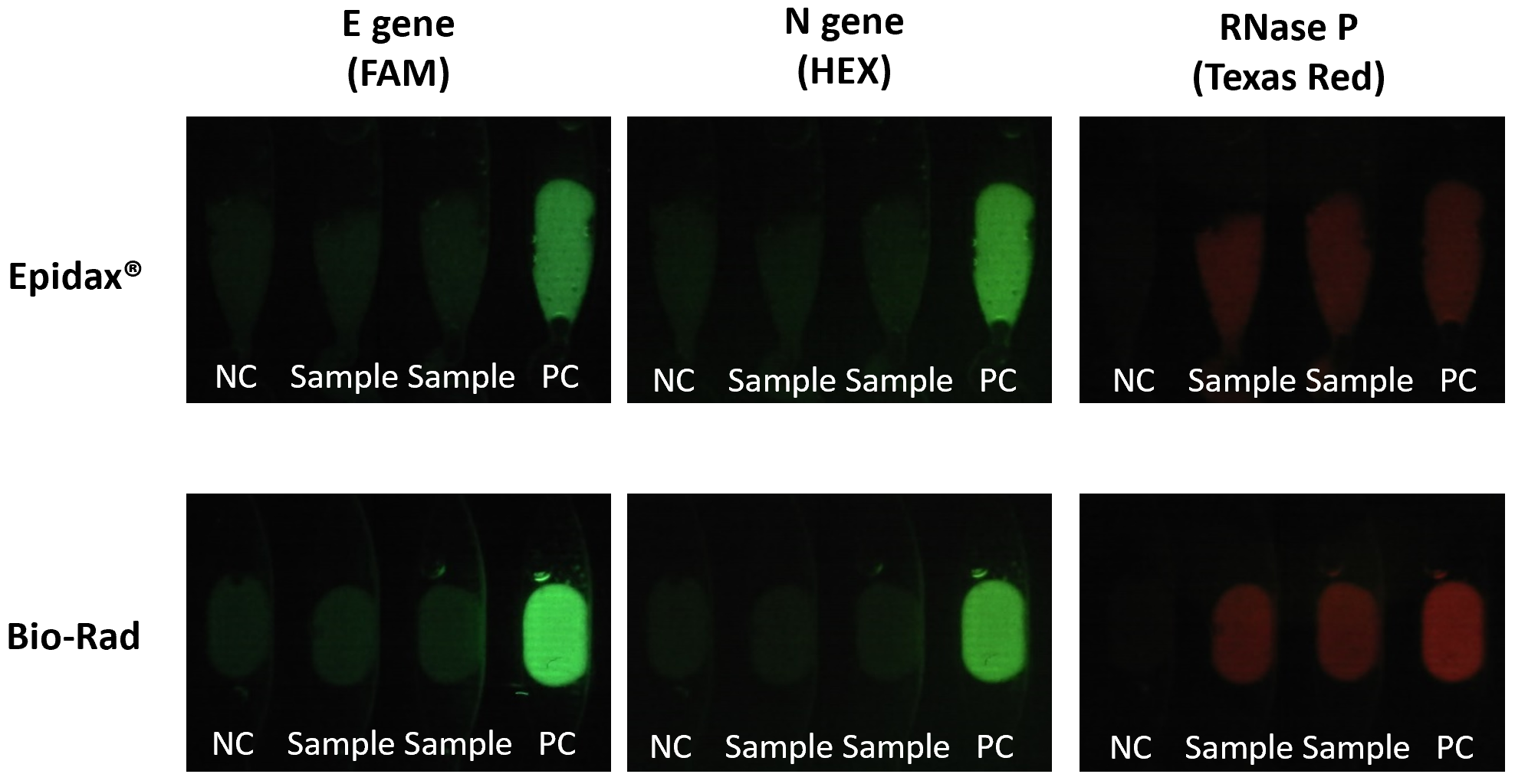 |
| --- |
| b  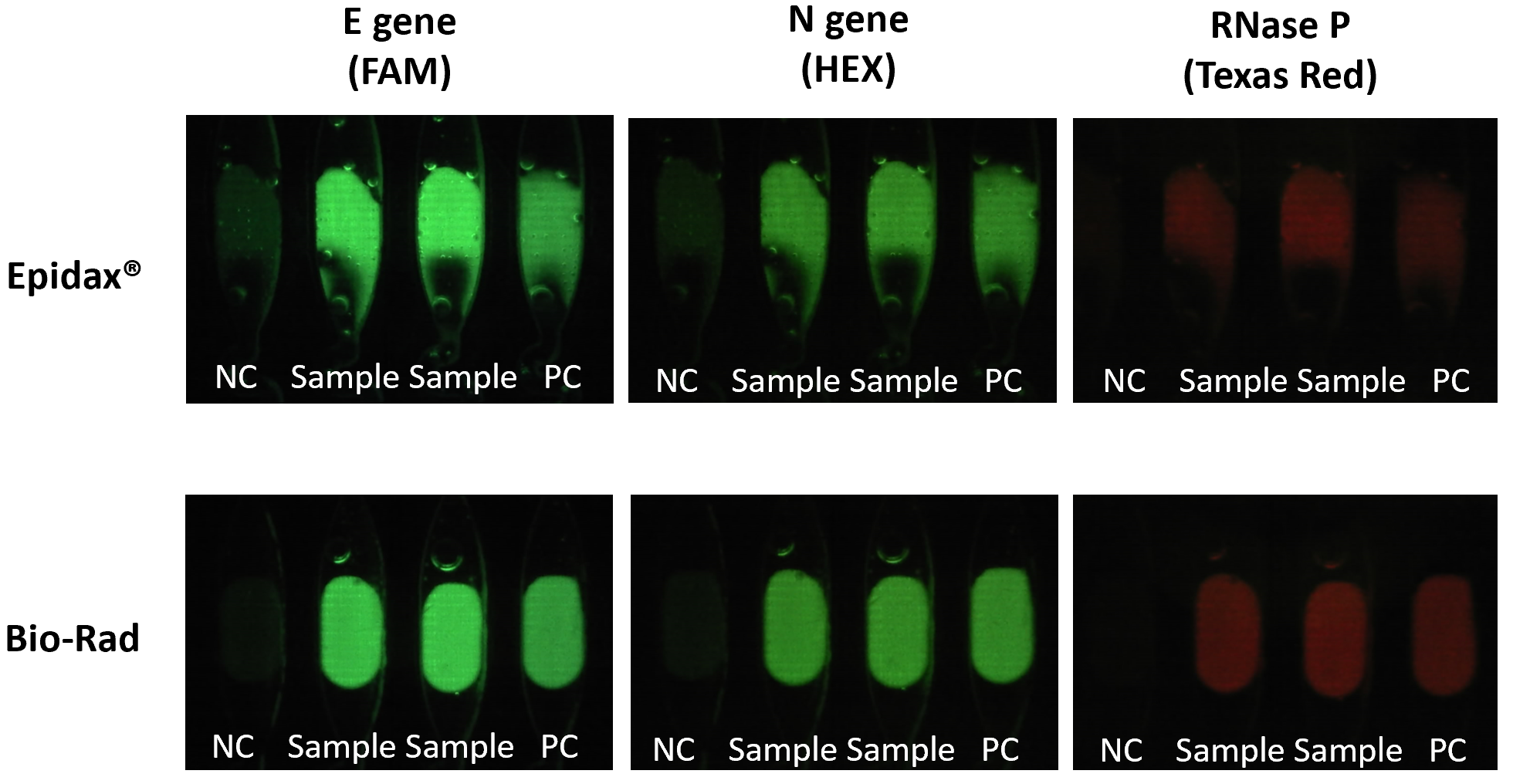 |
| **Fig. S8. Fluorescence images at cycle 45 of direct RT-PCR tests obtained by Epidax® and Bio-Rad systems.** For each experiment, two samples were tested on one chip; and for each sample, three assays were tested including E gene of SARS-CoV-2 (FAM), N gene of SARS-CoV-2 (HEX) and RNase P gene (Texas Red). In each image from left to right: negative control, sample 1, sample 2, and positive control. **a** Two negative sample; **b** Two positive samples. |

| 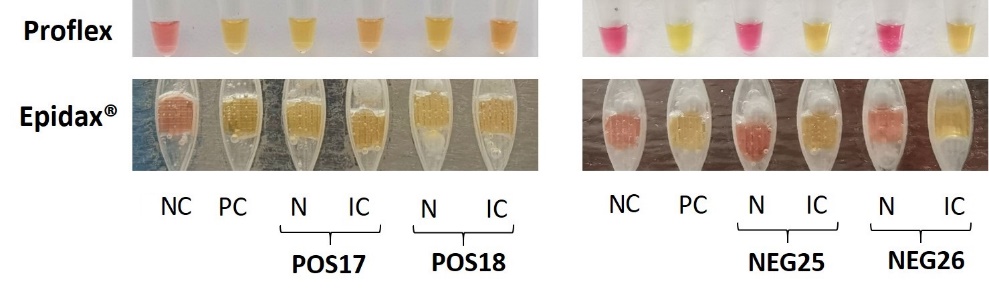 |
| --- |
| **Fig. S9. Clinical tests using RT-LAMP on Epidax® and Proflex.** Yellow indicates positive amplification (positive samples POS17 and POS18 as an example) and pink means no amplification (negative samples NEG25 and NEG26). |

**Table S1.** Primers and probes used for RT-PCR experiments.

| **Name** | **Description** | **Oligonucleotide Sequence (5’ to 3’)** |
| --- | --- | --- |
| SARS-CoV-2_N1-F | 2019 nCoV_N1 Forward | GAC CCC AAA ATC AGC GAA AT |
| SARS-CoV-2_N1-R | 2019 nCoV_N1 Reverse | TCT GGT TAC TGC CAG TTG AAT CTG |
| SARS-CoV-2_N1-P | 2019 nCoV_N1 Probe | ACC CCG CAT TAC GTT TGG ACC |
| RP-F | RP-F_RNaseP_Forward | AGA TTT GGA CCT GCG AGC G |
| RP-R | RP-R_RNaseP_Reverse | GAG CGG CTG TCT CCA CAA GT |
| RP-P | RP-P_RNaseP_Probe | TTC TGA CCT GAA GGC TCT GCG CG |

**Table S2.** Reaction mix preparation for each channel for singleplex RT-PCR experiments.

| **Channel No** | **Name** | **Reaction mix preparation** |
| --- | --- | --- |
| 1 | NC | 4.25 μl nuclease-free water + 2.5 μl Taqpath® master mix + 0.75 μl N1 primer/probe + 2.5 μl nuclease-free water |
| 2 | PC | 4.25 μl nuclease-free water + 2.5 μl Taqpath® master mix + 0.75 μl N1 primer/probe + 2.5 μl positive control |
| 3 | Sample | 4.25 μl nuclease-free water + 2.5 μl Taqpath® master mix + 0.75 μl N1 primer/probe + 2.5 μl RNA extract from sample |
| 4 | IC | 4.25 μl nuclease-free water + 2.5 μl Taqpath® master mix + 0.75 μl RP primer/probe + 2.5 μl RNA extract from sample |

**Table S3.** Reaction mix preparation for each channel for multiplex direct RT-PCR test using Resolute 2.0 kit.

| **Channel No** | **Name** | **Reaction mix preparation** |
| --- | --- | --- |
| 1 | NC | 9.65 μl reaction mix + 0.35 μl enzyme mix + 2.5 μl negative control |
| 2 | Sample 1 | 9.65 μl reaction mix + 0.35 μl enzyme mix + 2.5 μl sample 1 in UTM |
| 3 | Sample 2 | 9.65 μl reaction mix + 0.35 μl enzyme mix + 2.5 μl sample 2 in UTM |
| 4 | PC | 9.65 μl reaction mix + 0.35 μl enzyme mix + 2.5 μl positive control |

**Table S4**. RT-LAMP Primer Sequences.

Primers targeting the nucleocapsid (N-A) regions and human gene rActin were designed based on *Zhang* *et al.,* 2020 and Daniel J. Butler et al ^1^ and made by Integrated DNA Technologies.

| **Name** | **Oligonucleotide Sequence (5’ to 3’)** |
| --- | --- |
| Nucleocapsid Gene: (N, 5’-3’): | GeneN-A-F3 TGGCTACTACCGAAGAGCT  GeneN-A-B3 TGCAGCATTGTTAGCAGGAT  GeneN-A-FIP TCTGGCCCAGTTCCTAGGTAGTCCAGACGAATTCGTGGTGG  GeneN-A-BIP AGACGGCATCATATGGGTTGCACGGGTGCCAATGTGATCT  GeneN-A-LF GGACTGAGATCTTTCATTTTACCGT  GeneN-A-LB ACTGAGGGAGCCTTGAATACA |
| rActin (5′-3′) | ACTB-F3 AGTACCCCATCGAGCACG  ACTB-B3 AGCCTGGATAGCAACGTACA  ACTB-FIP GAGCCACACGCAGCTCATTGTATCACCAACTGGGACGACA  ACTB-BIP CTGAACCCCAAGGCCAACCGGCTGGGGTGTTGAAGGTC  ACTB-LoopF TGTGGTGCCAGATTTTCTCCA  ACTB-LoopB CGAGAAGATGACCCAGATCATGT |

**Table S5.** Master mix preparation for each channel for RT-LAMP experiments.

| **Channel No** | **Name** | **Master mix preparation** |
| --- | --- | --- |
| 1 | NC | 10 µL LAMP master mix + 5 µL nuclease free water + 2 µL of 10x primer mix for N gene + 3 µL nuclease-free water |
| 2 | PC | 10 µL LAMP master mix + 5 µL nuclease free water + 2 µL of 10x primer mix for N gene + 3 µL positive control |
| 3 | Sample 1 | 10 µL LAMP master mix + 5 µL nuclease free water + 2 µL of 10x primer mix for N gene + 3 µL RNA extract from sample 1 |
| 4 | IC for sample 1 | 10 µL LAMP master mix + 5 µL nuclease free water + 2 µL of 10x primer mix for rActin gene + 3 µL RNA extract from sample 1 |
| 5 | Sample 2 | 10 µL LAMP master mix + 5 µL nuclease free water + 2 µL of 10x primer mix for N gene + 3 µL RNA extract from sample 2 |
| 6 | IC for sample 2 | 10 µL LAMP master mix + 5 µL nuclease free water + 2 µL of 10x primer mix for rActin gene + 3 µL RNA extract from sample 2 |

## Appendix B. Analytical specificity test.

In this paper, we used three different sets of reagents and primers for RT-PCR and RT-LAMP, particularly

1. **Singleplex RT-PCR assay:** TaqPath^TM^ 1-Step RT-qPCR Master Mix and primers for N1 and Human RNase P genes with FAM probe (2019-nCov CDC EUA Kit, Product code 10006770 from IDT). These primers were tested and published by the Centers for Disease Control and Prevention, USA. Please refer to the instructions for use of CDC 2019-Novel Coronavirus (2019-nCoV) Real-Time RT-PCR Diagnostic Panel, Revision 3, page 39 – 41 for details of analytical specificity test (*in silico* analysis using Primer-BLAST and wet-lab testing). We further verified the cross-species specificity of the primers *in silico* using NCBI Primer-BLAST with database sequences of human coronaviruses, influenza, parainfluenza, adenoviruses and bacteria of significance (Table S6). The chosen primer pair was found to be specific only to SARS-CoV-2 virus sequences and not to any of the other microorganisms tested.
2. **RT-LAMP assay**: Commercial LAMP master mix (WarmStart Colorimetric Lamp 2x) were used. We further analysed *in silico* the primers designed by Zhang et al, 2020 for selection. Possible cross-species reactivity to other respiratory microbes was determined by homology checking of forward and backward inner primers (FIP & BIP) using NCBI BLASTn with database sequences of human coronaviruses, influenza, parainfluenza, adenoviruses and bacteria of significance. The selected primer set for the N gene (Gene N-A) exhibited significant homology (i.e. identical bases) of > 80% with either primer for only one microbe each, but of none where both the FIP and BIP primers have high homology to the same microbe species, which is required to carry out amplification (see Table S7 and Table S8).
3. **Multiplex direct RT-PCR assay**: Commercial direct RT-PCR reagent with primers for E gene and N gene (Resolute 2.0) were used. This assay was jointly developed by Singapore’s Agency for Science, Technology and Research (A*STAR) Diagnostics Development (DxD) Hub and DSO National Laboratories. It obtained the approval from Singapore’s Health Sciences Authority (HSA) and received the CE mark approval. Please refer to the instructions for use of Resolute 2.0, page 11 – 13 for details of analytical specificity test (*in silico* analysis and wet-lab testing).

## *In Silico* analysis of cross-species specificity of singleplex RT-PCR primers.

**Table S6. RT-PCR Primers specificity check.**

The specificity of the **SARS-CoV-2_N1** primer pair was checked against the NCBI reference collection of common respiratory microorganisms using Primer-BLAST. Positive specificity of the primer pair is determined when the results return amplicon products on the target microorganisms.

Forward primer: GAC CCC AAA ATC AGC GAA AT

Reverse primer: TCT GGT TAC TGC CAG TTG AAT CTG

| **Common microorganisms in respiratory infection** | **Specificity** |
| --- | --- |
| Human coronavirus (SARS-CoV-2) | Positive |
| Influenza A | Negative |
| Influenza B | Negative |
| Human Coronavirus NL63 | Negative |
| Human Coronavirus OC43 | Negative |
| Human Coronavirus HKU1 | Negative |
| Human Coronavirus 229E | Negative |
| Human Coronavirus (MERS-CoV) | Negative |
| Human Coronavirus (SARS-CoV) | Negative |
| Human parainfluenza virus 1 | Negative |
| Human parainfluenza virus 2 | Negative |
| Human parainfluenza virus 3 | Negative |
| Human parainfluenza virus 4a | Negative |
| Human parainfluenza virus 4b | Negative |
| Human adenovirus 1 | Negative |
| Human adenovirus E4 | Negative |
| Human adenovirus 4a | Negative |
| Human adenovirus 5 | Negative |
| Human adenovirus 7 | Negative |
| Haemophilus influenzae | Negative |
| Haemophilus parainfluenzae | Negative |
| Human rhinovirus | Negative |
| Legionella pneumophila | Negative |
| Mycobacterium tuberculosis | Negative |
| Mycoplasma pneumoniae | Negative |
| Klebsiella pneumoniae | Negative |
| Streptococcus pyogenes | Negative |
| Streptococcus pneumoniae | Negative |
| Staphylococcus aureus | Negative |
| Pseudomonas aeruginosa | Negative |
| Candida albicans | Negative |

## *In Silico* analysis of cross-species specificity of RT-LAMP primers.

**Table S7. RT-LAMP Forward Inner Primer homology check.**

The complementary sequence of the **Gene N-A-FIP primer** (bases in red) was checked against NCBI reference collection of the following microorganisms common in upper respiratory infections using BLASTn. The top hit in % homology with number of identical bases for each type of microorganism is displayed.

Gene N-A-FIP primer: TCTGGCCCAGTTCCTAGGTAGT**CCAGACGAATTCGTGGTGG**

|  | Top hit ID | No. of identical  bases (/19) | % Homology |
| --- | --- | --- | --- |
| Influenza A | [KU323253.1](https://www.ncbi.nlm.nih.gov/nucleotide/KU323253.1?report=genbank&log$=nuclalign&blast_rank=71&RID=ASS2R7VC016) | 12 | 63% |
| Influenza B | [CY237955.1](https://www.ncbi.nlm.nih.gov/nucleotide/CY237955.1?report=genbank&log$=nuclalign&blast_rank=1&RID=ASRKU9US016) | 11 | 58% |
| Human Cov NL63 | [MN306040.1](https://www.ncbi.nlm.nih.gov/nucleotide/MN306040.1?report=genbank&log$=nuclalign&blast_rank=97&RID=ASRMM37G014) | 9 | 47% |
| OC43 | [AY585229.1](https://www.ncbi.nlm.nih.gov/nucleotide/AY585229.1?report=genbank&log$=nuclalign&blast_rank=4&RID=ASRMM37G014) | 14 | 74% |
| HKU1 | [KY674943.1](https://www.ncbi.nlm.nih.gov/nucleotide/KY674943.1?report=genbank&log$=nuclalign&blast_rank=223&RID=ASRMM37G014) | 9 | 47% |
| 229E | [N369046.1](https://www.ncbi.nlm.nih.gov/nucleotide/MN369046.1?report=genbank&log$=nuclalign&blast_rank=89&RID=ASRMM37G014) | 9 | 47% |
| MERS-CoV | [MG596803.1](https://www.ncbi.nlm.nih.gov/nucleotide/MG596803.1?report=genbank&log$=nuclalign&blast_rank=1&RID=ASSEKG69014) | 11 | 58% |
| SARS-CoV | [JX163928.1](https://www.ncbi.nlm.nih.gov/nucleotide/JX163928.1?report=genbank&log$=nuclalign&blast_rank=45&RID=ASSJUT5W01R) | 15 | 79% |
| Human parainfluenza virus 1 | [AF457102.1](https://www.ncbi.nlm.nih.gov/nucleotide/AF457102.1?report=genbank&log$=nuclalign&blast_rank=2&RID=ASU0XBTF01R) | 10 | 53% |
| Human parainfluenza virus 2 | [AB176531.1](https://www.ncbi.nlm.nih.gov/nucleotide/AB176531.1?report=genbank&log$=nuclalign&blast_rank=34&RID=ASU1ZCM0016) | 9 | 47% |
| Human parainfluenza virus 3 | [KJ672618.1](https://www.ncbi.nlm.nih.gov/nucleotide/KJ672618.1?report=genbank&log$=nuclalign&blast_rank=2&RID=ASU225UF014) | 11 | 58% |
| Human parainfluenza virus 4a | [AB543336.1](https://www.ncbi.nlm.nih.gov/nucleotide/AB543336.1?report=genbank&log$=nuclalign&blast_rank=1&RID=ASU7K0EF016) | 9 | 47% |
| Human parainfluenza virus 4b | [MN306058.1](https://www.ncbi.nlm.nih.gov/nucleotide/MN306058.1?report=genbank&log$=nuclalign&blast_rank=1&RID=ASU7VK8V014) | 8 | 42% |
| Human adenovirus 1 | [MH183293.1](https://www.ncbi.nlm.nih.gov/nucleotide/MH183293.1?report=genbank&log$=nuclalign&blast_rank=1&RID=ASUBN97M014) | 8 | 42% |
| Human adenovirus E4 | [KX384957.1](https://www.ncbi.nlm.nih.gov/nucleotide/KX384957.1?report=genbank&log$=nuclalign&blast_rank=3&RID=ASUB9M21014) | 9 | 47% |
| Human adenovirus 4a | [AB679756.1](https://www.ncbi.nlm.nih.gov/nucleotide/AB679756.1?report=genbank&log$=nuclalign&blast_rank=1&RID=ASUC8F5W014) | 8 | 42% |
| Human adenovirus 5 | [KX868466.2](https://www.ncbi.nlm.nih.gov/nucleotide/KX868466.2?report=genbank&log$=nuclalign&blast_rank=1&RID=ASUF6XYJ01R) | 8 | 42% |
| Human adenovirus 7 | [MK492257.1](https://www.ncbi.nlm.nih.gov/nucleotide/MK492257.1?report=genbank&log$=nuclalign&blast_rank=1&RID=ASUFB9G901R) | 8 | 42% |
| Haemophilus influenzae | [CP044497.1](https://www.ncbi.nlm.nih.gov/nucleotide/CP044497.1?report=genbank&log$=nuclalign&blast_rank=1&RID=ASUJ5ZCH01R) | 13 | 68% |
| Haemophilus parainfluenzae | [CP035368.2](https://www.ncbi.nlm.nih.gov/nucleotide/CP035368.2?report=genbank&log$=nuclalign&blast_rank=1&RID=ASUJE6MK01R) | 13 | 68% |
| Human rhinovirus | [MN749148.1](https://www.ncbi.nlm.nih.gov/nucleotide/MN749148.1?report=genbank&log$=nuclalign&blast_rank=1&RID=ASUKE7Z201R) | 11 | 58% |
| Legionella pneumophila | [LR134176.1](https://www.ncbi.nlm.nih.gov/nucleotide/LR134176.1?report=genbank&log$=nuclalign&blast_rank=1&RID=ASURK66401R) | 13 | 68% |
| Mycobacterium tuberculosis | [CP049108.1](https://www.ncbi.nlm.nih.gov/nucleotide/CP049108.1?report=genbank&log$=nuclalign&blast_rank=1&RID=ASUS1FPR01R) | 15 | 79% |
| Mycoplasma pneumoniae | [CP039761.1](https://www.ncbi.nlm.nih.gov/nucleotide/CP039761.1?report=genbank&log$=nuclalign&blast_rank=1&RID=ASUSBVYD01R) | 14 | 74% |
| Klebsiella pneumoniae | [CP042977.1](https://www.ncbi.nlm.nih.gov/nucleotide/CP042977.1?report=genbank&log$=nuclalign&blast_rank=1&RID=ASUZ6XS601R) | 13 | 68% |
| Streptococcus pyogenes | [CP043530.1](https://www.ncbi.nlm.nih.gov/nucleotide/CP043530.1?report=genbank&log$=nuclalign&blast_rank=1&RID=ASV05CFR01R) | 12 | 63% |
| Streptococcus pneumoniae | [CP050175.1](https://www.ncbi.nlm.nih.gov/nucleotide/CP050175.1?report=genbank&log$=nuclalign&blast_rank=1&RID=ASUZFPEE01R) | 11 | 58% |
| Staphylococcus aureus | [AP020315.1](https://www.ncbi.nlm.nih.gov/nucleotide/AP020315.1?report=genbank&log$=nuclalign&blast_rank=1&RID=ASV2WNB801R) | 13 | 68% |
| Pseudomonas aeruginosa | [CP046602.1](https://www.ncbi.nlm.nih.gov/nucleotide/CP046602.1?report=genbank&log$=nuclalign&blast_rank=1&RID=ASV383BS01R) | 16 | 84% |
| Candida albicans | [CP025168.1](https://www.ncbi.nlm.nih.gov/nucleotide/CP025168.1?report=genbank&log$=nuclalign&blast_rank=1&RID=ASV3SXUN01R) | 14 | 74% |

**Table S8. RT-LAMP Backward Inner Primer homology check.**

The complementary sequence of the **Gene N-A-BIP primer** (bases in red) was checked against NCBI reference collection of the following microorganisms common in upper respiratory infections using BLASTn. The top hit for % homology with number of identical bases for each type of microorganism is displayed.

Gene N-A-BIP primer: **AGACGGCATCATATGGGTTGCA**CGGGTGCCAATGTGATCT

|  | Top hit ID | No. of identical  bases (/22) | % Homology |
| --- | --- | --- | --- |
| Influenza A | KY402002.1 | 14 | 64% |
| Influenza B | MH233895.1 | 12 | 55% |
| Human Cov NL63 | MN306040.1 | 10 | 45% |
| OC43 | LC506820.1 | 10 | 45% |
| HKU1 | MH940245.1 | 10 | 45% |
| 229E | MN369046.1 | 10 | 45% |
| MERS-CoV | MN723544.1 | 14 | 64% |
| SARS-CoV | AY502924.1 | 18 | 82% |
| Human parainfluenza virus 1 | KX570602.1 | 9 | 41% |
| Human parainfluenza virus 2 | AB176531.1 | 8 | 36% |
| Human parainfluenza virus 3 | MH678682.1 | 13 | 59% |
| Human parainfluenza virus 4a | MN369047.1 | 8 | 36% |
| Human parainfluenza virus 4b | MN306058.1 | 8 | 36% |
| Human adenovirus 1 | MH183293.1 | 9 | 41% |
| Human adenovirus E4 | MN513347.1 | 8 | 36% |
| Human adenovirus 4a | AB679756.1 | 8 | 36% |
| Human adenovirus 5 | KX868466.2 | 9 | 41% |
| Human adenovirus 7 | MK492257.1 | 9 | 41% |
| Haemophilus influenzae | CP044497.1 | 11 | 50% |
| Haemophilus parainfluenzae | FQ312002.1 | 14 | 64% |
| Human rhinovirus | HQ123440.1 | 12 | 55% |
| Legionella pneumophila | LR134176.1 | 14 | 64% |
| Mycobacterium tuberculosis | CP046308.1 | 12 | 55% |
| Mycoplasma pneumoniae | CP039761.1 | 11 | 50% |
| Klebsiella pneumoniae | CP050822.1 | 15 | 68% |
| Streptococcus pyogenes | CP043530.1 | 14 | 64% |
| Streptococcus pneumoniae | CP050175.1 | 12 | 55% |
| Staphylococcus aureus | CP050690.1 | 14 | 64% |
| Pseudomonas aeruginosa | LS483402.1 | 17 | 77% |
| Candida albicans | CP025168.1 | 15 | 68% |

**Table S9.** Result interpretation of 22 SARS-CoV-2 positive and 20 known negative nasopharyngeal samples using multiplex direct RT-PCR kit on Bio-Rad and Epidax®. Intensity thresholding of 15, 11 and 3 were used for E gene (FAM), N gene (HEX) and RNase P (Texas Red) respectively.

| **S/N** | **Sample No.** | **Bio-Rad** | | | | **Epidax®** | | | |
| --- | --- | --- | --- | --- | --- | --- | --- | --- | --- |
|  |  | **E gene (FAM)** | **N gene (HEX)** | **RNase P (TEX)** | **Conclusion** | **E gene (FAM)** | **N gene (HEX)** | **RNase P (TEX)** | **Conclusion** |
| 1 | POS2_POS3 | +/ + | +/ + | +/ + | +/ + | +/ + | +/ + | +/ + | +/ + |
| 2 | POS5_POS6 | +/ + | +/ + | +/ + | +/ + | +/ + | +/ + | +/ + | +/ + |
| 3 | POS7_POS8 | +/ + | +/ + | +/ + | +/ + | +/ + | +/ + | +/ + | +/ + |
| 4 | POS9_POS10 | +/ + | +/ + | +/ + | +/ + | +/ + | +/ + | +/ + | +/ + |
| 5 | POS11_POS12 | False -/ False - | +/ + | +/ + | +/ + | False -/ False - | False -/ + | +/ + | False -/ + |
| 6 | POS13_POS14 | False -/ + | +/ + | +/ + | +/ + | False -/ + | +/ + | +/ + | +/ + |
| 7 | POS17_POS18 | +/ + | +/ + | +/ + | +/ + | False -/ + | +/ + | +/ + | +/ + |
| 8 | POS19_POS20 | +/ + | +/ + | +/ + | +/ + | +/ - | +/ + | +/ + | +/ + |
| 9 | POS23_POS24 | +/ + | +/ + | +/ + | +/ + | +/ - | +/ + | +/ + | +/ + |
| 10 | POS25_POS26 | +/ + | +/ + | +/ + | +/ + | -/ - | +/ + | +/ + | +/ + |
| 11 | POS27_POS28 | +/ + | +/ + | +/ + | +/ + | +/ + | +/ + | +/ + | +/ + |
| 12 | POS29_POS30 | +/ + | +/ + | +/ + | +/ + | -/ - | +/ + | +/ + | +/ + |
| 13 | NEG3_NEG4 | -/ - | -/ - | +/ + | -/ - | -/ - | -/ - | +/ + | -/ - |
| 14 | NEG5_NEG6 | -/ - | -/ - | +/ + | -/ - | -/ - | -/ - | +/ + | -/ - |
| 15 | NEG7_NEG8 | False +/ - | -/ - | +/ + | Equivocal/- | -/ - | -/ - | +/ + | -/ - |
| 16 | NEG9_NEG10 | -/ - | -/ - | +/ + | -/ - | -/ - | -/ - | +/ + | -/ - |
| 17 | NEG11_NEG12 | -/ - | -/ - | +/ + | -/ - | -/ - | -/ - | +/ - | -/Invalid |
| 18 | NEG15_NEG16 | -/ - | -/ - | +/ + | -/ - | -/ - | -/ - | +/ + | -/ - |
| 19 | NEG17_NEG18 | -/ - | -/ - | +/ + | -/ - | -/ - | -/ - | +/ + | -/ - |
| 20 | NEG23_NEG24 | -/ False + | -/ False + | +/ + | -/ False + | -/ - | -/ - | +/ + | -/ - |
| 21 | NEG25_NEG26 | -/ - | -/ False + | +/ + | -/ False + | -/ - | -/ - | +/ + | -/ - |
| 22 | NEG27_NEG28 | -/ - | -/ - | +/ + | -/ - | -/ - | -/ - | +/ + | -/ - |

**Table S10.** Ct values of 22 SARS-CoV-2 positive and 20 known negative nasopharyngeal samples using direct RT-PCR kit (N gene assay by HEX channel) on Bio-Rad and Epidax®.

| **S/N** | **Sample** | **Bio-Rad** | | | |  | **Epidax®** | | | | |
| --- | --- | --- | --- | --- | --- | --- | --- | --- | --- | --- | --- |
|  |  | **(1) NC** | **(2) Sample 1** | **(3) Sample 2** | **(4) PC** | **Conclusion** | **(1) NC** | **(2) Sample 1** | **(3) Sample 2** | **(4) PC** | **Conclusion** |
| 1 | POS2_POS3 | NA | 16.12 | 23.67 | 32.91 | +/+ | NA | 18.5 | 24.6 | 28.8 | +/+ |
| 2 | POS5_POS6 | NA | 23.23 | 20.41 | 33.23 | +/+ | NA | 21.7 | 20.3 | 34.2 | +/+ |
| 3 | POS7_POS8 | NA | 19.32 | 19.7 | 32.95 | +/+ | NA | 20 | 20.8 | 33.4 | +/+ |
| 4 | POS9_POS10 | NA | 19.25 | 20.81 | 32 | +/+ | NA | 21 | 21.7 | 26.2 | +/+ |
| 5 | POS11_POS12 | NA | 37.92 | 38.04 | 34.21 | +/+ | NA | NA | 37.5 | 32.6 | False -/+ |
| 6 | POS13_POS14 | NA | 34.67 | 33.34 | 33.7 | +/+ | NA | 36.9 | 34.6 | 33.7 | +/+ |
| 7 | POS17_POS18 | NA | 18.07 | 18.9 | 32.06 | +/+ | NA | 17.5 | 17.8 | 35.9 | +/+ |
| 8 | POS19_POS20 | NA | 15.6 | 37.26 | 32.15 | +/+ | NA | 15.9 | 36.8 | 33.7 | +/+ |
| 9 | POS23_POS24 | NA | 33.91 | 33.61 | 32.21 | +/+ | NA | 34.92 | 35.4 | 32.96 | +/+ |
| 10 | POS25_POS26 | 37.85 | 31.11 | 37.16 | 31.69 | +/+ | NA | 31.33 | 35.93 | 32.14 | +/+ |
| 11 | POS27_POS28 | NA | 35.03 | 32.53 | 32.33 | +/+ | 39.01 | 33.62 | 32.5 | 33.03 | +/+ |
| 12 | POS29_POS30 | NA | 31.44 | 32.83 | 31.71 | +/+ | NA | 33.34 | 33.55 | 32.66 | +/+ |
| 13 | NEG3_NEG4 | NA | NA | NA | 32.1 | -/- | NA | NA | NA | 33 | -/- |
| 14 | NEG5_NEG6 | NA | NA | NA | 34.01 | -/- | NA | NA | 44.5 | 33.6 | -/- |
| 15 | NEG7_NEG8 | NA | NA | NA | 33.26 | -/- | NA | NA | NA | 33.4 | -/- |
| 16 | NEG9_NEG10 | NA | NA | NA | 32.44 | -/- | NA | NA | NA | 34.1 | -/- |
| 17 | NEG11_NEG12 | NA | NA | NA | 34.03 | -/- | NA | NA | NA | 31.7 | -/- |
| 18 | NEG15_NEG16 | NA | NA | NA | 33.69 | -/- | NA | NA | NA | 34.4 | -/- |
| 19 | NEG17_NEG18 | NA | NA | NA | 29.27 | -/- | NA | NA | NA | 30.7 | -/- |
| 20 | NEG23_NEG24 | NA | 36.89 | NA | 31.21 | False +/- | NA | NA | NA | 30.86 | -/- |
| 21 | NEG25_NEG26 | NA | NA | 35.55 | 30.99 | -/ False + | NA | NA | NA | 33.35 | -/- |
| 22 | NEG27_NEG28 | NA | NA | NA | 31.25 | -/- | NA | NA | NA | 31.43 | -/- |

**Table S11**. Comparison of POC devices for nucleic acid detection of SARS-CoV-2

* Nasopharyngeal swab (NPS). Universal Transport Medium (UTM)

| **S/N** | **Platform** | **Sample type** | **Sample preparation** | **Analytical method** | **Reaction volume** | **Limit of detection (copies per μL)** | **Clinical performance** | **Maximum samples per run** | **Detection time (min)** | **Target region of SARS-CoV-2** |
| --- | --- | --- | --- | --- | --- | --- | --- | --- | --- | --- |
| 1 | Our system (Epidax®) | 2.5 μL of NPS sample or extracted nucleic acid | Not required for direct RT-PCR, or manual nucleic acid extraction for RT-LAMP and RT-PCR | Real-time RT-PCR.  End-point RT-PCR.  RT-LAMP | 10 - 12.5 μL | Real-time RT-PCR: 80% detection at 1 copy per μL;  100% detection at 10 copies per μL | - Real-time RT-PCR: Sensitivity: 100% Specificity: 100% - Real-time direct RT-PCR: Sensitivity: 96% Specificity: 100% | 4 (RT-PCR) or 6 (RT-LAMP) | 60 min (sample-to-result direct RT-PCR using NPS sample). 30 min (RT-LAMP using nucleic acid template) | N1, N, E, N-A |
| 2 | Li et al^2^ | Original swab in 800 μL of lysis buffer | Magnetic bead-based nucleic acid extraction | Real-time  RT-LAMP | 160 μL (80 μL eluted nucleic acid, 80 μL RT-LAMP mastermix) | 2.5 | - | 1 sample (up to 8 target detection) | 80 min (RT-LAMP at 65 °C, 40 min) | N, ORF1ab |
| 3 | Tian et al^3^ | 4 μL of sample | Chemical lysis | End-point  RT-LAMP | 21 μL (4 μL of sample and 17 μL of RT-LAMP master mix) | 0.5 | - | 1 | 70 min sample-to-answer (RT-LAMP at 65 °C, 60 min) | N |
| 4 | Soares et al^4^ | 1 μL of sample | Heat inactivation | End-point  RT-LAMP | 10 μL (1 μL of sample) | 10 | Sensitivity: 96.6% for samples with Ct<26. Specificity: 100% | 1 | 60 min (RT-LAMP at 65 °C, 30 min) | As1e |
| 5 | Xiong et al^5^ | 12 μL of nucleic acid extracted from throat swab (added to 60 μL of LAMP buffer) | Manual nucleic acid extraction | Real-time LAMP | 5 μL | 10 | Sensitivity: 100% Specificity: 100% | 8 | 40 min (RT was performed manually) | N, O |
| 6 | Xing et al^6^ | 400 μL of oropharyngeal sample | Silica membrane-based extraction | RT-RPA and LAMP | - | 0.15 | Sensitivity: 99.71% Specificity: 98.46% | 5 | 45 (RNA extraction 10 min, RT-RPA 15 min, LAMP 20 min) | ORF1ab, N |
| 7 | Davidson et al^7^ | 25 μL of diluted saliva (5% v/v in water) | Without preprocessing | Colorimetric  RT-LAMP | 25 μL | 200 | Analytical sensitivity: 76% (using naked eyes), 97% (using image analysis). Specificity: 100% | 1 | 60 min | ORF1ab |
| 8 | Garneret et al^8^ | 100 μL of sample | Membrane based extraction | Real-time  RT-LAMP | - | 1 | Specificity: 100% | 2 | 80 min | ORF1ab |
| 9 | Xun et al (SPOT system)^9^ | Saliva | Heat inactivation | RT-LAMP and detection by PfAgo-based target sequence detection | 40 μL (5 μL of *in vitro* transcribed RNA, or pretreated sample) | 0.44 (N gene) 1.09 (E gene) | Sensitivity: 93.3%  Specificity: 100% | 1 | 30 min | N, E |
| 10 | Nguyen et al^10^ | 2μL of NPS sample in UTM | Thermal lysis with vibration | Real-time  RT-LAMP | 10 μL | 20 | - | 4 | 60 min | As1e, N, E |
| 11 | Huang et al (Onestart system)^11^ | 800 μL of pharyngeal swab sample | Solid-phase extraction | Real-time RT-PCR | 3.5 μL | 1 | Specificity: 100% | 32 | 90 min | ORF1ab |
| **Commercial devices for SARS-CoV-2 detection** | | | | | | | | | | |
| 12 | Cepheid® (Xpert® Xpress SARS-CoV-2)^12^ | 300 μL of NPS sample | Lysis by sonication | Real-time RT-PCR | - | 0.1 | Sensitivity: 98.3%  Specificity: 100% | 4 per instrument | 46 min | N2, E |
| 13 | GenMark Diagnostics (ePlex system)^12^ | 200 μL of NPS sample | Magnetic solid phase extraction | RT-PCR and eSensor detection | - | 1 | Sensitivity: 91.4% Specificity: 100% | 6 per tower (1 to 4 towers) | 90 min | N |
| 14 | Abbott (ID Now COVID-19)^12^ | 200 μL of NPS sample | Sample is mixed with elution/lysis buffer manually | Isothermal amplification | - | 20 | Sensitivity: 87.7% Specificity: 100% | 1 per instrument | 20 - 30 min | RdRp |
| 15 | DnaNudge (COVIDNudge test)^13^ | Original NPS | Lysis buffer and silica frit filter | Real-time RT-PCR | 1.8 μL | 5 (N3 gene) 10 (N1, N2 & E genes) 50 (rdrp1, rdrp2) | Sensitivity: 94% Specificity: 100% | 72 wells for various target genes | 90 min | rdrp1, rdrp2, E, N, N1, N2 and N3 |
| 16 | Meridian Bioscience (Revogene® SARS-CoV-2)^14^ | NPS | The cartridge performs viral lysis | Real-time RT-PCR | - | 4.575 | Sensitivity: 97.7% Specificity: 97.7% | 8 | 90 min sample to answer | N |
| 17 | BioFire Diagnostics (BioFire Respiratory Panel 2.1-EZ)^15^ | 300 μL of NPS sample | Chemical lysis and magnetic bead-based nucleic acid extraction | End-point multiplex PCR | - | 0.3 | Sensitivity: 98.4% Specificity: 98.9% | 1 | 60 min sample to result | 19 targets (15 viral and 4 bacterial targets, including SARS-CoV-2) |
| 18 | QuantuMDx (Q-POC SARS-CoV-2)^16^ | Mid nasal swab | No sample preprocessing required | Real-time multiplex PCR | - | 1 | Sensitivity: 96.9% (at Ct cut-off 35), 80% (at Ct cut-off 40) Specificity: 98.3% | 1 | 32 min | ORF1, N, S |
| 19 | Mesa Biotech Inc. (Accula SARS-CoV-2 Test)^17^ | 10 μL of NPS sample in UTM or saline | Chemical lysis | RT-PCR, detection by lateral flow | - | 0.15 | Sensitivity: 68% Specificity: 100% | 1 | 30 min sample to answer | N |
| 20 | Roche (cobas® Liat SARS-CoV-2)^18^ | 200 μL of NPS sample in transport media | Magnetic bead-based nucleic acid extraction | Real-time RT-PCR | - | 0.012 TCID_50_/ml | Sensitivity: 100% Specificity: 97.4% | 1 | 20 min | ORF1ab, N |
| 21 | Luminex (ARIES® SARS-CoV-2 Assay)^19^ | 200 μL of NPS sample in UTM | Magnetic bead-based nucleic acid extraction | Real-time RT-PCR | - | 1000 copies per reaction | Sensitivity: 100% Specificity: 100% | 6 | 120 min | ORF, N |
| 22 | DiaSorin Molecular (Simplexa® COVID-19 Direct Kit)^20^ | 50 μL of NPS sample in UTM | Without preprocessing | Real-time RT-PCR | - | 0.5 | Sensitivity: 100% Specificity: 100% | 8 | 90 min | ORF1ab, S |

# References

1 Butler, D. J. *et al.* Host, Viral, and Environmental Transcriptome Profiles of the Severe Acute Respiratory Syndrome Coronavirus 2 (SARS-CoV-2). *bioRxiv*, 2020.2004.2020.048066, doi:10.1101/2020.04.20.048066 (2020).

2 Li, N. *et al.* Multiplexed detection of respiratory pathogens with a portable analyzer in a "raw-sample-in and answer-out" manner. *Microsyst Nanoeng* **7**, 94, doi:10.1038/s41378-021-00321-7 (2021).

3 Tian, F. *et al.* A fully automated centrifugal microfluidic system for sample-to-answer viral nucleic acid testing. *Science China Chemistry* **63**, 1498-1506, doi:10.1007/s11426-020-9800-6 (2020).

4 Soares, R. R. G. *et al.* Sample-to-answer COVID-19 nucleic acid testing using a low-cost centrifugal microfluidic platform with bead-based signal enhancement and smartphone read-out. *Lab Chip* **21**, 2932-2944, doi:10.1039/d1lc00266j (2021).

5 Xiong, H. *et al.* Rapid Differential Diagnosis of Seven Human Respiratory Coronaviruses Based on Centrifugal Microfluidic Nucleic Acid Assay. *Analytical Chemistry* **92**, 14297-14302, doi:10.1021/acs.analchem.0c03364 (2020).

6 Xing, W. *et al.* A Highly Automated Mobile Laboratory for On-site Molecular Diagnostics in the COVID-19 Pandemic. *Clin Chem* **67**, 672-683, doi:10.1093/clinchem/hvab027 (2021).

7 Davidson, J. L. *et al.* A paper-based colorimetric molecular test for SARS-CoV-2 in saliva. *Biosensors and Bioelectronics: X* **9**, 100076, doi:<https://doi.org/10.1016/j.biosx.2021.100076> (2021).

8 Garneret, P. *et al.* Performing point-of-care molecular testing for SARS-CoV-2 with RNA extraction and isothermal amplification. *PLoS One* **16**, e0243712, doi:10.1371/journal.pone.0243712 (2021).

9 Xun, G., Lane, S. T., Petrov, V. A., Pepa, B. E. & Zhao, H. A rapid, accurate, scalable, and portable testing system for COVID-19 diagnosis. *Nature Communications* **12**, 2905, doi:10.1038/s41467-021-23185-x (2021).

10 Nguyen, H. Q., Bui, H. K., Phan, V. M. & Seo, T. S. An internet of things-based point-of-care device for direct reverse-transcription-loop mediated isothermal amplification to identify SARS-CoV-2. *Biosensors and Bioelectronics* **195**, 113655, doi:<https://doi.org/10.1016/j.bios.2021.113655> (2022).

11 Huang, E. *et al.* A fully automated microfluidic PCR-array system for rapid detection of multiple respiratory tract infection pathogens. *Anal Bioanal Chem* **413**, 1787-1798, doi:10.1007/s00216-021-03171-4 (2021).

12 Zhen, W., Smith, E., Manji, R., Schron, D. & Berry, G. J. Clinical Evaluation of Three Sample-to-Answer Platforms for Detection of SARS-CoV-2. *Journal of Clinical Microbiology* **58**, e00783-00720, doi:10.1128/JCM.00783-20 (2020).

13 Gibani, M. M. *et al.* Assessing a novel, lab-free, point-of-care test for SARS-CoV-2 (CovidNudge): a diagnostic accuracy study. *The Lancet Microbe* **1**, e300-e307, doi:10.1016/S2666-5247(20)30121-X (2020).

14 Bioscience, M. Revogene® SARS-CoV-2 Instruction for Use. <https://www.fda.gov/media/154060/download>. Accessed 11 Apr 2022. (2021).

15 Diagnostics, B. BioFire Respiratory Panel 2.1-EZ. <https://docs.biofiredx.com/wp-content/uploads/BFR0001-0044-BioFire-RP2.1-EZ-Instructions-for-Use-EUA-1.pdf>. Accessed 11 Apr 2022. (2020).

16 QuantuMDx. Q-POC SARS-CoV-2. <https://www.quantumdx.com/products/q-poc/>. Accessed 11 Apr 2022. (2022).

17 Hogan, C. A. *et al.* Comparison of the Accula SARS-CoV-2 Test with a Laboratory-Developed Assay for Detection of SARS-CoV-2 RNA in Clinical Nasopharyngeal Specimens. *J Clin Microbiol* **58**, doi:10.1128/jcm.01072-20 (2020).

18 Hansen, G. *et al.* Clinical Performance of the Point-of-Care cobas Liat for Detection of SARS-CoV-2 in 20 Minutes: a Multicenter Study. *J Clin Microbiol* **59**, doi:10.1128/jcm.02811-20 (2021).

19 Tanida, K. *et al.* Evaluation of the automated cartridge-based ARIES SARS-CoV-2 Assay (RUO) against automated Cepheid Xpert Xpress SARS-CoV-2 PCR as gold standard. *Eur J Microbiol Immunol (Bp)* **10**, 156-164, doi:10.1556/1886.2020.00017 (2020).

20 Bordi, L. *et al.* Rapid and sensitive detection of SARS-CoV-2 RNA using the Simplexa™ COVID-19 direct assay. *J Clin Virol* **128**, 104416, doi:10.1016/j.jcv.2020.104416 (2020).
