# Supplementary material for: Modular micro-PCR system for the onsite rapid diagnosis of COVID-19
Source: Microsyst Nanoeng. 2022 Jul 19;8:82. doi: 10.1038/s41378-022-00400-3 (PMC9294789; doi:10.1038/s41378-022-00400-3)
Supplement: Supplementary file 5 — IFU_CDC 2019-nCoV diagnostic rt-PCR [file 41378_2022_400_MOESM5_ESM.pdf]

CDC 2019-Novel Coronavirus (2019-nCoV) Real-Time RT-PCR Diagnostic Panel

For Emergency Use Only

Instructions for Use

Catalog # 2019-nCoVEUA-01
1000 reactions

For *In-vitro* Diagnostic (IVD) Use

Rx Only

Centers for Disease Control and Prevention
Division of Viral Diseases
1600 Clifton Rd NE
Atlanta GA 30329

Table of Contents

Intended Use 2

Summary and Explanation 2

Principles of the Procedure..3

Materials Required (Provided) 5

Materials Required (But Not Provided)..6

Warnings and Precautions..9

Reagent Storage, Handling, and Stability 10

Specimen Collection, Handling, and Storage 11

Specimen Referral to CDC 12

Reagent and Controls Preparation 12

General Preparation...13

Nucleic Acid extraction 13

Assay Set Up..15

Create a Run Template on the Applied Biosystems 7500 Fast Dx Real-time PCR Instrument (Required if no template exists)..19

Defining the Instrument Settings..25

Running a Test...28

Interpretation of Results and Reporting...33

2019-nCoV rRT-PCR Diagnostic Panel Results Interpretation Guide.............................35

Quality Control...36

Limitations..36

Conditions of Authorization for the Laboratory..37

Performance Characteristics 38

Disposal..45

References 45

Contact Information, Ordering, and Product Support 46

Intended Use

The CDC 2019-Novel Coronavirus (2019-nCoV) Real-Time RT-PCR Diagnostic Panel is a real-time RT-PCR test intended for the qualitative detection of nucleic acid from the 2019-nCoV in upper and lower respiratory specimens (such as nasopharyngeal or oropharyngeal swabs, sputum, lower respiratory tract aspirates, bronchoalveolar lavage, and nasopharyngeal wash/aspirate or nasal aspirate) collected from individuals who meet 2019-nCoV clinical and/or epidemiological criteria (for example, clinical signs and symptoms associated with 2019-nCoV infection, contact with a probable or confirmed 2019-nCoV case, history of travel to geographic locations where 2019-nCoV cases were detected, or other epidemiologic links for which 2019-nCoV testing may be indicated as part of a public health investigation). Testing in the United States is limited to laboratories certified under the Clinical Laboratory Improvement Amendments of 1988 (CLIA), 42 U.S.C. § 263a, to perform high complexity tests.

Results are for the identification of 2019-nCoV RNA. The 2019-nCoV RNA is generally detectable in upper and lower respiratory specimens during infection. Positive results are indicative of active infection with 2019-nCoV but do not rule out bacterial infection or co-infection with other viruses. The agent detected may not be the definite cause of disease. Laboratories within the United States and its territories are required to report all positive results to the appropriate public health authorities.

Negative results do not preclude 2019-nCoV infection and should not be used as the sole basis for treatment or other patient management decisions. Negative results must be combined with clinical observations, patient history, and epidemiological information.

Testing with the CDC 2019-nCoV Real-Time RT-PCR Diagnostic Panel is intended for use by trained laboratory personnel who are proficient in performing real-time RT-PCR assays. The CDC 2019-Novel Coronavirus (2019-nCoV) Real-Time RT-PCR Diagnostic Panel is only for use under a Food and Drug Administration's Emergency Use Authorization.

Summary and Explanation

An outbreak of pneumonia of unknown etiology in Wuhan City, Hubei Province, China was initially reported to WHO on December 31, 2019. Chinese authorities identified a novel coronavirus (2019-nCoV), which has resulted in thousands of confirmed human infections in multiple provinces throughout China and many countries including the United States. Cases of asymptomatic infection, mild illness, severe illness, and some deaths have been reported.

The CDC 2019-nCoV Real-Time RT-PCR Diagnostic Panel is a molecular *in vitro* diagnostic test that aids in the detection and diagnosis 2019-nCoV and is based on widely used nucleic acid amplification technology. The product contains oligonucleotide primers and dual-labeled hydrolysis probes (TaqMan®) and control material used in rRT-PCR for the *in vitro* qualitative detection of 2019-nCoV RNA in respiratory specimens.

The term "qualified laboratories" refers to laboratories in which all users, analysts, and any person reporting results from use of this device should be trained to perform and interpret the results from this procedure by a competent instructor prior to use.

Principles of the Procedure

The oligonucleotide primers and probes for detection of 2019-nCoV were selected from regions of the virus nucleocapsid (N) gene. The panel is designed for specific detection of the 2019-nCoV (two primer/probe sets). An additional primer/probe set to detect the human RNase P gene (RP) in control samples and clinical specimens is also included in the panel.

RNA isolated and purified from upper and lower respiratory specimens is reverse transcribed to cDNA and subsequently amplified in the Applied Biosystems 7500 Fast Dx Real-Time PCR Instrument with SDS version 1.4 software. In the process, the probe anneals to a specific target sequence located between the forward and reverse primers. During the extension phase of the PCR cycle, the 5' nuclease activity of Taq polymerase degrades the probe, causing the reporter dye to separate from the quencher dye, generating a fluorescent signal. With each cycle, additional reporter dye molecules are cleaved from their respective probes, increasing the fluorescence intensity. Fluorescence intensity is monitored at each PCR cycle by Applied Biosystems 7500 Fast Dx Real-Time PCR System with SDS version 1.4 software.

Detection of viral RNA not only aids in the diagnosis of illness but also provides epidemiological and surveillance information.

Summary of Preparation and Testing Process

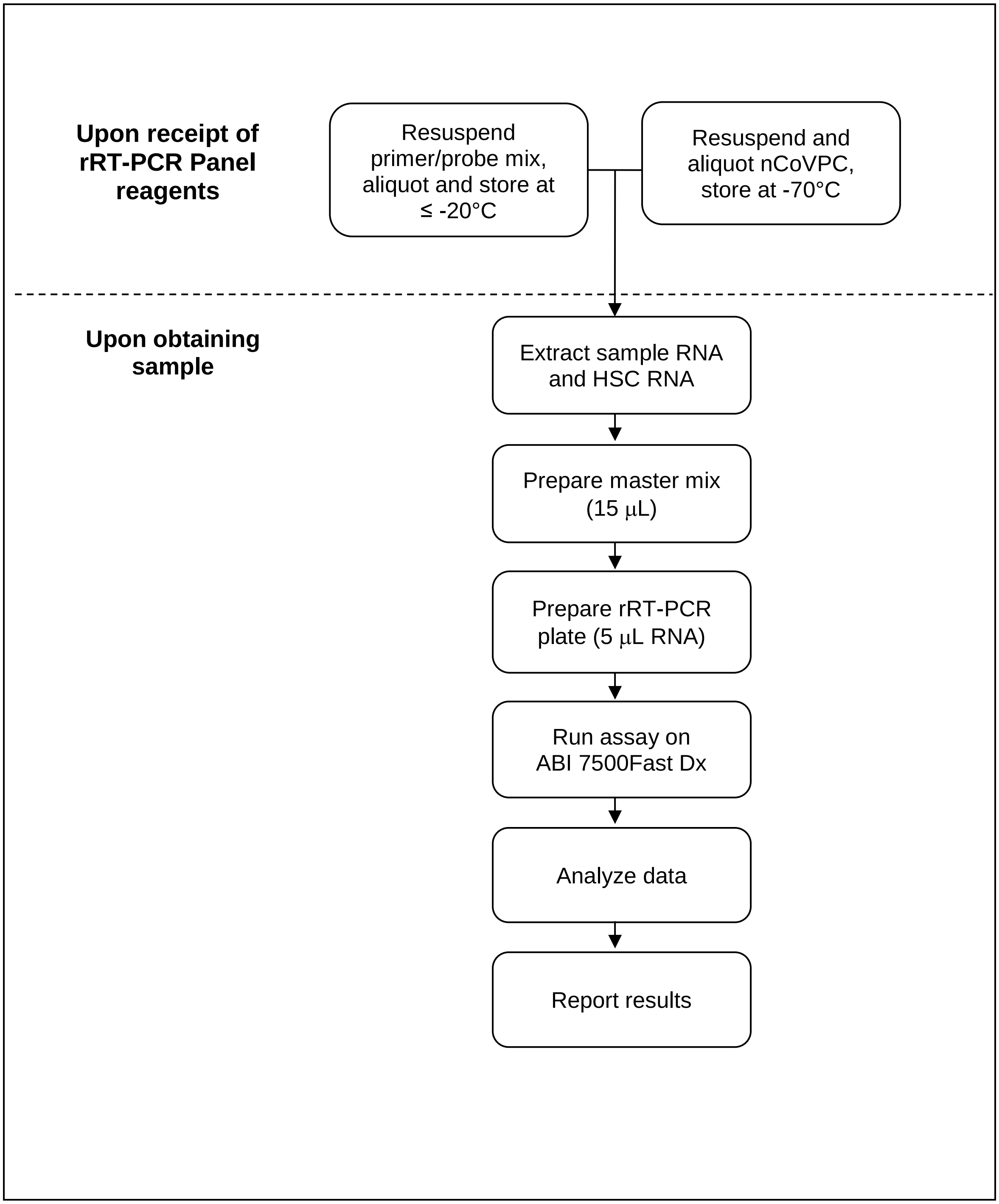

Materials Required (Provided)

Note: CDC will maintain on its website a list of commercially available lots of primer and probe sets and/or positive control materials that are acceptable alternatives to the CDC primer and probe set and/or positive control included in the Diagnostic Panel. Only material distributed through the CDC International Reagent Resource and specific lots of material posted to the CDC website are acceptable for use with this assay under CDC's Emergency Use Authorization.

This list of acceptable alternative lots of primer and probe materials and/or positive control materials will be available at:
https://www.cdc.gov/coronavirus/2019-nCoV/lab/index.html

Primers and Probes:

Catalog #2019-nCoVEUA-01 Diagnostic Panel Box #1:

Reagent Label	*Part #*	*Description*	*Quantity / Tube*	*Reactions / Tube*
2019-nCoV_N1	RV202001<br>RV202015	2019-nCoV_N1 Combined Primer/Probe Mix	22.5 nmol	1000
2019-nCoV_N2	RV202002<br>RV202016	2019-nCoV_N2 Combined Primer/Probe Mix	22.5 nmol	1000
RP	RV202004<br>RV202018	Human RNase P Forward Primer/Probe Mix	22.5 nmol	1000

Positive Control (either of the following products are acceptable)

Catalog #2019-nCoVEUA-01 Diagnostic Panel Box #2:

Reagent Label	*Part #*	*Description*	*Quantity*	*Notes*
nCoVPC	RV202005	2019-nCoV Positive Control (nCoVPC)<br>For use as a positive control with the CDC 2019-nCoV Real-Time RT-PCR Diagnostic Panel procedure. The nCoVPC contains noninfectious positive control material supplied in a dried state and must be resuspended before use. nCoVPC consists of *in vitro* transcribed RNA. nCoVPC will yield a positive result with each assay in the 2019-nCoV Real-Time RT-PCR Diagnostic Panel including RP.	4 tubes	Provides (800) 5 µL test reactions

Catalog #VTC-04 CDC 2019-nCoV Positive Control (nCoVPC)

Reagent Label	*Part #*	*Description*	*Quantity*	*Notes*
nCoVPC	RV202005	2019-nCoV Positive Control (nCoVPC)<br>For use as a positive control with the CDC 2019-nCoV Real-Time RT-PCR Diagnostic Panel procedure. The nCoVPC contains noninfectious positive control material supplied in a dried state and must be resuspended before use. nCoVPC consists of *in vitro* transcribed RNA. nCoVPC will yield a positive result with each assay in the 2019-nCoV Real-Time RT-PCR Diagnostic Panel including RP.	4 tubes	Provides (800) 5 µL test reactions

Materials Required (But Not Provided)

Human Specimen Control (HSC)

Description	Quantity	CDC Catalog No.
Manufactured by CDC. For use as an RNA extraction procedural control to demonstrate successful recovery of RNA as well as extraction reagent integrity. The HSC consists of noninfectious (beta-Propiolactone treated) cultured human cell material supplied as a liquid suspended in 0.01 M PBS at pH 7.2-7.4.	10 vials x 500uL	KT0189

Acceptable alternatives to HSC:

- Negative human specimen material: Laboratories may prepare a volume of human specimen material (e.g., human sera or pooled leftover negative respiratory specimens) to extract and run alongside clinical samples as an extraction control. This material should be prepared in sufficient volume to be used across multiple runs. Material should be tested prior to use as the extraction control to ensure it generates the expected results for the HSC listed in these instructions for use.
- Contrived human specimen material: Laboratories may prepare contrived human specimen materials by suspending any human cell line (e.g., A549, Hela or 293) in PBS. This material should be prepared in sufficient volume to be used across multiple runs. Material should be tested prior to use as the extraction control to ensure it generates the expected results for the HSC listed in these instructions for use.

CDC will maintain on its website a list of commercially alternative extraction controls, if applicable, that are acceptable for use with this assay under CDC's Emergency Use Authorization, at: https://www.cdc.gov/coronavirus/2019-nCoV/lab/index.html

rRT-PCR Enzyme Mastermix Options

Reagent	Quantity	Catalog No.
Quantabio qScript XLT One-Step RT-qPCR ToughMix	100 x 20 µL rxns (1 x 1 mL)	95132-100
	2000 x 20 µL rxns (1 x 20 mL)	95132-02K
	500 x 20 µL rxns (5 x 1 mL)	95132-500
Quantabio UltraPlex 1-Step ToughMix (4X)	100 x 20 µL rxns (500 µL)	95166-100
	500 x 20 µL rxns (5 x 500 µL)	95166-500
	1000 x 20 µL rxns (1 x 5 mL)	95166-01K
Promega GoTaq® Probe 1- Step RT-qPCR System	200 x 20 µL rxns (2 mL)	A6120
	1250 x 20 µL rxns 12.5 mL	A6121
Thermofisher TaqPath™ 1-Step RT-qPCR Master Mix, CG	1000 reactions	A15299
	2000 reactions	A15300

RNA Extraction Options

For each of the kits listed below, CDC has confirmed that the external lysis buffer is effective for inactivation of SARS-CoV-2.

Instrument/Manufacturer	Extraction Kit	Catalog No.
QIAGEN	[2]QIAmp DSP Viral RNA Mini Kit	50 extractions (61904)
	[2]QIAamp Viral RNA Mini Kit	50 extractions (52904)<br>250 extractions (52906)
QIAGEN EZ1 Advanced XL	[2]EZ1 DSP Virus Kit	48 extractions (62724)<br>Buffer AVL (19073)<br>EZ1 Advanced XL DSP Virus Card (9018703)
	[2]EZ1 Virus Mini Kit v2.0	48 extractions (955134)<br>Buffer AVL (19073)<br>EZ1 Advanced XL Virus Card v2.0 (9018708)
[1]Roche MagNA Pure LC	[2]Total Nucleic Acid Kit	192 extractions (03 038 505 001)
[1]Roche MagNA Pure Compact	[2]Nucleic Acid Isolation Kit I	32 extractions (03 730 964 001)
[1]Roche MagNA Pure 96	[2]DNA and Viral NA Small Volume Kit	576 extractions (06 543 588 001)<br>External Lysis Buffer (06 374 913 001)
[1]QIAGEN QIAcube	[2]QIAmp DSP Viral RNA Mini Kit	50 extractions (61904)
	[2]QIAamp Viral RNA Mini Kit	50 extractions (52904)<br>250 extractions (52906)
[1, 3]bioMérieux NucliSENS® easyMAG®<br>and<br>[1, 3]bioMérieux EMAG®<br>(Automated magnetic extraction reagents sold separately. Both instruments use the same reagents and disposables, with the exception of tips.)		EasyMAG® Magnetic Silica (280133)<br>EasyMAG® Lysis Buffer (280134)<br>EasyMAG® Lysis Buffer, 2 mL (200292)<br>EasyMAG® Wash Buffers 1,2, and 3<br>(280130, 280131, 280132)<br>EasyMAG® Disposables (280135)<br>Biohit Pipette Tips (easyMAG® only)<br>(280146)<br>EMAG®1000µL Tips (418922)

[1]Equivalence and performance of these extraction platforms for extraction of viral RNA were demonstrated with the CDC Human Influenza Virus Real-Time RT-PCR Diagnostic Panel (K190302). Performance characteristics of these extraction platforms with 2019-nCoV (SARS CoV-2) have not been demonstrated.

[2] CDC has confirmed that the external lysis buffer used with this extraction method is effective for inactivation of SARS-CoV-2.

[3] CDC has compared the concentration of inactivating agent in the lysis buffer used with this extraction method and has determined the concentration to be within the range of concentrations found effective in inactivation of SARS-CoV-2.

Equipment and Consumables Required (But Not Provided)

- Vortex mixer
- Microcentrifuge
- Micropipettes (2 or 10 µL, 200 µL and 1000 µL)
- Multichannel micropipettes (5-50 µl)
- Racks for 1.5 mL microcentrifuge tubes
- 2 x 96-well -20°C cold blocks
- 7500 Fast Dx Real-Time PCR Systems with SDS 1.4 software (Applied Biosystems; catalog #4406985 or #4406984)
- Extraction systems (instruments): QIAGEN EZ1 Advanced XL
- Molecular grade water, nuclease-free
- 10% bleach (1:10 dilution of commercial 5.25-6.0% hypochlorite bleach)
- DNA*Zap*TM (Ambion, cat. #AM9890) or equivalent
- RNAse AwayTM (Fisher Scientific; cat. #21-236-21) or equivalent
- Disposable powder-free gloves and surgical gowns
- Aerosol barrier pipette tips
- 1.5 mL microcentrifuge tubes (DNase/RNase free)
- 0.2 mL PCR reaction plates (Applied Biosystems; catalog #4346906 or #4366932)
- MicroAmp Optical 8-cap Strips (Applied Biosystems; catalog #4323032)

Warnings and Precautions

- For *in vitro* diagnostic use (IVD).
- For emergency use only.
- Follow standard precautions. All patient specimens and positive controls should be considered potentially infectious and handled accordingly.
- Do not eat, drink, smoke, apply cosmetics or handle contact lenses in areas where reagents and human specimens are handled.
- Handle all specimens as if infectious using safe laboratory procedures. Refer to Interim Laboratory Biosafety Guidelines for Handling and Processing Specimens Associated with 2019-nCoV https://www.cdc.gov/coronavirus/2019-nCoV/lab-biosafety-guidelines.html.
- Specimen processing should be performed in accordance with national biological safety regulations.
- If infection with 2019-nCoV is suspected based on current clinical and epidemiological screening criteria recommended by public health authorities, specimens should be collected with appropriate infection control precautions.
- Performance characteristics have been determined with human upper respiratory specimens and lower respiratory tract specimens from human patients with signs and symptoms of respiratory infection.
- Perform all manipulations of live virus samples within a Class II (or higher) biological safety cabinet (BSC).
- Use personal protective equipment such as (but not limited to) gloves, eye protection, and lab coats when handling kit reagents while performing this assay and handling materials including samples, reagents, pipettes, and other equipment and reagents.

- Amplification technologies such as PCR are sensitive to accidental introduction of PCR product from previous amplifications reactions. Incorrect results could occur if either the clinical specimen or the real-time reagents used in the amplification step become contaminated by accidental introduction of amplification product (amplicon). Workflow in the laboratory should proceed in a unidirectional manner.
  - Maintain separate areas for assay setup and handling of nucleic acids.
  - Always check the expiration date prior to use. Do not use expired reagent. Do not substitute or mix reagent from different kit lots or from other manufacturers.
  - Change aerosol barrier pipette tips between all manual liquid transfers.
  - During preparation of samples, compliance with good laboratory techniques is essential to minimize the risk of cross-contamination between samples, and the inadvertent introduction of nucleases into samples during and after the extraction procedure. Proper aseptic technique should always be used when working with nucleic acids.
  - Maintain separate, dedicated equipment (e.g., pipettes, microcentrifuges) and supplies (e.g., microcentrifuge tubes, pipette tips) for assay setup and handling of extracted nucleic acids.
  - Wear a clean lab coat and powder-free disposable gloves (not previously worn) when setting up assays.
  - Change gloves between samples and whenever contamination is suspected.
  - Keep reagent and reaction tubes capped or covered as much as possible.
  - Primers, probes (including aliquots), and enzyme master mix must be thawed and maintained on cold block at all times during preparation and use.
  - Work surfaces, pipettes, and centrifuges should be cleaned and decontaminated with cleaning products such as 10% bleach, "DNA*Zap*™" or "RNase AWAY®" to minimize risk of nucleic acid contamination. Residual bleach should be removed using 70% ethanol.
- RNA should be maintained on cold block or on ice during preparation and use to ensure stability.
- Dispose of unused kit reagents and human specimens according to local, state, and federal regulations.

Reagent Storage, Handling, and Stability

- Store all dried primers and probes and the positive control, nCoVPC, at 2-8°C until re-hydrated for use. Store liquid HSC control materials at ≤ -20°C.
  Note: Storage information is for CDC primer and probe materials obtained through the International Reagent Resource. If using commercial primers and probes, please refer to the manufacturer's instructions for storage and handling.
- Always check the expiration date prior to use. Do not use expired reagents.
- Protect fluorogenic probes from light.
- Primers, probes (including aliquots), and enzyme master mix must be thawed and kept on a cold block at all times during preparation and use.
- Do not refreeze probes.
  Controls and aliquots of controls must be thawed and kept on ice at all times during preparation and use.

Specimen Collection, Handling, and Storage

Inadequate or inappropriate specimen collection, storage, and transport are likely to yield false test results. Training in specimen collection is highly recommended due to the importance of specimen quality. CLSI MM13-A may be referenced as an appropriate resource.

- Collecting the Specimen
  - Refer to Interim Guidelines for Collecting, Handling, and Testing Clinical Specimens from Patients Under Investigation (PUIs) for 2019 Novel Coronavirus (2019-nCoV) https://www.cdc.gov/coronavirus/2019-nCoV/guidelines-clinical-specimens.html
  - Follow specimen collection device manufacturer instructions for proper collection methods.
  - Swab specimens should be collected using only swabs with a synthetic tip, such as nylon or Dacron®, and an aluminum or plastic shaft. Calcium alginate swabs are unacceptable and cotton swabs with wooden shafts are not recommended. Place swabs immediately into sterile tubes containing 1-3 ml of viral transport media.
- Transporting Specimens
  - Specimens must be packaged, shipped, and transported according to the current edition of the International Air Transport Association (IATA) Dangerous Goods Regulation. Follow shipping regulations for UN 3373 Biological Substance, Category B when sending potential 2019-nCoV specimens. Store specimens at 2-8°C and ship overnight to CDC on ice pack. If a specimen is frozen at -70°C or lower, ship overnight to CDC on dry ice.
- Storing Specimens
  - Specimens can be stored at 2-8°C for up to 72 hours after collection.
  - If a delay in extraction is expected, store specimens at -70°C or lower.
  - Extracted nucleic acid should be stored at -70°C or lower.

Specimen Referral to CDC

For state and local public health laboratories:

- Ship all specimens overnight to CDC.
- Ship frozen specimens on dry ice and non-frozen specimens on cold packs.
- Refer to the International Air Transport Association (IATA - www.iata.org) for requirements for shipment of human or potentially infectious biological specimens. Follow shipping regulations for UN 3373 Biological Substance, Category B when sending potential 2019-nCoV specimens.
- Prior to shipping, notify CDC Division of Viral Diseases (see contact information below) that you are sending specimens.
- Send all samples to the following recipient:

Centers for Disease Control and Prevention
c/o STATT
Attention: Dr. Stephen Lindstrom (Unit 84)
1600 Clifton Rd., Atlanta, GA 30329-4027
Phone: (404) 639-3931

The emergency contact number for CDC Emergency Operations Center (EOC) is 770-488-7100.

All other laboratories that are CLIA certified and meet requirements to perform high complexity testing:

- Please notify your state and/or local public health laboratory for specimen referral and confirmatory testing guidance.

Reagent and Controls Preparation

NOTE: Storage information is for materials obtained through the CDC International Regent Resource. If using commercial products for testing, please refer to the manufacturer's instructions for storage, handling and preparation instructions.

Primer and Probe Preparation:

1) Upon receipt, store dried primers and probes at 2-8°C.
2) Precautions: These reagents should only be handled in a clean area and stored at appropriate temperatures (see below) in the dark. Freeze-thaw cycles should be avoided. Maintain cold when thawed.
3) Using aseptic technique, suspend dried reagents in 1.5 mL of nuclease-free water (50X working concentration) and allow to rehydrate for 15 min at room temperature in the dark.
4) Mix gently and aliquot primers/probe in 300 µL volumes into 5 pre-labeled tubes. Store a single aliquot of primers/probe at 2-8°C in the dark. Do not refreeze (stable for up to 4 months). Store remaining aliquots at ≤ -20°C in a non-frost-free freezer.

2019-nCoV Positive Control (nCoVPC) Preparation:

1) Precautions: This reagent should be handled with caution in a dedicated nucleic acid handling area to prevent possible contamination. Freeze-thaw cycles should be avoided. Maintain on ice when thawed.
2) Resuspend dried reagent in each tube in 1 mL of nuclease-free water to achieve the proper concentration. Make single use aliquots (approximately 30 µL) and store at ≤ -70ºC.
3) Thaw a single aliquot of diluted positive control for each experiment and hold on ice until adding to plate. Discard any unused portion of the aliquot.

Human Specimen Control (HSC) (not provided)

1) Human Specimen Control (HSC) or one of the listed acceptable alternative extraction controls must be extracted and processed with each specimen extraction run.
2) Refer to the Human Specimen Control (HSC) package insert for instructions for use.

No Template Control (NTC) (not provided)

1) Sterile, nuclease-free water
2) Aliquot in small volumes
3) Used to check for contamination during specimen extraction and/or plate set-up

General Preparation

Equipment Preparation

Clean and decontaminate all work surfaces, pipettes, centrifuges, and other equipment prior to use. Decontamination agents should be used including 10% bleach, 70% ethanol, and *DNAzap*™ or *RNase AWAY*® to minimize the risk of nucleic acid contamination.

Nucleic Acid Extraction

Performance of the CDC 2019-nCoV Real-Time RT-PCR Diagnostic Panel is dependent upon the amount and quality of template RNA purified from human specimens. The following commercially available RNA extraction kits and procedures have been qualified and validated for recovery and purity of RNA for use with the panel:

Qiagen QIAamp® DSP Viral RNA Mini Kit or QIAamp® Viral RNA Mini Kit

Recommendation(s): Utilize 100 µL of sample and elute with 100 µL of buffer or utilize 140 µL of sample and elute with 140 µL of buffer.

Qiagen EZ1 Advanced XL

Kit: Qiagen EZ1 DSP Virus Kit and Buffer AVL (supplied separately) for offboard lysis
Card: EZ1 Advanced XL DSP Virus Card
Recommendation(s): Add 120 µL of sample to 280 µL of pre-aliquoted Buffer AVL (total input sample volume is 400 µL). Proceed with the extraction on the EZ1 Advanced XL. Elution volume is 120 µL.

Kit: Qiagen EZ1 Virus Mini Kit v2.0 and Buffer AVL (supplied separately) for offboard lysis
Card: EZ1 Advanced XL Virus Card v2.0
Recommendation(s): Add 120 µL of sample to 280 µL of pre-aliquoted Buffer AVL (total input sample volume is 400 µL). Proceed with the extraction on the EZ1 Advanced XL. Elution volume is 120 µL.

Equivalence and performance of the following extraction platforms were demonstrated with the CDC Human Influenza Virus Real-Time RT-PCR Diagnostic Panel (K190302) and based on those data are acceptable for use with the CDC 2019-nCoV Real-Time RT-PCR Diagnostic Panel.

QIAGEN QIAcube
Kit: QIAGEN QIAamp® DSP Viral RNA Mini Kit or QIAamp® Viral RNA Mini Kit
Recommendations: Utilize 140 µL of sample and elute with 100 µL of buffer.

Roche MagNA Pure LC
Kit: Roche MagNA Pure Total Nucleic Acid Kit
Protocol: Total NA External_lysis
Recommendation(s): Add 100 µL of sample to 300 µL of pre-aliquoted TNA isolation kit lysis buffer (total input sample volume is 400 µL). Elution volume is 100 µL.

Roche MagNA Pure Compact
Kit: Roche MagNA Pure Nucleic Acid Isolation Kit I
Protocol: Total_NA_Plasma100_400
Recommendation(s): Add 100 µL of sample to 300 µL of pre-aliquoted TNA isolation kit lysis buffer (total input sample volume is 400 µL). Elution volume is 100 µL.

Roche MagNA Pure 96
Kit: Roche MagNA Pure 96 DNA and Viral NA Small Volume Kit
Protocol: Viral NA Plasma Ext Lys SV Protocol
Recommendation(s): Add 100 µL of sample to 350 µL of pre-aliquoted External Lysis Buffer (supplied separately) (total input sample volume is 450 µL). Proceed with the extraction on the MagNA Pure 96. (**Note: Internal Control = None)**. Elution volume is 100 µL.

bioMérieux NucliSENS® easyMAG® Instrument
Protocol: General protocol (not for blood) using "Off-board Lysis" reagent settings.
Recommendation(s): Add 100 µL of sample to 1000 µL of pre-aliquoted easyMAG lysis buffer (total input sample volume is 1100 µL). Incubate for 10 minutes at room temperature. Elution volume is 100 µL.

bioMérieux EMAG® Instrument
Protocol: Custom protocol: **CDC Flu V1** using "Off-board Lysis" reagent settings.
Recommendation(s): Add 100 µL of samples to 2000 µL of pre-aliquoted easyMAG lysis buffer (total input sample volume is 2100 µL). Incubate for 10 minutes at room temperature. Elution volume is 100 µL. The custom protocol, **CDC Flu V1**, is programmed on the bioMérieux EMAG® instrument with the assistance of a bioMérieux service representative. Installation verification is documented at the time of installation. Laboratories are recommended to retain a record of the step-by-step verification of the bioMérieux custom protocol installation procedure.

Manufacturer's recommended procedures (except as noted in recommendations above) are to be followed for sample extraction. HSC must be included in each extraction batch.

Disclaimer: Names of vendors or manufacturers are provided as examples of suitable product sources. Inclusion does not imply endorsement by the Centers for Disease Control and Prevention.

Assay Set Up

Reaction Master Mix and Plate Set Up

Note: Plate set-up configuration can vary with the number of specimens and workday organization. NTCs and nCoVPCs must be included in each run.

1) In the reagent set-up room clean hood, place rRT-PCR buffer, enzyme, and primer/probes on ice or cold-block. Keep cold during preparation and use.
2) Mix buffer, enzyme, and primer/probes by inversion 5 times.
3) Centrifuge reagents and primers/probes for 5 seconds to collect contents at the bottom of the tube, and then place the tube in a cold rack.
4) Label one 1.5 mL microcentrifuge tube for each primer/probe set.
5) Determine the number of reactions (N) to set up per assay. It is necessary to make excess reaction mix for the NTC, nCoVPC, HSC (if included in the RT-PCR run), and RP reactions and for pipetting error. Use the following guide to determine N:
   - If number of samples (n) including controls equals 1 through 14, then N = n + 1
   - If number of samples (n) including controls is 15 or greater, then N = n + 2
7) For each primer/probe set, calculate the amount of each reagent to be added for each reaction mixture (N = # of reactions).

Thermofisher TaqPath™ 1-Step RT-qPCR Master Mix

Step #	Reagent	Vol. of Reagent Added per Reaction
1	Nuclease-free Water	N x 8.5 µL
2	Combined Primer/Probe Mix	N x 1.5 µL
3	TaqPath™ 1-Step RT-qPCR Master Mix (4x)	N x 5.0 µL
	**Total Volume**	**N x 15.0 µL**

Promega GoTaq® Probe 1- Step RT-qPCR System

Step #	Reagent	Vol. of Reagent Added per Reaction
1	Nuclease-free Water	N x 3.1 µL
2	Combined Primer/Probe Mix	N x 1.5 µL
3	GoTaq Probe qPCR Master Mix with dUTP	N x 10.0 µL
4	Go Script RT Mix for 1-Step RT-qPCR	N x 0.4 µL
	**Total Volume**	**N x 15.0 µL**

Quantabio qScript XLT One-Step RT-qPCR ToughMix

Step #	Reagent	Vol. of Reagent Added per Reaction
1	Nuclease-free Water	N x 3.5 µL
2	Combined Primer/Probe Mix	N x 1.5 µL
3	qScript XLT One-Step RT-qPCR ToughMix (2X)	N x 10.0 µL
	**Total Volume**	**N x 15.0 µL**

Quantabio UltraPlex 1-Step ToughMix (4X)

Step #	Reagent	Vol. of Reagent Added per Reaction
1	Nuclease-free Water	N x 8.5 µL
2	Combined Primer/Probe Mix	N x 1.5 µL
3	UltraPlex 1-Step ToughMix (4X)	N x 5.0 µL
	**Total Volume**	**N x 15.0 µL**

8) Dispense reagents into each respective labeled 1.5 mL microcentrifuge tube. After addition of the reagents, mix reaction mixtures by pipetting up and down. ***Do not vortex***.
9) Centrifuge for 5 seconds to collect contents at the bottom of the tube, and then place the tube in a cold rack.
10) Set up reaction strip tubes or plates in a 96-well cooler rack.
11) Dispense 15 µL of each master mix into the appropriate wells going across the row as shown below (**Figure 1**):

Figure 1: Example of Reaction Master Mix Plate Set-Up

	1	2	3	4	5	6	7	8	9	10	11	12
**A**	N1	N1	N1	N1	N1	N1	N1	N1	N1	N1	N1	N1
**B**	N2	N2	N2	N2	N2	N2	N2	N2	N2	N2	N2	N2
**C**	RP	RP	RP	RP	RP	RP	RP	RP	RP	RP	RP	RP
**D**												
**E**												
**F**												
**G**												
**H**												

12) Prior to moving to the nucleic acid handling area, prepare the No Template Control (NTC) reactions for column #1 in the assay preparation area.
13) Pipette 5 µL of nuclease-free water into the NTC sample wells (**Figure 2**, column 1). Securely cap NTC wells before proceeding.
14) Cover the entire reaction plate and move the reaction plate to the specimen nucleic acid handling area.

<u>Nucleic Acid Template Addition</u>

1) Gently vortex nucleic acid sample tubes for approximately 5 seconds.
2) Centrifuge for 5 seconds to collect contents at the bottom of the tube.
3) After centrifugation, place extracted nucleic acid sample tubes in the cold rack.
4) Samples should be added to columns 2-11 (column 1 and 12 are for controls) to the specific assay that is being tested as illustrated in **Figure 2**. Carefully pipette 5.0 µL of the first sample into all the wells labeled for that sample (i.e. Sample "S1" down column #2). *Keep other sample wells covered during addition. Change tips after each addition.*
5) Securely cap the column to which the sample has been added to prevent cross contamination and to ensure sample tracking.
6) Change gloves often and when necessary to avoid contamination.
7) Repeat steps #4 and #5 for the remaining samples.

8) If necessary, add 5 µL of Human Specimen Control (HSC) extracted sample to the HSC wells (**Figure 2**, column 11). Securely cap wells after addition. NOTE: Per CLIA regulations, HSC must be tested at least once per day.
9) Cover the entire reaction plate and move the reaction plate to the positive template control handling area.

Assay Control Addition

1) Pipette 5 µL of nCoVPC RNA to the sample wells of column 12 (**Figure 2**)**.** Securely cap wells after addition of the control RNA.

NOTE: *If using 8-tube strips, label the TAB of each strip to indicate sample position.* ***DO NOT LABEL THE TOPS OF THE REACTION TUBES!***

2) Briefly centrifuge reaction tube strips for 10-15 seconds. After centrifugation return to cold rack.

NOTE: *If using 96-well plates, centrifuge plates for 30 seconds at 500 x g, 4°C.*

Figure 2. 2019-nCoV rRT-PCR Diagnostic Panel: Example of Sample and Control Set-up

	1	2	3	4	5	6	7	8	9	10	11[a]	12
A	NTC	S1	S2	S3	S4	S5	S6	S7	S8	S9	S10	nCoV PC
B	NTC	S1	S2	S3	S4	S5	S6	S7	S8	S9	S10	nCoV PC
C	NTC	S1	S2	S3	S4	S5	S6	S7	S8	S9	S10	nCoV PC
D												
E												
F												
G												
H												

[a]Replace the sample in this column with extracted HSC if necessary

Create a Run Template on the Applied Biosystems 7500 Fast Dx Real-time PCR Instrument (Required if no template exists)

If the template already exists on your instrument, please proceed to the **RUNNING A TEST** section.

1) Launch the Applied Biosystems 7500 Fast Dx Real-time PCR Instrument by double clicking on the Applied Biosystems 7500 Fast Dx System icon on the desktop.
2) A new window should appear, select **Create New Document** from the menu.

Figure 3. New Document Wizard Window

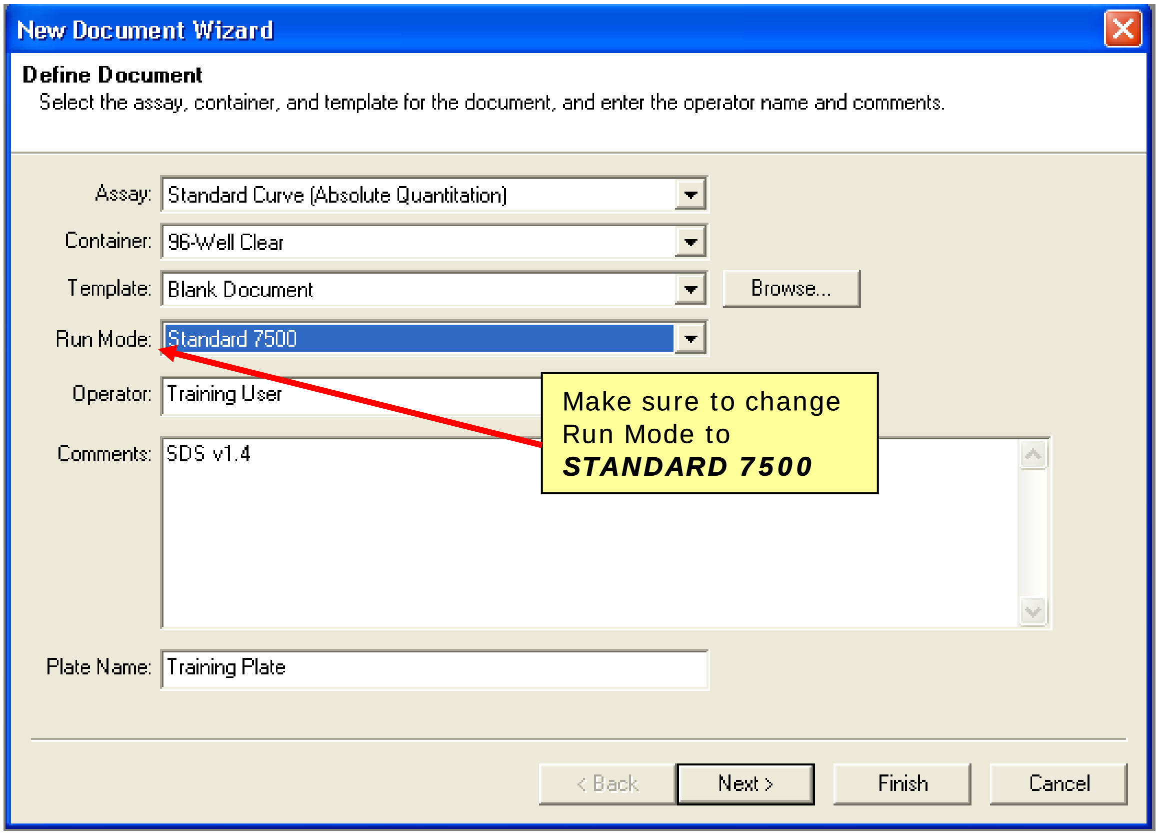

3) The **New Document Wizard** screen in **Figure 3** will appear. Select:
   a. Assay: **Standard Curve (Absolute Quantitation)**
   b. Container: **96-Well Clear**
   c. Template: **Blank Document**
   d. Run Mode: **Standard 7500**
   e. Operator: ***Your Name***
   f. Comments: **SDS v1.4**
   g. Plate Name: ***Your Choice***
4) After making selections click **Next** at the bottom of the window.

Figure 4. Creating New Detectors

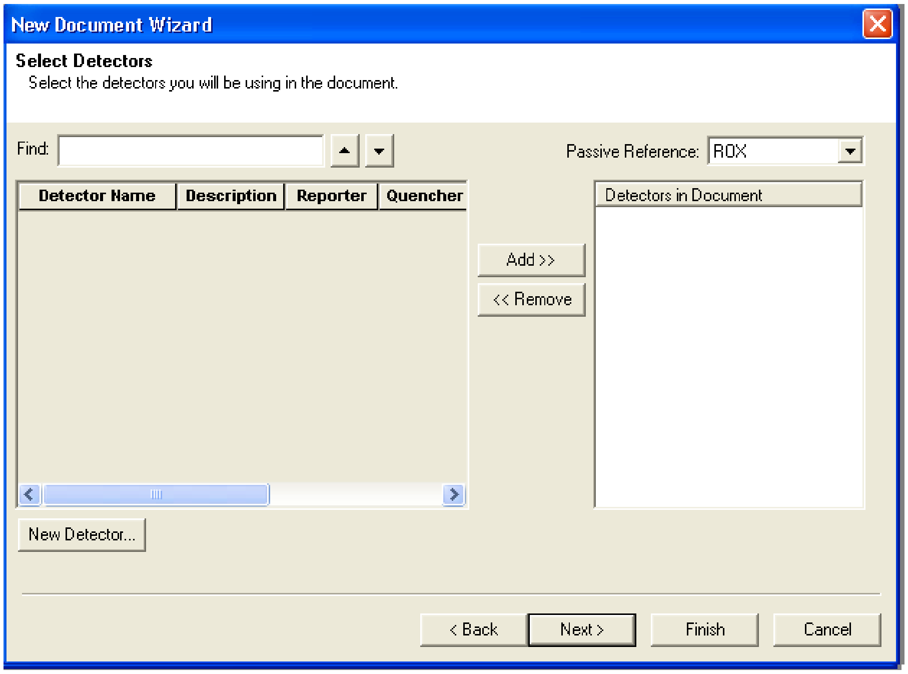

5) After selecting next, the ***Select Detectors*** screen (**Figure 4**) will appear.
6) Click the **New Detector** button (see **Figure 4**).
7) The **New Detector** window will appear (**Figure 5**). A new detector will need to be defined for each primer and probe set. Creating these detectors will enable you to analyze each primer and probe set individually at the end of the reaction.

Figure 5. New Detector Window

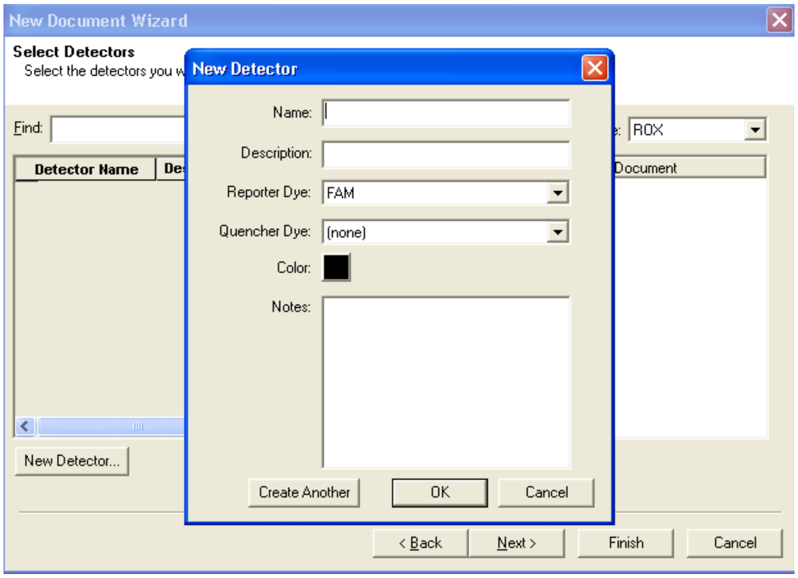

8) Start by creating the N1 Detector. Include the following:
   a. Name: **N1**
   b. Description: *leave blank*
   c. Reporter Dye: **FAM**
   d. Quencher Dye: **(none)**
   e. Color: *to change the color of the detector indicator do the following:*
      ⇒ Click on the color square to reveal the color chart
      ⇒ Select a color by clicking on one of the squares
      ⇒ After selecting a color click **OK** to return to the New Detector screen
   f. Click the **OK** button of the New Detector screen to return to the screen shown in **Figure 4**.
9) Repeat step 6-8 for each target in the panel.

Name	Reporter Dye	Quencher Dye
N1	FAM	(none)
N2	FAM	(none)
RP	FAM	(none)

10) After each Detector is added, the **Detector Name**, **Description**, **Reporter** and **Quencher** fields will become populated in the **Select Detectors** screen (**Figure 6**).
11) Before proceeding, the newly created detectors must be added to the document. To add the new detectors to the document, click **ADD** (see **Figure 6**). Detector names will appear on the right-hand side of the **Select Detectors** window (**Figure 6**).

Figure 6. Adding New Detectors to Document

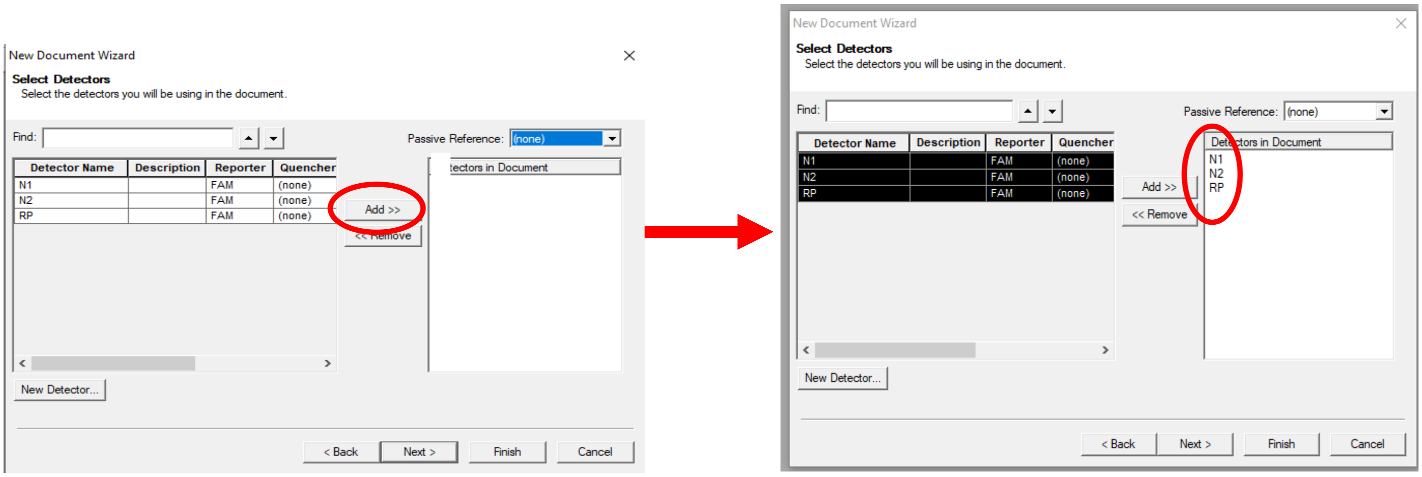

12) Once all detectors have been added, select **(none)** for **Passive Reference** at the top right-hand drop-down menu (**Figure 7**).

Figure 7. Select Passive Reference

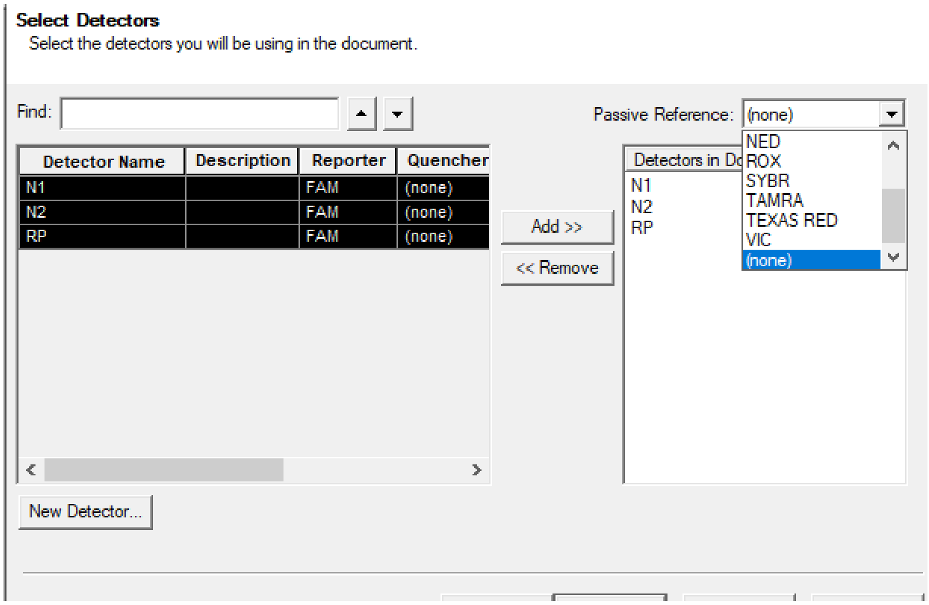

Passive reference should be set to "(none)" as described above.

13) Click **Next** at the bottom of the **Select Detectors** window to proceed to the **Set Up Sample Plate** window (**Figure 8**).

14) In the **Set Up Sample Plate** window (**Figure 8**), use your mouse to select row A from the lower portion of the window, in the spreadsheet (see **Figure 8**).

15) In the top portion of the window, select detector **N1**. A check will appear next to the detector you have selected (**Figure 8**). You will also notice the row in the spreadsheet will be populated with a colored "U" icon to indicate which detector you've selected.

16) Repeat step 14-15 for each detector that will be used in the assay.

Figure 8. Sample Plate Set-up

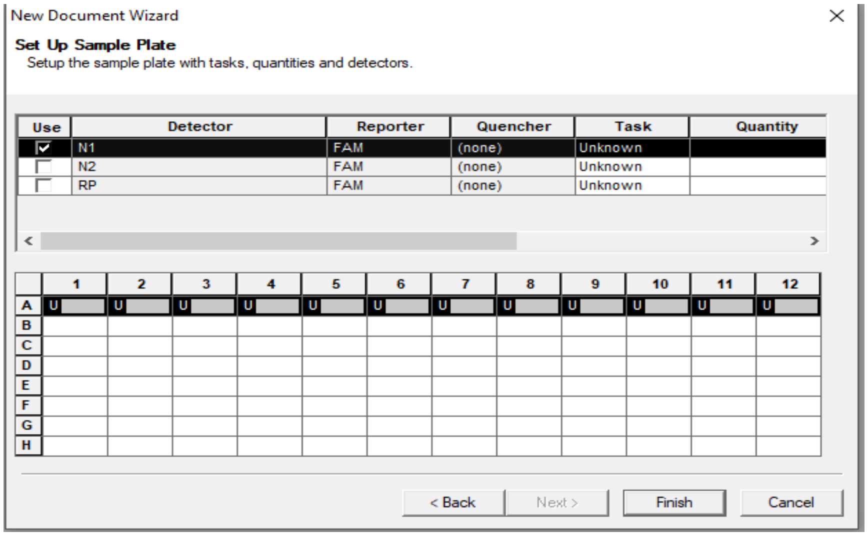

17) Select **Finish** after detectors have been assigned to their respective rows. (**Figure 9**).

Figure 9. Finished Plate Set-up

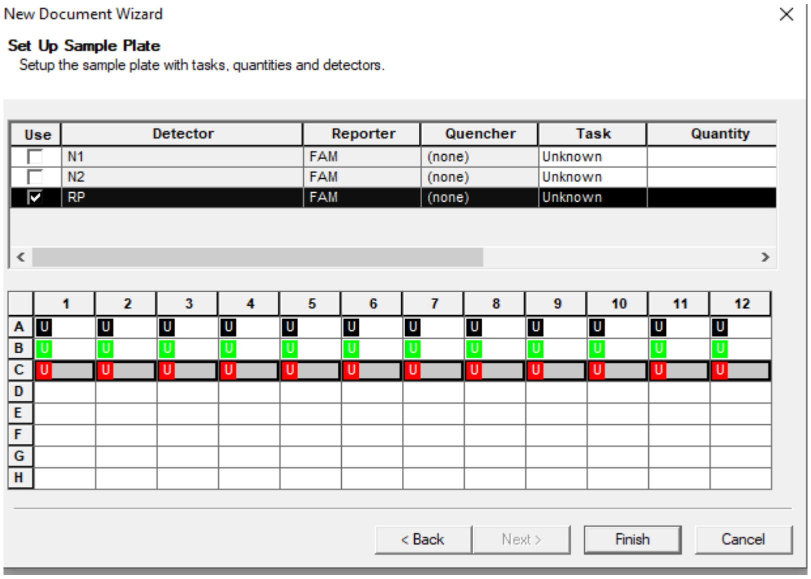

18) After clicking “Finish”, there will be a brief pause allowing the Applied Biosystems 7500 Fast Dx to initialize. This initialization is followed by a clicking noise. ***Note: The machine must be turned on for initialization.***
19) After initialization, the **Plate** tab of the Setup (**Figure 10)** will appear.
20) Each well of the plate should contain colored U icons that correspond with the detector labels that were previously chosen. To confirm detector assignments, select **Tools** from the file menu, then select **Detector Manager.**

Figure 10. Plate Set-up Window

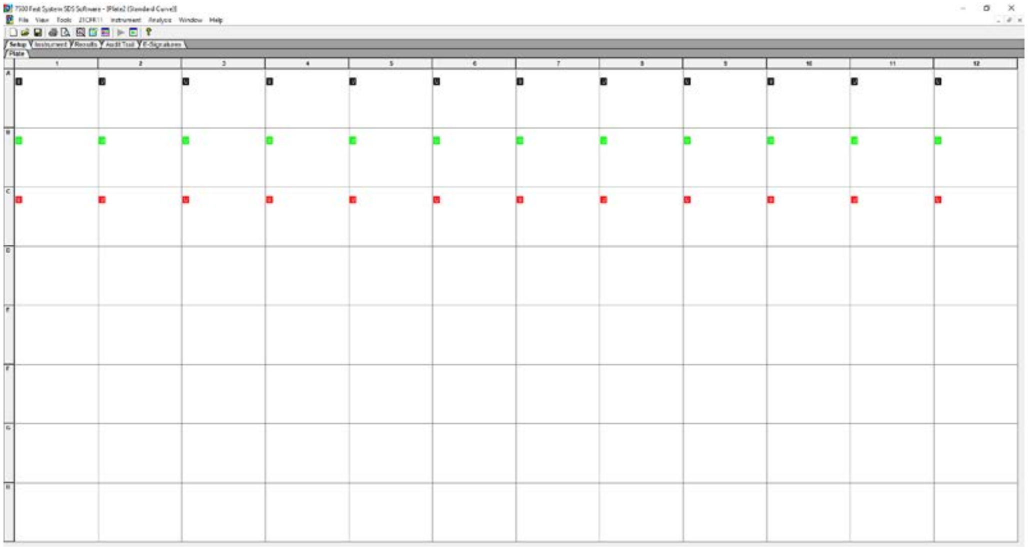

21) The Detector Manager window will appear (**Figure 11**).

Figure 11. Detector Manager Window

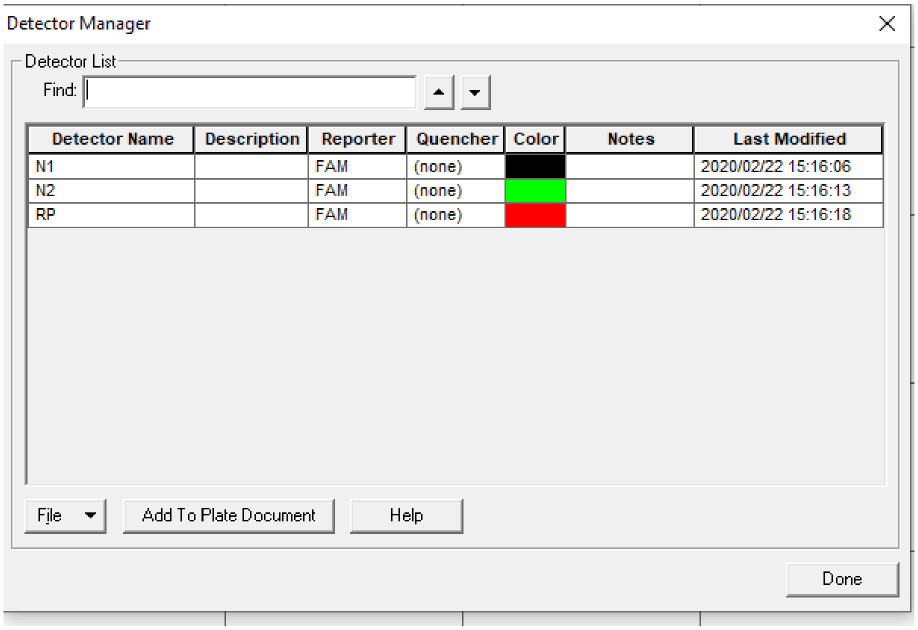

22) Confirm all detectors are included and that each target has a **Reporter** set to **FAM** and the **Quencher** is set to **(none)**.
23) If all detectors are present, select **Done**. The detector information has been created and assigned to wells on the plate.

Defining the Instrument Settings

1) After detectors have been created and assigned, proceed to instrument set up.
2) Select the **Instrument** tab to define thermal cycling conditions.
3) Modify the thermal cycling conditions as follows (**Figure 12**):

Thermofisher TaqPath™ 1-Step RT-qPCR Master Mix, CG

a. In Stage 1, Set to 2 min at **25°C**; **1 Rep**.
b. In Stage 2, Set to 15 min at **50°C**; **1 Rep**.
c. In Stage 3, Set to 2 min at **95°C, 1 Rep.**
d. In Stage 4, Step 1 set to **3 sec** at **95°C**.
e. In Stage 4, Step 2 set to **30 sec** at **55.0°C.**
f. In Stage 4, Reps should be set to **45.**
g. Under **Settings** (**Figure 12**), bottom left-hand box, change volume to 20 µL.
h. Under **Settings**, **Run Mode** selection should be **Standard 7500**.
i. Step 2 of Stage 4 should be highlighted in yellow to indicate data collection (see **Figure 12**).

OR

Quantabio qScript™ XLT One-Step RT-qPCR ToughMix or UltraPlex 1-Step ToughMix

a. In Stage 1, Set to 10 min at **50°C**; **1 Rep**.
b. In Stage 2, Set to 3 min at **95°C, 1 Rep.**
c. In Stage 3, Step 1 set to **3 sec** at **95°C**.
d. In Stage 3, Step 2 set to **30 sec** at **55.0°C.**
e. In Stage 3, Reps should be set to **45.**
f. Under **Settings** (**Figure 12**), bottom left-hand box, change volume to 20 µL.
g. Under **Settings**, **Run Mode** selection should be **Standard 7500**.
h. Step 2 of Stage 4 should be highlighted in yellow to indicate data collection (see **Figure 12**).

OR

Promega GoTaq® Probe 1-Step RT-qPCR System

a. In Stage 1, Set to 15 min at **45°C**; **1 Rep**.
b. In Stage 2, Set to 2 min at **95°C, 1 Rep.**
c. In Stage 3, Step 1 set to **3 sec** at **95°C**.
d. In Stage 3, Step 2 set to **30 sec** at **55.0°C.**
e. In Stage 3, Reps should be set to **45.**
f. Under **Settings** (**Figure 12**), bottom left-hand box, change volume to 20 µL.
g. Under **Settings**, **Run Mode** selection should be **Standard 7500**.
h. Step 2 of Stage 4 should be highlighted in yellow to indicate data collection (see **Figure 12**).

Figure 12. Instrument Window

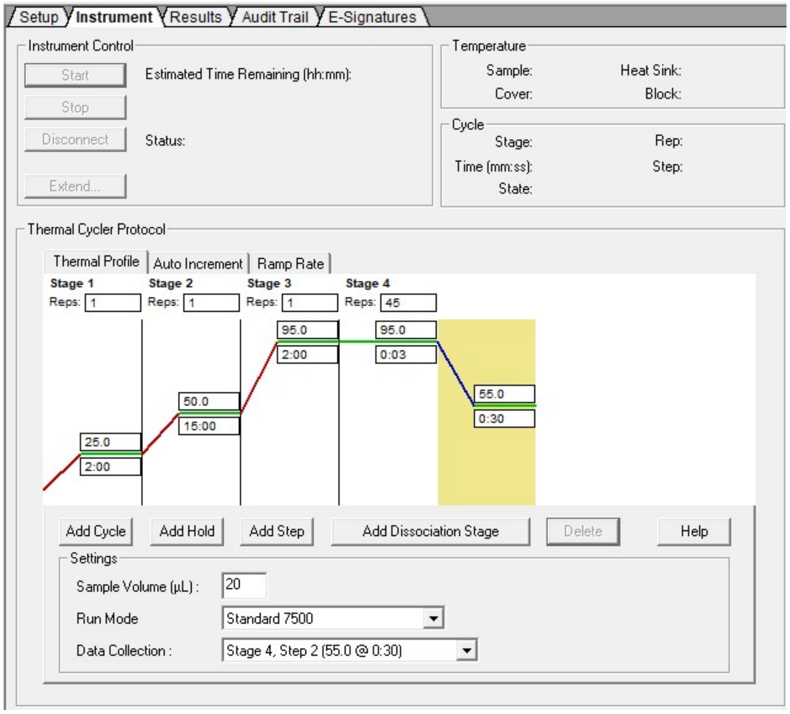

4) After making changes to the **Instrument** tab, the template file is ready to be saved. To save the template, select **File** from the top menu, then select **Save As**. Since the enzyme options have different instrument settings, it is recommended that the template be saved with a name indicating the enzyme option.

5) Save the template as **2019-nCoV Dx Panel TaqPath** or **2019-nCoV Dx Panel Quanta** or **2019-nCoV Dx Panel Promega** as appropriate in the desktop folder labeled "***ABI Run Templates***" (*you must create this folder*). Save as type should be SDS Templates (*.sdt) (**Figure 13**).

Figure 13. Saving Template

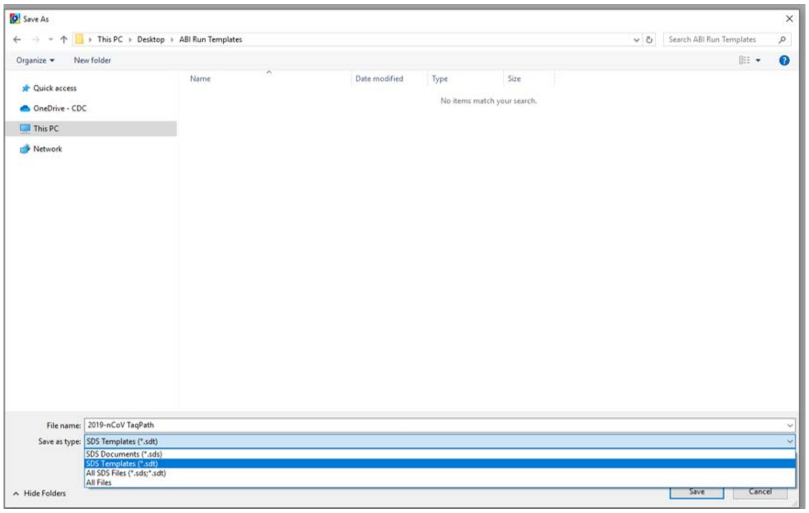

Running a Test

1) Turn on the ABI 7500 Fast Dx Real-Time PCR Instrument.
2) Launch the Applied Biosystems 7500 Fast Dx Real-time PCR System by double clicking on the 7500 Fast Dx System icon on the desktop.
3) A new window should appear, select **Open Existing Document** from the menu.
4) Navigate to select your ABI Run Template folder from the desktop.
5) Double click on the appropriate template file (**2019-nCoV Dx Panel TaqPath** or **2019-nCoV Dx Panel Quanta** or **2019-nCoV Dx Panel Promega)**
6) There will be a brief pause allowing the Applied Biosystems 7500 Fast Dx Real-Time PCR Instrument to initialize. This initialization is followed by a clicking noise. ***Note: The machine must be turned on for initialization.***

Figure 14. Plate Set-up Window

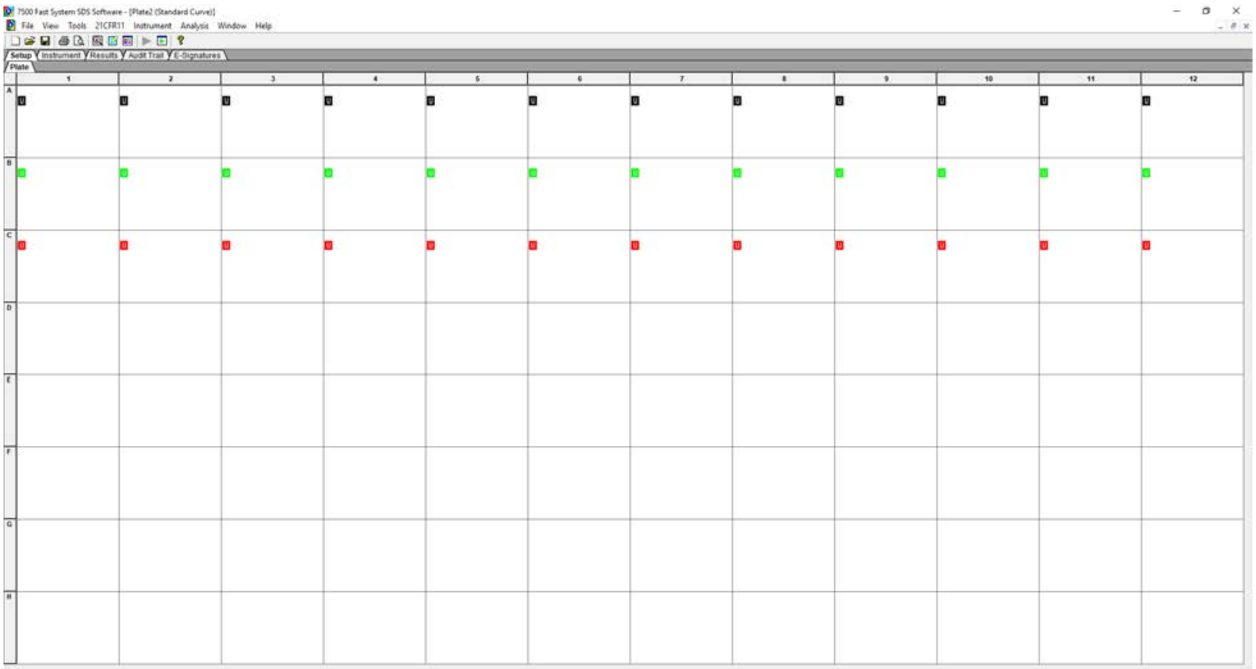

7) After the instrument initializes, a plate map will appear (**Figure 14**). The detectors and controls should already be labeled as they were assigned in the original template.

8) Click the **Well Inspector** icon 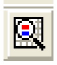 from the top menu.
9) Highlight specimen wells of interest on the plate map.
10) Type sample identifiers to **Sample Name** box in the **Well Inspector** window (**Figure 15**).

Figure 15. Labeling Wells

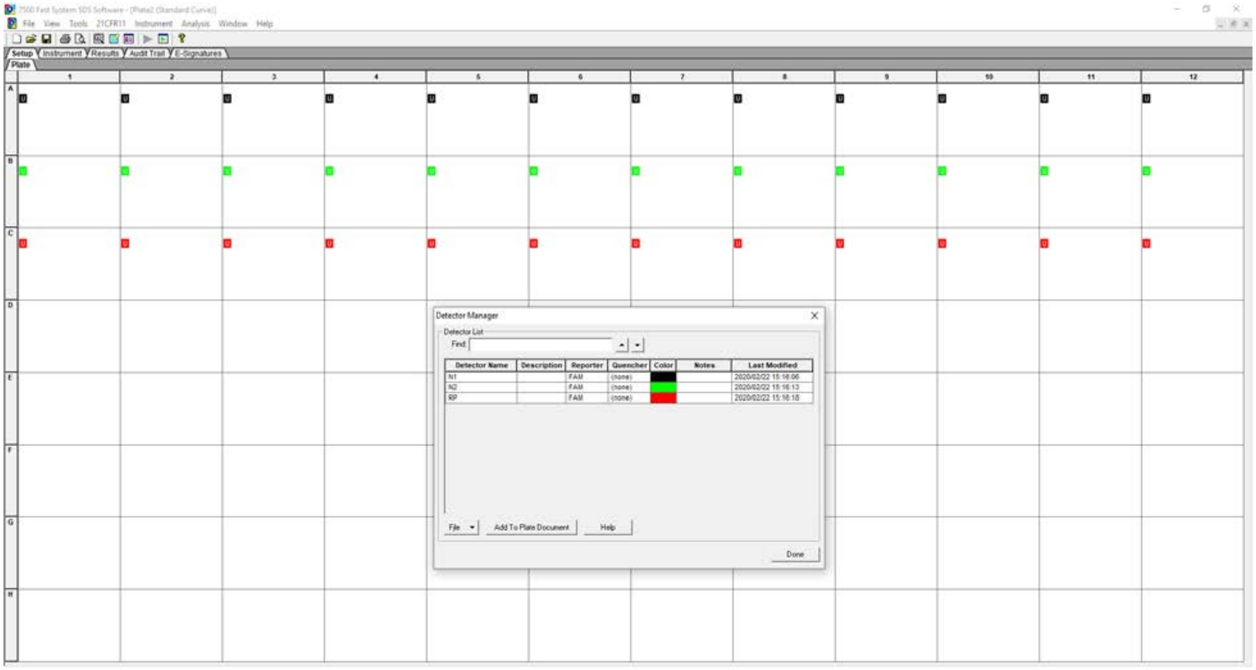

11) Repeat steps 9-10 until all sample identifiers are added to the plate setup.

12) Once all specimen and control identifiers are added click the **Close** button on the **Well Inspector** window to return to the **Plate** set up tab.
13) Click the **Instrument** tab at the upper left corner.
14) The reaction conditions, volumes, and type of 7500 reaction should already be loaded (**Figure 16**).

Figure 16. Instrument Settings

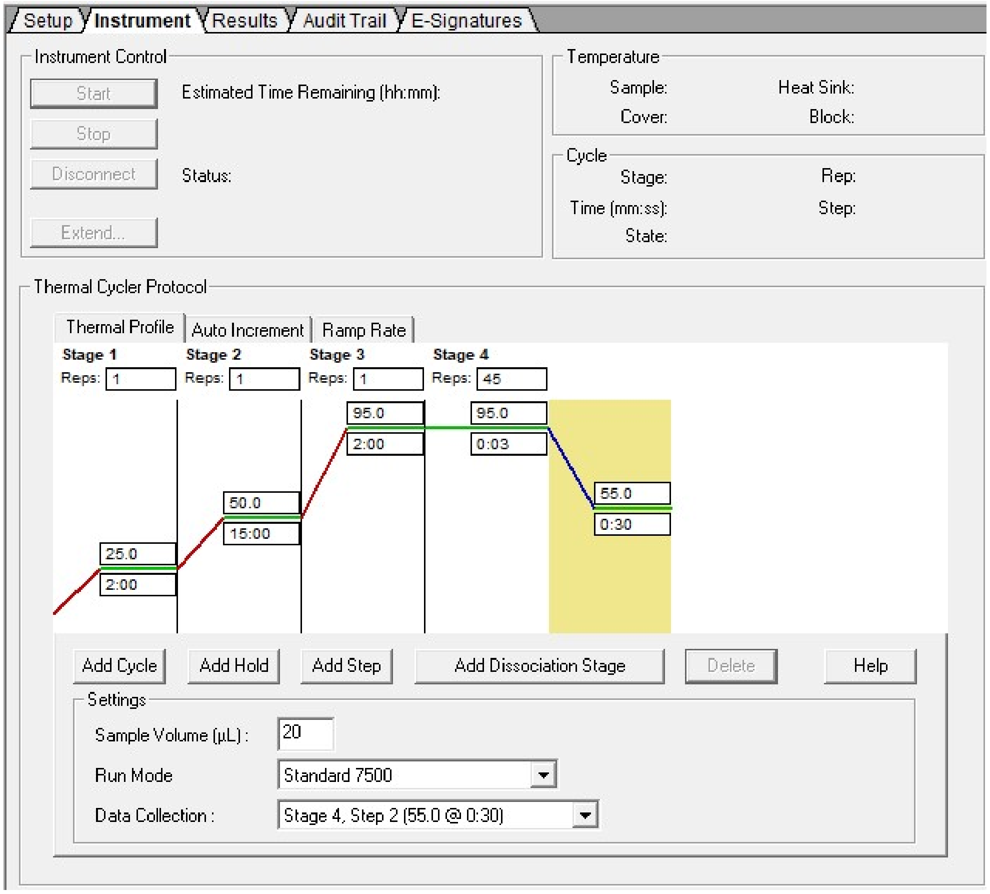

15) Ensure settings are correct (refer to the *Defining Instrument Settings*).
16) Before proceeding, the run file must be saved; from the main menu, select **File,** then **Save As**. Save in appropriate run folder designation.
17) Load the plate into the plate holder in the instrument. Ensure that the plate is properly aligned in the holder.
18) Once the run file is saved, click the **Start** button. *Note: The run should take approximately 1hr and 20 minutes to complete.*

Data Analysis

1) After the run has completed, select the **Results** tab at the upper left corner of the software.
2) Select the **Amplification Plot** tab to view the raw data (**Figure 17**).

Figure 17. Amplification Plot Window

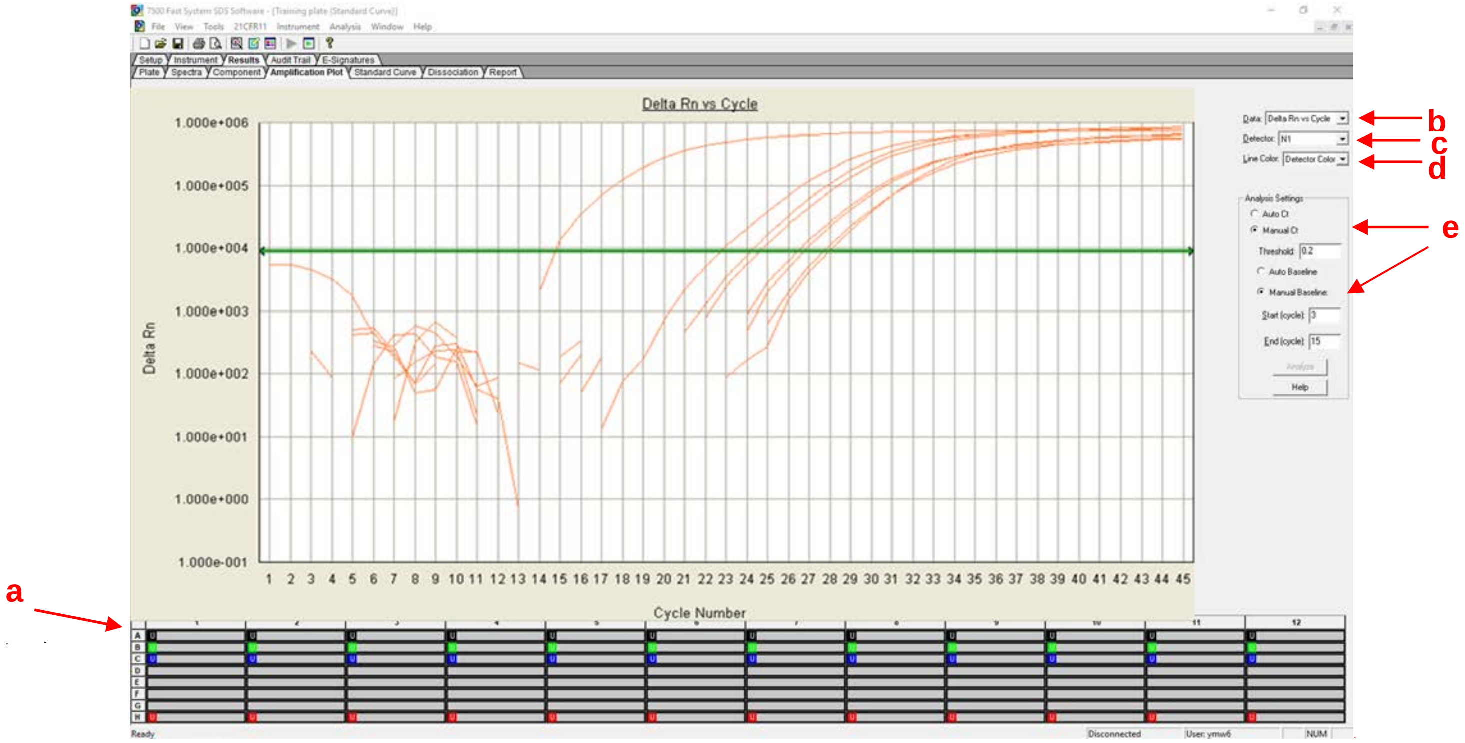

3) Start by highlighting all the samples from the run; to do this, click on the upper left-hand box **(a)** of the sample wells (**Figure 17**). All the growth curves should appear on the graph.
4) On the right-hand side of the window **(b)**, the **Data** drop down selection should be set to **Delta Rn vs. Cycle**.
5) Select **N1** from **(c)**, the **Detector** drop down menu, using the downward arrow.
   a. Please note that each detector is analyzed individually to reflect different performance profiles of each primer and probe set.
6) In the **Line Color** drop down **(d)**, **Detector Color** should be selected.
7) Under **Analysis Settings** select **Manual Ct (e)**.
   b. Do not change the **Manual Baseline** default numbers.
8) Using the mouse, click and drag the red threshold line until it lies within the exponential phase of the fluorescence curves and above any background signal (**Figure 18)**.

Figure 18. Amplification Plot

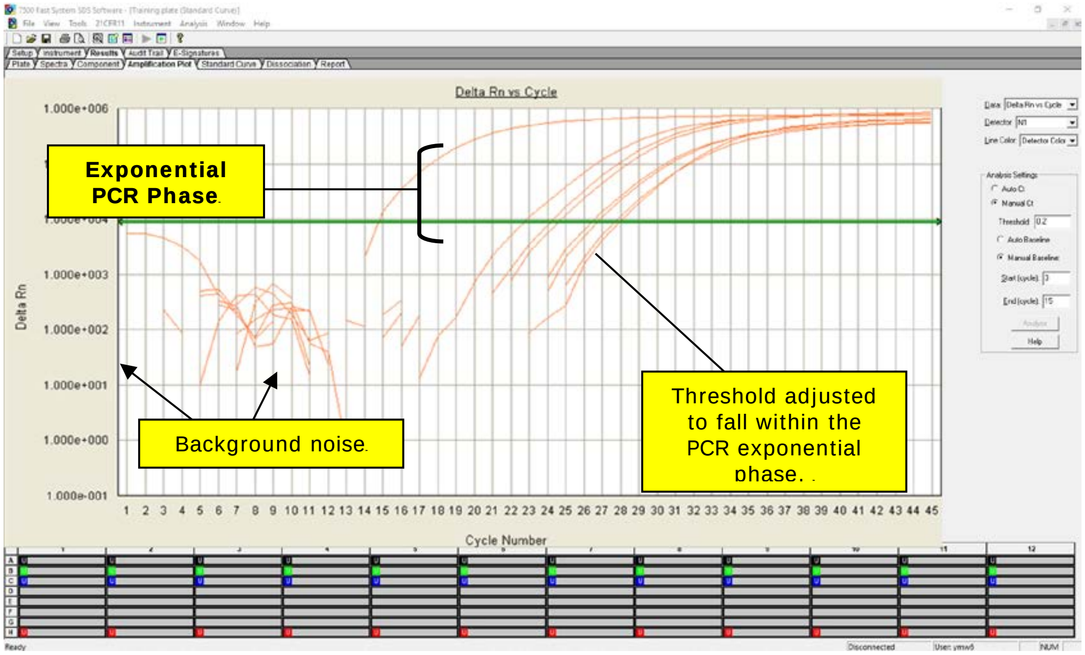

9) Click the **Analyze** button in the lower right corner of the window. The red threshold line will turn to green, indicating the data has been analyzed.
10) Repeat steps 5-9 to analyze results generated for each set of markers (N1, N2, RP).
11) Save analysis file by selecting **File** then **Save As** from the main menu.
12) After completing analysis for each of the markers, select the **Report** tab above the graph to display the Ct values (**Figure 19**). To filter report by sample name in ascending or descending order, simply click on **Sample Name** in the table.

Figure 19. Report

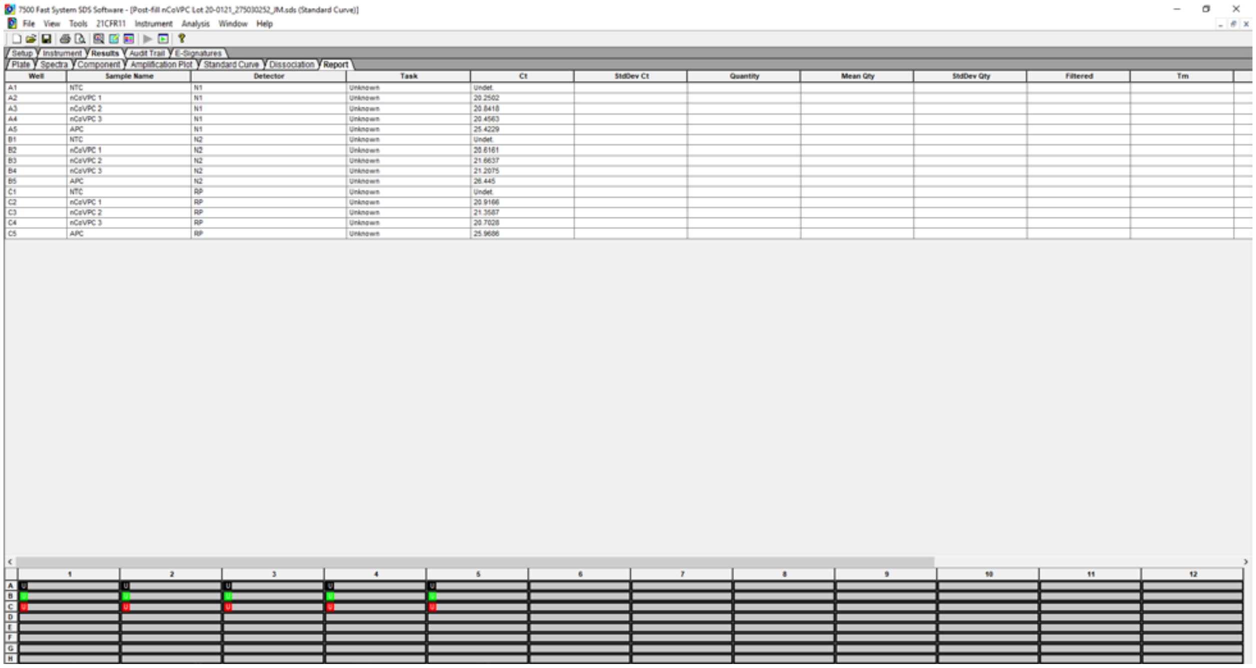

Well	Sample Name	Detector	Task	Ct	StdDev Ct	Quantity	Mean Qty	StdDev Qty	Filtered	Tm
A1	NTC	N1	Unknown	Undet.						
A2	nCoVPC 1	N1	Unknown	20.2502						
A3	nCoVPC 2	N1	Unknown	20.8418						
A4	nCoVPC 3	N1	Unknown	20.4563						
A5	APC	N1	Unknown	25.4229						
B1	NTC	N2	Unknown	Undet.						
B2	nCoVPC 1	N2	Unknown	20.6161						
B3	nCoVPC 2	N2	Unknown	21.6637						
B4	nCoVPC 3	N2	Unknown	21.2075						
B5	APC	N2	Unknown	26.445						
C1	NTC	RP	Unknown	Undet.						
C2	nCoVPC 1	RP	Unknown	20.9166						
C3	nCoVPC 2	RP	Unknown	21.3587						
C4	nCoVPC 3	RP	Unknown	20.7028						
C5	APC	RP	Unknown	25.9686						

Interpretation of Results and Reporting

Extraction and Positive Control Results and Interpretation

No Template Control (NTC)

The NTC consists of using nuclease-free water in the rRT-PCR reactions instead of RNA. The NTC reactions for all primer and probe sets should not exhibit fluorescence growth curves that cross the threshold line. If any of the NTC reactions exhibit a growth curve that crosses the cycle threshold, sample contamination may have occurred. Invalidate the run and repeat the assay with strict adherence to the guidelines.

2019-nCoV Positive Control (nCoVPC)

The nCoVPC consists of in vitro transcribed RNA. The nCoVPC will yield a positive result with the following primer and probe sets: N1, N2 and RP.

Human Specimen Control (HSC) (Extraction Control)

When HSC is run with the CDC 2019-nCoV rRT-PCR Diagnostic Panel (see previous section on Assay Set Up), the HSC is used as an RNA extraction procedural control to demonstrate successful recovery of RNA as well as extraction reagent integrity. The HSC control consists of noninfectious cultured human cell (A549) material. Purified nucleic acid from the HSC should yield a positive result with the RP primer and probe set and negative results with all 2019-nCoV markers.

Expected Performance of Controls Included in the CDC 2019-nCoV Real-Time RT-PCR Diagnostic Panel

Control Type	External Control Name	Used to Monitor	2019 nCoV_N1	2019 nCoV_N2	RP	Expected Ct Values
Positive	nCoVPC	Substantial reagent failure including primer and probe integrity	+	+	+	< 40.00 Ct
Negative	NTC	Reagent and/or environmental contamination	-	-	-	None detected
Extraction	HSC	Failure in lysis and extraction procedure, potential contamination during extraction	-	-	+	< 40.00 Ct

If any of the above controls do not exhibit the expected performance as described, the assay may have been set up and/or executed improperly, or reagent or equipment malfunction could have occurred. Invalidate the run and re-test.

RNase P (Extraction Control)

- All clinical samples should exhibit fluorescence growth curves in the RNase P reaction that cross the threshold line within 40.00 cycles (< 40.00 Ct), thus indicating the presence of the human RNase P gene. Failure to detect RNase P in any clinical specimens may indicate:
  - Improper extraction of nucleic acid from clinical materials resulting in loss of RNA and/or RNA degradation.
  - Absence of sufficient human cellular material due to poor collection or loss of specimen integrity.
  - Improper assay set up and execution.
  - Reagent or equipment malfunction.
- If the RP assay does not produce a positive result for human clinical specimens, interpret as follows:
  - If the 2019-nCoV N1 and N2are positive even in the absence of a positive RP, the result should be considered valid. It is possible, that some samples may fail to exhibit RNase P growth curves due to low cell numbers in the original clinical sample. A negative RP signal does not preclude the presence of 2019-nCoV virus RNA in a clinical specimen.
  - If all 2019-nCoV markers AND RNase P are negative for the specimen, the result should be considered invalid for the specimen. If residual specimen is available, repeat the extraction procedure and repeat the test. If all markers remain negative after re-test, report the results as invalid and a new specimen should be collected if possible.

2019-nCoV Markers (N1 and N2)

- When all controls exhibit the expected performance, a specimen is considered negative if all 2019-nCoV marker (N1, N2) cycle threshold growth curves DO NOT cross the threshold line within 40.00 cycles (< 40.00 Ct) AND the RNase P growth curve DOES cross the threshold line within 40.00 cycles (< 40.00 Ct).
- When all controls exhibit the expected performance, a specimen is considered positive for 2019-nCoV if all 2019-nCoV marker (N1, N2) cycle threshold growth curves cross the threshold line within 40.00 cycles (< 40.00 Ct). The RNase P may or may not be positive as described above, but the 2019-nCoV result is still valid.
- When all controls exhibit the expected performance and the growth curves for the 2019-nCoV markers (N1, N2) AND the RNase P marker DO NOT cross the cycle threshold growth curve within 40.00 cycles (< 40.00 Ct), the result is invalid. The extracted RNA from the specimen should be re-tested. If residual RNA is not available, re-extract RNA from residual specimen and re-test. If the re-tested sample is negative for all markers and RNase P, the result is invalid and collection of a new specimen from the patient should be considered.
- When all controls exhibit the expected performance and the cycle threshold growth curve for any one marker (N1 or N2 but not both markers) crosses the threshold line within 40.00 cycles (< 40.00 Ct) the result is inconclusive. The extracted RNA should be retested. If residual RNA is not available, re-extract RNA from residual specimen and re-test. If the same result is obtained, report the inconclusive result. Consult with your state public health laboratory or CDC, as appropriate, to request guidance and/or to coordinate transfer of the specimen for additional analysis.
- If HSC is positive for N1 or N2, then contamination may have occurred during extraction or sample processing. Invalidate all results for specimens extracted alongside the HSC. Re-extract specimens and HSC and re-test.

2019-nCoV rRT-PCR Diagnostic Panel Results Interpretation Guide

The table below lists the expected results for the 2019-nCoV rRT-PCR Diagnostic Panel. If a laboratory obtains unexpected results for assay controls or if inconclusive or invalid results are obtained and cannot be resolved through the recommended re-testing, please contact CDC for consultation and possible specimen referral. See pages 10 and 40 for referral and contact information.

2019 nCoV_N1	2019 nCoV_N2	RP	Result Interpretation[a]	Report	Actions
+	+	±	2019-nCoV detected	Positive 2019-nCoV	Report results to CDC and sender.
If only one of the two targets is positive		±	Inconclusive Result	Inconclusive	Repeat testing of nucleic acid and/or re-extract and repeat rRT-PCR. If the repeated result remains inconclusive, contact your State Public Health Laboratory or CDC for instructions for transfer of the specimen or further guidance.
-	-	+	2019-nCoV not detected	Not Detected	Report results to sender. Consider testing for other respiratory viruses.[b]
-	-	-	Invalid Result	Invalid	Repeat extraction and rRT-PCR. If the repeated result remains invalid, consider collecting a new specimen from the patient.

[a]Laboratories should report their diagnostic result as appropriate and in compliance with their specific reporting system.

[b]Optimum specimen types and timing for peak viral levels during infections caused by 2019-nCoV have not been determined. Collection of multiple specimens from the same patient may be necessary to detect the virus. The possibility of a false negative result should especially be considered if the patient's recent exposures or clinical presentation suggest that 2019-nCoV infection is possible, and diagnostic tests for other causes of illness (e.g., other respiratory illness) are negative. If 2019-nCoV infection is still suspected, re-testing should be considered in consultation with public health authorities.

Quality Control

- Quality control requirements must be performed in conformance with local, state, and federal regulations or accreditation requirements and the user's laboratory's standard quality control procedures. For further guidance on appropriate quality control practices, refer to 42 CFR 493.1256.
- Quality control procedures are intended to monitor reagent and assay performance.
- Test all positive controls prior to running diagnostic samples with each new kit lot to ensure all reagents and kit components are working properly.
- Good laboratory practice (cGLP) recommends including a positive extraction control in each nucleic acid isolation batch.
- Although HSC is not included with the 2019-nCov rRT-PCR Diagnostic Panel, the HSC extraction control must proceed through nucleic acid isolation per batch of specimens to be tested.
- Always include a negative control (NTC), and the appropriate positive control (nCoVPC) in each amplification and detection run. All clinical samples should be tested for human RNAse P gene to control for specimen quality and extraction.

Limitations

- All users, analysts, and any person reporting diagnostic results should be trained to perform this procedure by a competent instructor. They should demonstrate their ability to perform the test and interpret the results prior to performing the assay independently.
- Performance of the CDC 2019-nCoV Real-Time RT-PCR Diagnostic Panel has only been established in upper and lower respiratory specimens (such as nasopharyngeal or oropharyngeal swabs, sputum, lower respiratory tract aspirates, bronchoalveolar lavage, and nasopharyngeal wash/aspirate or nasal aspirate).
- Negative results do not preclude 2019-nCoV infection and should not be used as the sole basis for treatment or other patient management decisions. Optimum specimen types and timing for peak viral levels during infections caused by 2019-nCoV have not been determined. Collection of multiple specimens (types and time points) from the same patient may be necessary to detect the virus.
- A false negative result may occur if a specimen is improperly collected, transported or handled. False negative results may also occur if amplification inhibitors are present in the specimen or if inadequate numbers of organisms are present in the specimen.
- Positive and negative predictive values are highly dependent on prevalence. False negative test results are more likely when prevalence of disease is high. False positive test results are more likely when prevalence is moderate to low.
- Do not use any reagent past the expiration date.
- If the virus mutates in the rRT-PCR target region, 2019-nCoV may not be detected or may be detected less predictably. Inhibitors or other types of interference may produce a false negative result. An interference study evaluating the effect of common cold medications was not performed.
- Test performance can be affected because the epidemiology and clinical spectrum of infection caused by 2019-nCoV is not fully known. For example, clinicians and laboratories may not know the optimum types of specimens to collect, and, during the course of infection, when these specimens are most likely to contain levels of viral RNA that can be readily detected.
- Detection of viral RNA may not indicate the presence of infectious virus or that 2019-nCoV is the causative agent for clinical symptoms.

- The performance of this test has not been established for monitoring treatment of 2019-nCoV infection.
- The performance of this test has not been established for screening of blood or blood products for the presence of 2019-nCoV.
- This test cannot rule out diseases caused by other bacterial or viral pathogens.

Conditions of Authorization for the Laboratory

The CDC 2019-nCoV Real-Time RT-PCR Diagnostic Panel Letter of Authorization, along with the authorized Fact Sheet for Healthcare Providers, the authorized Fact Sheet for Patients and authorized labeling are available on the FDA website:
https://www.fda.gov/MedicalDevices/Safety/EmergencySituations/ucm161496.htm
Use of the CDC 2019-nCoV Real-Time RT-PCR Diagnostic Panel must follow the procedures outlined in these manufacturer's Instructions for Use and the conditions of authorization outlined in the Letter of Authorization. Deviations from the procedures outlined are not permitted under the Emergency Use Authorization (EUA). To assist clinical laboratories running the CDC 2019-nCoV Real-Time RT-PCR Diagnostic Panel, the relevant Conditions of Authorization are listed verbatim below, and are required to be met by laboratories performing the EUA test.

- Authorized laboratories[1] will include with reports of the results of the CDC 2019-nCoV Real-Time RT-PCR Diagnostic Panel, all authorized Fact Sheets. Under exigent circumstances, other appropriate methods for disseminating these Fact Sheets may be used, which may include mass media.

- Authorized laboratories will perform the CDC 2019-nCoV Real-Time RT-PCR Diagnostic Panel as outlined in the CDC 2019-Novel Coronavirus (2019-nCoV) Real-Time RT-PCR Diagnostic Panel Instructions for Use. Deviations from the authorized procedures, including the authorized RT-PCR instruments, authorized extraction methods, authorized clinical specimen types, authorized control materials, authorized other ancillary reagents and authorized materials required to perform the CDC 2019-nCoV Real-Time RT-PCR Diagnostic Panel are not permitted. [2]

- Authorized laboratories that receive the CDC 2019-nCoV Real-Time RT-PCR Diagnostic Panel must notify the relevant public health authorities of their intent to run the test prior to initiating testing.

- Authorized laboratories will have a process in place for reporting test results to healthcare providers and relevant public health authorities, as appropriate.

- Authorized laboratories will collect information on the performance of the test and report to DMD/OHT7-OIR/OPEQ/CDRH (via email: CDRH-EUA-Reporting@fda.hhs.gov) and CDC

[1]Authorized Laboratories: For ease of reference, the Letter of Authorization refers to "laboratories certified under the Clinical Laboratory Improvement Amendments of 1988 (CLIA), 42 U.S.C. § 263a, to perform high complexity tests" as "authorized laboratories."

[2]If an authorized laboratory is interested in implementing changes to the CDC 2019-nCoV Real-Time RT-PCR Diagnostic Panel that are not in the scope (Section II) of this letter of authorization FDA recommends you discuss with FDA after considering the policy outlined in *Immediately in Effect Guidance for Clinical Laboratories and Food and Drug Administration Staff: Policy for Diagnostics Testing in Laboratories Certified to Perform High Complexity Testing under CLIA prior to Emergency Use Authorization for Coronavirus Disease-2019 during the Public Health Emergency* (https://www.fda.gov/media/135659/download).

(respvirus@cdc.gov) any suspected occurrence of false positive or false negative results and significant deviations from the established performance characteristics of the test of which they become aware.

- Authorized laboratories will report adverse events, including problems with test performance or results, to MedWatch by submitting the online FDA Form 3500 (https://www.accessdata.fda.gov/scripts/medwatch/index.cfm?action=reporting.home) or by calling 1-800-FDA-1088

- All laboratory personnel using the test must be appropriately trained in RT-PCR techniques and use appropriate laboratory and personal protective equipment when handling this kit and use the test in accordance with the authorized labeling.

- CDC, IRR, manufacturers and distributors of commercial materials identified as acceptable on the CDC website, and authorized laboratories will ensure that any records associated with this EUA are maintained until otherwise notified by FDA. Such records will be made available to FDA for inspection upon request.

Performance Characteristics

Analytical Performance:

Limit of Detection (LoD):

LoD studies determine the lowest detectable concentration of 2019-nCoV at which approximately 95% of all (true positive) replicates test positive. The LoD was determined by limiting dilution studies using characterized samples.

The analytical sensitivity of the rRT-PCR assays contained in the CDC 2019 Novel Coronavirus (2019-nCoV) Real-Time RT-PCR Diagnostic Panel were determined in Limit of Detection studies. Since no quantified virus isolates of the 2019-nCoV are currently available, assays designed for detection of the 2019-nCoV RNA were tested with characterized stocks of in vitro transcribed full length RNA (N gene; GenBank accession: MN908947.2) of known titer (RNA copies/µL) spiked into a diluent consisting of a suspension of human A549 cells and viral transport medium (VTM) to mimic clinical specimen. Samples were extracted using the QIAGEN EZ1 Advanced XL instrument and EZ1 DSP Virus Kit (Cat# 62724) and manually with the QIAGEN DSP Viral RNA Mini Kit (Cat# 61904). Real-Time RT-PCR assays were performed using the ThemoFisher Scientific TaqPath™ 1-Step RT-qPCR Master Mix, CG (Cat# A15299) on the Applied Biosystems™ 7500 Fast Dx Real-Time PCR Instrument according to the CDC 2019-nCoV Real-Time RT-PCR Diagnostic Panel instructions for use.

A preliminary LoD for each assay was determined testing triplicate samples of RNA purified using each extraction method. The approximate LoD was identified by extracting and testing 10-fold serial dilutions of characterized stocks of in vitro transcribed full-length RNA. A confirmation of the LoD was determined using 3-fold serial dilution RNA samples with 20 extracted replicates. The LoD was determined as the lowest concentration where ≥ 95% (19/20) of the replicates were positive.

Table 4. Limit of Detection Confirmation of the CDC 2019-nCoV Real-Time RT-PCR Diagnostic Panel with QIAGEN EZ1 DSP

Targets	2019-nCoV_N1			2019-nCoV_N2		
RNA Concentration[1]	$10^{0.5}$	$10^{0.0}$	$10^{-0.5}$	$10^{0.5}$	$10^{0.0}$	$10^{-0.5}$
Positives/Total	20/20	19/20	13/20	20/20	17/20	9/20
Mean Ct[2]	32.5	35.4	NA	35.8	NA	NA
Standard Deviation (Ct)	0.5	0.8	NA	1.3	NA	NA

[1] Concentration is presented in RNA copies/µL

[2] Mean Ct reported for dilutions that are ≥ 95% positive. Calculations only include positive results.

NA not applicable

Table 5. Limit of Detection Confirmation CDC 2019-nCoV Real-Time RT-PCR Diagnostic Panel with QIAGEN QIAmp DSP Viral RNA Mini Kit

Targets	2019-nCoV_N1			2019-nCoV_N2			
RNA Concentration[1]	$10^{0.5}$	$10^{0.0}$	$10^{-0.5}$	$10^{0.5}$	$10^{0.0}$	$10^{-0.5}$	$10^{-1.0}$
Positives/Total	20/20	20/20	6/20	20/20	20/20	20/20	8/20
Mean Ct[2]	32.0	32.8	NA	33.0	35.4	36.2	NA
Standard Deviation (Ct)	0.7	0.8	NA	1.4	0.9	1.9	NA

[1] Concentration is presented in RNA copies/µL

[2] Mean Ct reported for dilutions that are ≥ 95% positive. Calculations only include positive results.

NA not applicable

Table 6. Limit of Detection of the CDC 2019-nCoV Real-Time RT-PCR Diagnostic Panel

**Virus**	**Material**	**Limit of Detection (RNA copies/µL)**	
		**QIAGEN EZ1 Advanced XL**	**QIAGEN DSP Viral RNA Mini Kit**
2019 Novel Coronavirus	N Gene RNA Transcript	$10^{0.5}$	10^{0}

FDA Sensitivity Evaluation: The analytical sensitivity of the test will be further assessed by evaluating an FDA-recommended reference material using an FDA developed protocol if applicable and/or when available.

In Silico Analysis of Primer and Probe Sequences:

An alignment was performed with the oligonucleotide primer and probe sequences of the CDC 2019 nCoV Real-Time RT-PCR Diagnostic Panel with all publicly available nucleic acid sequences for 2019-nCoV in GenBank as of February 1, 2020 to demonstrate the predicted inclusivity of the CDC 2019 nCoV Real-Time RT-PCR Diagnostic panel. All the alignments show 100% identity of the CDC panel to the available 2019-nCoV sequences with the exception of one nucleotide mismatch with the N1 forward primer in one deposited sequence. The risk of a single mismatch resulting in a significant loss in reactivity, and false negative result, is

low due to the design of the primers and probes with melting temperatures > 60°C and run conditions of the assay with annealing temperature at 55°C to tolerate one to two mismatches.

Specificity/Exclusivity Testing: In Silico Analysis

BLASTn analysis queries of the 2019-nCoV rRT-PCR assays primers and probes were performed against public domain nucleotide sequences. The database search parameters were as follows: 1) The nucleotide collection consists of GenBank+EMBL+DDBJ+PDB+RefSeq sequences, but excludes EST, STS, GSS, WGS, TSA, patent sequences as well as phase 0, 1, and 2 HTGS sequences and sequences longer than 100Mb; 2) The database is non-redundant. Identical sequences have been merged into one entry, while preserving the accession, GI, title and taxonomy information for each entry; 3) Database was updated on 10/03/2019; 4) The search parameters automatically adjust for short input sequences and the expect threshold is 1000; 5) The match and mismatch scores are 1 and -3, respectively; 6) The penalty to create and extend a gap in an alignment is 5 and 2 respectively.

2019-nCoV_N1 Assay:
Probe sequence of 2019-nCoV rRT-PCR assay N1 showed high sequence homology with SARS coronavirus and Bat SARS-like coronavirus genome. However, forward and reverse primers showed no sequence homology with SARS coronavirus and Bat SARS-like coronavirus genome. Combining primers and probe, there is no significant homologies with human genome, other coronaviruses or human microflora that would predict potential false positive rRT-PCR results.

2019-nCoV_N2 Assay:
The forward primer sequence of 2019-nCoV rRT-PCR assay N2 showed high sequence homology to Bat SARS-like coronaviruses. The reverse primer and probe sequences showed no significant homology with human genome, other coronaviruses or human microflora. Combining primers and probe, there is no prediction of potential false positive rRT-PCR results.

In summary, the 2019-nCoV rRT-PCR assay N1 and N2, designed for the specific detection of 2019-nCoV, showed no significant combined homologies with human genome, other coronaviruses, or human microflora that would predict potential false positive rRT-PCR results.

In addition to the *in silico* analysis, several organisms were extracted and tested with the CDC 2019-nCoV Real-Time RT-PCR Diagnostic Panel to demonstrate analytical specificity and exclusivity. Studies were performed with nucleic acids extracted using the QIAGEN EZ1 Advanced XL instrument and EZ1 DSP Virus Kit. Nucleic acids were extracted from high titer preparations (typically ≥ 10^5 PFU/mL or ≥ 10^6 CFU/mL). Testing was performed using the ThemoFisher Scientific TaqPath™ 1-Step RT-qPCR Master Mix, CG on the Applied Biosystems™ 7500 Fast Dx Real-Time PCR instrument. The data demonstrate that the expected results are obtained for each organism when tested with the CDC 2019-nCoV Real-Time RT-PCR Diagnostic Panel.

Table 7. Specificity/Exclusivity of the CDC 2019-nCoV Real-Time RT-PCR Diagnostic Panel

Virus	Strain	Source	2019-nCoV_N1	2019-nCoV_N2	Final Result
Human coronavirus	229E	isolate	0/3	0/3	Neg.
Human coronavirus	OC43	isolate	0/3	0/3	Neg.
Human coronavirus	NL63	clinical specimen	0/3	0/3	Neg.
Human coronavirus	HKU1	clinical specimen	0/3	0/3	Neg.
MERS-coronavirus		isolate	0/3	0/3	Neg.
SARS-coronavirus		isolate	0/3	0/3	Neg.
bocavirus	-	clinical specimen	0/3	0/3	Neg.
Mycoplasma pneumoniae		isolate	0/3	0/3	Neg.
Streptococcus		isolate	0/3	0/3	Neg.
Influenza A(H1N1)		isolate	0/3	0/3	Neg.
Influenza A(H3N2)		isolate	0/3	0/3	Neg.
Influenza B		isolate	0/3	0/3	Neg.
Human adenovirus, type 1	Ad71	isolate	0/3	0/3	Neg.
Human metapneumovirus	-	isolate	0/3	0/3	Neg.
respiratory syncytial virus	Long A	isolate	0/3	0/3	Neg.
rhinovirus		isolate	0/3	0/3	Neg.
parainfluenza 1	C35	isolate	0/3	0/3	Neg.
parainfluenza 2	Greer	isolate	0/3	0/3	Neg.
parainfluenza 3	C-43	isolate	0/3	0/3	Neg.
parainfluenza 4	M-25	isolate	0/3	0/3	Neg.

Endogenous Interference Substances Studies:

The CDC 2019-nCoV Real-Time RT-PCR Diagnostic Panel uses conventional well-established nucleic acid extraction methods and based on our experience with CDC's other EUA assays, including the CDC Novel Coronavirus 2012 Real-time RT-PCR Assay for the presumptive detection of Middle East Respiratory Syndrome Coronavirus (MERS-CoV) and the CDC Human Influenza Virus Real-Time RT-PCR Diagnostic Panel-Influenza A/H7 (Eurasian Lineage) Assay for the presumptive detection of novel influenza A (H7N9) virus that are both intended for use with a number of respiratory specimens, we do not anticipate interference from common endogenous substances.

Specimen Stability and Fresh-frozen Testing:

To increase the likelihood of detecting infection, CDC recommends collection of lower respiratory and upper respiratory specimens for testing. If possible, additional specimen types (e.g., stool, urine) should be collected and should be stored initially until decision is made by CDC whether additional specimen sources should be tested. Specimens should be collected as soon as possible once a PUI is identified regardless of symptom onset. Maintain proper infection control when collecting specimens. Store specimens at 2-8°C and ship overnight to CDC on ice pack. Label each specimen container with the patient's ID number (e.g., medical record number), unique specimen ID (e.g., laboratory requisition number), specimen type (e.g., nasal swabs) and the date the sample was collected. Complete a CDC Form 50.34 for each specimen submitted.

Clinical Performance:

As of February 22, 2020, CDC has tested 2071 respiratory specimens from persons under investigation (PUI) in the U.S. using the CDC 2019-nCoV Real-Time RT-PCR Diagnostic Panel. Specimen types include bronchial fluid/wash, buccal swab, nasal wash/aspirate, nasopharyngeal swab, nasopharyngeal/throat swab, oral swab, sputum, oropharyngeal (throat) swab, swab (unspecified), and throat swab.

Table 8: Summary of CDC 2019-Novel Coronavirus (2019-nCoV) Real-Time RT-PCR Diagnostic Panel Data Generated by Testing Human Respiratory Specimens Collected from PUI Subjects in the U.S.

Specimen Type	2019 nCoV Negative	2019 nCoV Positive	Inconclusive	Invalid	Total
**Bronchial fluid/wash**	2	0	0	0	2
**Buccal swab**	5	1	0	0	6
**Nasal wash/aspirate**	6	0	0	0	6
**Nasopharyngeal swab**	927	23	0	0	950
**Nasopharyngeal swab/throat swab**	4	0	0	0	4
**Oral swab**	476	9	0	0	485
**Pharyngeal (throat) swab**	363	10	0	1	374
**Sputum**	165	5	0	0	170
**Swab (unspecified)**[1]	71	1	0	0	72
**Tissue (lung)**	2	0	0	0	2
**Total**	2021	49	0	1	2071

[1]Actual swab type information was missing from these upper respiratory tract specimens.

Two thousand twenty-one (2021) respiratory specimens of the 2071 respiratory specimens tested negative by the CDC 2019-nCoV Real-Time RT-PCR Diagnostic Panel. Forty-nine (49) of the 2071 respiratory specimens tested positive by the CDC 2019-nCoV Real-Time RT-PCR Diagnostic Panel. Only one specimen (oropharyngeal (throat) swab) was invalid. Of the 49 respiratory specimens that tested positive by the CDC 2019-nCoV Real-Time RT-PCR Diagnostic Panel, seventeen (17) were confirmed by genetic sequencing and/or virus culture (positive percent agreement = 17/17, 95% CI: 81.6%-100%)

During the early phase of the testing, a total of 117 respiratory specimens collected from 46 PUI subjects were also tested with two analytically validated real-time RT-PCR assays that target separate and independent regions of the nucleocapsid protein gene of the 2019-nCoV, N4 and N5 assays. The nucleocapsid protein gene targets for the N4 and N5 assays are different and independent from the nucleocapsid protein gene targets for the two RT-PCR assays included in the CDC 2019-nCoV Real-Time RT-

PCR Diagnostic Panel, N1 and N2. Any positive result from the N4 and/or the N5 assay was further investigated by genetic sequencing.

Performance of the CDC 2019-nCoV Real-Time RT-PCR Diagnostic Panel testing these 117 respiratory specimens was estimated against a composite comparator. A specimen was considered comparator negative if both the N4 and the N5 assays were negative. A specimen was considered comparator positive when the N4 and/or the N5 assay generated a positive result, and the comparator positive result(s) were further investigated and confirmed to be 2019-nCoV RNA positive by genetic sequencing.

Table 9: Percent Agreement of the CDC 2019-nCoV Real-Time RT-PCR Diagnostic Panel with the Composite Comparator

**CDC 2019-nCoV Panel Result**	**Composite Comparator Result**	
	Positive	Negative
Positive	13[1]	0
Inconclusive	0	0
Negative	0	104

[1]Composite comparator results were available for 13 of 49 CDC 2019-nCoV Panel positive specimens only.

Positive percent agreement = 13/13 = 100% (95% CI: 77.2% - 100%)
Negative percent agreement = 104/104 = 100% (95% CI: 96.4% - 100%)

Enzyme Master Mix Evaluation:

The limit of detection equivalence between the ThermoFisher TaqPath™ 1-Step RT-qPCR Master Mix and the following enzyme master mixes was evaluated: Quantabio qScript XLT One-Step RT-qPCR ToughMix, Quantabio UltraPlex 1-Step ToughMix (4X), and Promega GoTaq® Probe 1- Step RT-qPCR System. Serial dilutions of 2019 novel coronavirus (SARS CoV-2) transcript were tested in triplicate with the CDC 2019-nCoV Real-time RT-PCR Diagnostic Panel using all four enzyme master mixes. Both manufactured versions of oligonucleotide probe, BHQ and ZEN, were used in the comparison. The lowest detectable concentration of transcript at which all replicates tested positive using the Quantabio qScript XLT One-Step RT-qPCR ToughMix and Quantabio UltraPlex 1-Step ToughMix (4X) was similar to that observed for the ThemoFisher TaqPath™ 1-Step RT-qPCR Master Mix. The lowest detectable concentration of transcript when using the Promega GoTaq® Probe 1- Step RT-qPCR System was one dilution above that observed for the other candidates when evaluated with the BHQ version of the CDC assays. The candidate master mixes all performed equivalently or at one dilution below the ThemoFisher TaqPath™ 1-Step RT-qPCR Master Mix when evaluated with the ZEN version of the CDC assays.

Table 10: Limit of Detection Comparison for Enzyme Master Mixes – BHQ Probe Summary Results

Copy Number	ThemoFisher TaqPath™ 1-Step RT-qPCR Master Mix		Quantabio qScript XLT One-Step RT-qPCR ToughMix		Quantabio UltraPlex 1-Step ToughMix (4X)		Promega GoTaq® Probe 1- Step RT-qPCR System	
	2019-nCoV_N1	2019-nCoV_N2	2019-nCoV_N1	2019-nCoV_N2	2019-nCoV_N1	2019-nCoV_N2	2019-nCoV_N1	2019-nCoV_N2
10^2 copies/µL	3/3	3/3	3/3	3/3	3/3	3/3	3/3	3/3
10^1 copies/µL	3/3	3/3	3/3	3/3	3/3	3/3	3/3	3/3
10^0 copies/µL	3/3	3/3	3/3	3/3	3/3	3/3	3/3	2/3
10^{-1} copies µL	2/3	0/3	1/3	1/3	1/3	1/3	0/3	0/3

Table 11: Limit of Detection Comparison for Enzyme Master Mixes – ZEN Probe Summary Results

Copy Number	ThemoFisher TaqPath™ 1-Step RT-qPCR Master Mix		Quantabio qScript XLT One-Step RT-qPCR ToughMix		Quantabio UltraPlex 1-Step ToughMix (4X)		Promega GoTaq® Probe 1- Step RT-qPCR System	
	2019-nCoV_N1	2019-nCoV_N2	2019-nCoV_N1	2019-nCoV_N2	2019-nCoV_N1	2019-nCoV_N2	2019-nCoV_N1	2019-nCoV_N2
10^2 copies/µL	3/3	3/3	3/3	3/3	3/3	3/3	3/3	3/3
10^1 copies/µL	3/3	3/3	3/3	3/3	3/3	3/3	3/3	3/3
10^0 copies/µL	3/3	2/3	3/3	3/3	3/3	2/3	3/3	3/3
10^{-1} copies µL	1/3	1/3	0/3	0/3	0/3	1/3	1/3	1/3

Retrospective positive (18) and negative (17) clinical respiratory specimens were extracted using the QIAGEN EZ1 Advanced XL instrument and EZ1 DSP Virus Kit and were tested with the CDC 2019-nCoV Real-time RT-PCR Diagnostic Panel using the Quantabio qScript XLT One-Step RT-qPCR ToughMix, Quantabio UltraPlex 1-Step ToughMix (4X), and Promega GoTaq® Probe 1- Step RT-qPCR System master mixes. All three enzyme master mixes performed equivalently, demonstrating 100% positive and 100% negative agreement with expected results and a 95% confidence interval of 82.4%-100% and 81.6%-100%, respectively.

Table 12: Clinical Comparison – Retrospective Study Summary Results

CDC 2019-nCoV Real-time RT-PCR Diagnostic Panel Result	Quantabio qScript XLT One-Step RT-qPCR ToughMix		Quantabio UltraPlex 1-Step ToughMix (4X)		Promega GoTaq® Probe 1-Step RT-qPCR System	
	Positive	Negative	Positive	Negative	Positive	Negative
Positive	18	0	18	0	18	0
Negative	0	17	0	17	0	17

Disposal

Dispose of hazardous or biologically contaminated materials according to the practices of your institution.

References

1. Ballew, H. C., *et al*. "Basic Laboratory Methods in Virology," DHHS, Public Health Service 1975 (Revised 1981), Centers for Disease Control and Prevention, Atlanta, Georgia 30333.

2. Clinical Laboratory Standards Institute (CLSI), "Collection, Transport, Preparation and Storage of Specimens for Molecular Methods: Proposed Guideline," MM13-A

3. Lieber, M., *et al*. "A Continuous Tumor Cell Line from a Human Lung Carcinoma with Properties of Type II Alveolar Epithelial Cells." *International Journal of Cancer* 1976, 17(1), 62-70.

Revision History

Revision #	Effective Date	Summary of Revisions
1	February 4, 2020	Original Instructions for Use
2	March 15, 2020	• Intended use update<br>• Removal of N3 primer and probe set from Diagnostic Panel<br>• Performance data update<br>• Addition of alternative nucleic acid extraction platforms<br>• Addition of acceptable alternatives to HSC and addition of QIAGEN RUO extraction reagents<br>• Positive results no longer presumptive. No confirmation of positive results required
3	March 30, 2020	• Addition of alternative enzyme master mix options

Contact Information, Ordering, and Product Support

For technical and product support, contact the CDC Division of Viral Diseases directly.

Send email to: respvirus@cdc.gov

Note: If your laboratory is using reagents sourced from someone other than the CDC International Reagent Resource, please refer to the manufacturer's instructions provided with the commercial materials.

DO NOT DISCARD: Important product-specific information

CDC 2019-nCoV Real-Time RT-PCR Diagnostic Panel
For use under EMERGENCY USE AUTHORIZATION (EUA) only.
Rx only

CATALOG: 2019-nCoV EUA-01
KIT LOT:
EXPIRATION DATE: YYYY-MM-DD (3 Years from DOM)

INTENDED USE
The CDC 2019-Novel Coronavirus (2019-nCoV) Real-Time RT-PCR Diagnostic Panel is a real-time RT-PCR test intended for the qualitative detection of nucleic acid from the 2019-nCoV in upper and lower respiratory specimens (such as nasopharyngeal or oropharyngeal swabs, sputum, lower respiratory tract aspirates, bronchoalveolar lavage, and nasopharyngeal wash/aspirate or nasal aspirate) collected from individuals who meet 2019-nCoV clinical and/or epidemiological criteria (for example, clinical signs and symptoms associated with 2019-nCoV infection, contact with a probable or confirmed 2019-nCoV case, history of travel to a geographic locations where 2019-nCoV cases were detected, or other epidemiologic links for which 2019-nCoV testing may be indicated as part of a public health investigation). Testing in the United States is limited to laboratories certified under the Clinical Laboratory Improvement Amendments of 1988 (CLIA), 42 U.S.C. § 263a, to perform high complexity tests.

Results are for the identification of 2019-nCoV RNA. The 2019-nCoV RNA is generally detectable in upper and lower respiratory specimens during infection. Positive results are indicative of active infection with 2019-nCoV but do not rule out bacterial infection or co-infection with other viruses. The agent detected may not be the definite cause of disease. Laboratories within the United States and its territories are required to report all positive results to the appropriate public health authorities.

Negative results do not preclude 2019-nCoV infection and should not be used as the sole basis for treatment or other patient management decisions. Negative results must be combined with clinical observations, patient history, and epidemiological information.

Testing with the CDC 2019-nCoV Real-Time RT-PCR Diagnostic Panel is intended for use by trained laboratory personnel who are proficient in performing real-time RT-PCR assays. The CDC 2019-Novel Coronavirus (2019-nCoV) Real-Time RT-PCR Diagnostic Panel is only for use under a Food and Drug Administration's Emergency Use Authorization .

PACKAGE CONTENTS

PACKAGING	COMPONENT	PART NUMBER	COMPONENT LOT NUMBER	VIALS PER KIT	QUANTITY /VIAL	STATE
Oligonucleotide Box	2019-nCoV_N1 Combined Primer/Probe Mix	RV202001		1	22.5 nmol	Dried
	2019-nCoV_N2 Combined Primer/Probe Mix	RV202002		1	22.5 nmol	Dried
	RP Combined Primer/Probe Mix	RV202004		1	22.5 nmol	Dried
Control Box	nCoVPC 2019-nCoV Positive Control (non-infectious)	RV202005		4	1×10^4 copies/µL	Dried

STORAGE INSTRUCTIONS
Upon receipt, store at 2-8°C. Refer to the CDC 2019-Novel Coronavirus (2019-nCoV) Real-Time RT-PCR Diagnostic Panel Instructions for Use before opening and preparing reagents for use.

PROCEDURE/INTERPRETATION/LIMITATIONS
Users should refer to the **CDC 2019-Novel Coronavirus (2019-nCoV) Real-Time RT-PCR Diagnostic Panel Instructions for Use** posted on the FDA website for all IVD products used under Emergency Use Authorization, http://www.fda.gov/MedicalDevices/Safety/EmergencySituations/ucm161496.htm.

IVD

PRECAUTIONS

This reagent should be handled in an approved BSL-2 handling area to avoid contamination of laboratory equipment and reagents that could cause false positive results. This product is non-infectious. However, this product should be handled in accordance with Good Laboratory Practices.

REAGENT COMPLAINTS/QUESTIONS

If you have a question/comment about this product, please contact the CDC Division of Viral Diseases/Respiratory Viruses Branch by email at respvirus@cdc.gov.

DISTRIBUTED BY

Manufactured by the Centers for Disease Control and Prevention, 1600 Clifton Road, Atlanta, Georgia, 30329, USA

IVD

****** DO NOT DISCARD: Important product-specific information ******

CDC 2019-nCoV Real-Time RT-PCR Diagnostic Panel – Verification Requirements

Please consult the following guidance from CMS regarding Emergency Use Authorized diagnostic tests: https://www.cms.gov/Medicare/Provider-Enrollment-and-Certification/SurveyCertificationGenInfo/Policy-and-Memos-to-States-and-Regions-Items/QSO18-19-CLIA

INTENDED USE

The CDC 2019-Novel Coronavirus (2019-nCoV) Real-Time RT-PCR Diagnostic Panel is a real-time RT-PCR test intended for the qualitative detection of nucleic acid from the 2019-nCoV in upper and lower respiratory specimens (such as nasopharyngeal or oropharyngeal swabs, sputum, lower respiratory tract aspirates, bronchoalveolar lavage, and nasopharyngeal wash/aspirate or nasal aspirate) collected from individuals who meet 2019-nCoV clinical and/or epidemiological criteria (for example, clinical signs and symptoms associated with 2019-nCoV infection, contact with a probable or confirmed 2019-nCoV case, history of travel to a geographic locations where 2019-nCoV cases were detected, or other epidemiologic links for which 2019-nCoV testing may be indicated as part of a public health investigation). Testing in the United States is limited to laboratories certified under the Clinical Laboratory Improvement Amendments of 1988 (CLIA), 42 U.S.C. § 263a, to perform high complexity tests.

Results are for the identification of 2019-nCoV RNA. The 2019-nCoV RNA is generally detectable in upper and lower respiratory specimens during infection. Positive results are indicative of active infection with 2019-nCoV but do not rule out bacterial infection or co-infection with other viruses. The agent detected may not be the definite cause of disease. Laboratories within the United States and its territories are required to report all positive results to the appropriate public health authorities.

Negative results do not preclude 2019-nCoV infection and should not be used as the sole basis for treatment or other patient management decisions. Negative results must be combined with clinical observations, patient history, and epidemiological information.

Testing with the CDC 2019-nCoV Real-Time RT-PCR Diagnostic Panel is intended for use by trained laboratory personnel who are proficient in performing real-time RT-PCR assays. The CDC 2019-Novel Coronavirus (2019-nCoV) Real-Time RT-PCR Diagnostic Panel is only for use under a Food and Drug Administration’s Emergency Use Authorization.

REQUIRED MATERIALS

The 2019 novel coronavirus positive control (nCoVPC) is provided with the CDC 2019-nCoV Real-Time RT-PCR Diagnostic Panel and should be prepared according to the instructions for use. The nCoVPC consists of an RNA transcript of the 2019-nCoV N gene as well as human RNase P gene segment. nCoVPC will yield a positive result with the following primer and probe sets: 2019-nCoV_N1, 2019-nCoV_N2, and RP.

Approximately 2 mL of an upper respiratory specimen (e.g. nasopharyngeal swabs (NPS) in transport media) will be needed for testing. Specimens may be pooled if less than 2mL of one specimen is available.

Refer to CDC 2019-nCoV Real-Time RT-PCR Diagnostic Panel package insert (manufacturer instructions) for additional reagents, materials, and instructions.

PRECAUTIONS

This reagent should be handled in an approved BSL-2 handling area to avoid contamination of laboratory equipment and reagents that could cause false positive results. This product is an

****** DO NOT DISCARD: Important product-specific information ******

RNA transcript and is non-infectious. However, the nCoVPC should be handled in accordance with Good Laboratory Practices.

Store reagent at appropriate temperatures (see instructions for use) and hold on ice when thawed.

Please use standard precautions when handling respiratory specimens.

INSTRUCTIONS FOR PREPARING SAMPLES BEFORE EXTRACTION WITH THE QIAamp DSP VIRAL RNA MINI KIT OR THE QIAamp VIRAL RNA MINI KIT

- Refer to the 2019-nCoV Real-Time RT-PCR Diagnostic Panel instructions for use for reconstitution of the materials for use. RNA should be kept cold during preparation and use.
- Make a 1/10 dilution of nCoVPC by adding 5 μL of nCoVPC into 45 μL of nuclease-free water or 10 mM Tris
- Aliquot 560 μL of lysis buffer into each of nine tubes labeled 1-9.
- Add 140 μL of upper respiratory specimen (e.g. NPS in viral transport media) into each of the nine labeled tubes with lysis buffer
- To prepare samples at a moderate concentration, spike 14 μL of undiluted nCoVPC (rehydrated as described in the CDC 2019-nCoV Real-Time RT-PCR Diagnostic Panel instructions for use) into each tube labeled 1-3 containing lysis buffer and specimen
- To prepare samples at a low concentration, spike 14 μL of 1/10 dilution of nCoVPC into each tube labeled 4-6 containing lysis buffer and specimen
- To prepare negative samples, spike 14 μL of nuclease-free water into each tube labeled 7-9 containing lysis buffer and specimen
- Perform extractions of all nine samples according to the CDC 2019-nCoV Real-Time RT-PCR Diagnostic Panel instructions for use

INSTRUCTIONS FOR PREPARING SAMPLES BEFORE EXTRACTION WITH THE QIAGEN EZ1 ADVANCED XL

- Refer to the 2019-nCoV Real-Time RT-PCR Diagnostic Panel instructions for use for reconstitution of the materials for use. RNA should be kept cold during preparation and use.
- Make a 1/10 dilution of nCoVPC by adding 5 μL of nCoVPC into 45 μL of nuclease-free water or 10 mM Tris
- Aliquot 280 μL of lysis buffer into each of nine tubes labeled 1-9.
- Add 120 μL of upper respiratory specimen (e.g. NPS in viral transport media) into each of the nine labeled tubes with lysis buffer
- To prepare samples at a moderate concentration, spike 12 μL of undiluted nCoVPC (rehydrated as described in the CDC 2019-nCoV Real-Time RT-PCR Diagnostic Panel instructions for use) into each tube labeled 1-3 containing lysis buffer and specimen
- To prepare samples at a low concentration, spike 12 μL of 1/10 dilution of nCoVPC into each tube labeled 4-6 containing lysis buffer and specimen
- To prepare negative samples, spike 12 μL of nuclease-free water into each tube labeled 7-9 containing lysis buffer and specimen
- Perform extractions of all nine samples according to the CDC 2019-nCoV Real-Time RT-PCR Diagnostic Panel instructions for use

INSTRUCTIONS FOR PREPARING SAMPLES BEFORE EXTRACTION WITH THE ROCHE MagNA PURE TOTAL NUCLEIC ACID KIT OR THE ROCHE MagNA PURE NUCLEIC ACID ISOLATION KIT I

- Refer to the 2019-nCoV Real-Time RT-PCR Diagnostic Panel instructions for use for reconstitution of the materials for use. RNA should be kept cold during preparation and use.
- Make a 1/10 dilution of nCoVPC by adding 5 μL of nCoVPC into 45 μL of nuclease-free water or 10 mM Tris
- Aliquot 300 μL of lysis buffer into each of nine tubes labeled 1-9.
- Add 100 μL of upper respiratory specimen (e.g. NPS in viral transport media) into each of the nine labeled tubes with lysis buffer

****** DO NOT DISCARD: Important product-specific information ******

- To prepare samples at a moderate concentration, spike 12 μL of undiluted nCoVPC (rehydrated as described in the CDC 2019-nCoV Real-Time RT-PCR Diagnostic Panel instructions for use) into each tube labeled 1-3 containing lysis buffer and specimen
- To prepare samples at a low concentration, spike 12 μL of 1/10 dilution of nCoVPC into each tube labeled 4-6 containing lysis buffer and specimen
- To prepare negative samples, spike 12 μL of nuclease-free water into each tube labeled 7-9 containing lysis buffer and specimen
- Perform extractions of all nine samples according to the CDC 2019-nCoV Real-Time RT-PCR Diagnostic Panel instructions for use

INSTRUCTIONS FOR PREPARING SAMPLES BEFORE EXTRACTION WITH THE ROCHE MagNA PURE 96 DNA AND VIRAL NA SMALL VOLUME KIT

- Refer to the 2019-nCoV Real-Time RT-PCR Diagnostic Panel instructions for use for reconstitution of the materials for use. RNA should be kept cold during preparation and use.
- Make a 1/10 dilution of nCoVPC by adding 5 μL of nCoVPC into 45 μL of nuclease-free water or 10 mM Tris
- Aliquot 350 μL of lysis buffer into each of nine tubes labeled 1-9.
- Add 100 μL of upper respiratory specimen (e.g. NPS in viral transport media) into each of the nine labeled tubes with lysis buffer
- To prepare samples at a moderate concentration, spike 12 μL of undiluted nCoVPC (rehydrated as described in the CDC 2019-nCoV Real-Time RT-PCR Diagnostic Panel instructions for use) into each tube labeled 1-3 containing lysis buffer and specimen
- To prepare samples at a low concentration, spike 12 μL of 1/10 dilution of nCoVPC into each tube labeled 4-6 containing lysis buffer and specimen
- To prepare negative samples, spike 12 μL of nuclease-free water into each tube labeled 7-9 containing lysis buffer and specimen
- Perform extractions of all nine samples according to the CDC 2019-nCoV Real-Time RT-PCR Diagnostic Panel instructions for use

INSTRUCTIONS FOR PREPARING SAMPLES BEFORE EXTRACTION WITH THE BIOMÉRIEUX NucliSENS easyMAG OR THE BIOMÉRIEUX EMAG

- Refer to the 2019-nCoV Real-Time RT-PCR Diagnostic Panel instructions for use for reconstitution of the materials for use. RNA should be kept cold during preparation and use.
- Make a 1/10 dilution of nCoVPC by adding 5 μL of nCoVPC into 45 μL of nuclease-free water or 10 mM Tris
- Aliquot 1000 µL or 2000 μL of pre-aliquoted easyMAG lysis buffer into each of nine tubes labeled 1-9 for the easyMAG or eMAG, respectively.
- Add 100 μL of upper respiratory specimen (e.g. NPS in viral transport media) into each of the nine labeled tubes with lysis buffer
- To prepare samples at a moderate concentration, spike 12 μL of undiluted nCoVPC (rehydrated as described in the CDC 2019-nCoV Real-Time RT-PCR Diagnostic Panel instructions for use) into each tube labeled 1-3 containing lysis buffer and specimen
- To prepare samples at a low concentration, spike 12 μL of 1/10 dilution of nCoVPC into each tube labeled 4-6 containing lysis buffer and specimen
- To prepare negative samples, spike 12 μL of nuclease-free water into each tube labeled 7-9 containing lysis buffer and specimen
- Perform extractions of all nine samples according to the CDC 2019-nCoV Real-Time RT-PCR Diagnostic Panel instructions for use

PROCEDURE

Follow the CDC 2019-nCoV Real-Time RT-PCR Diagnostic Panel instructions for use for testing the 9 extracted samples at least once.

EXPECTED RESULTS

Moderate nCoVPC samples should be positive for 2019-nCoV.
Low nCoVPC samples should be positive for 2019-nCoV.
Negative upper respiratory samples should be negative for 2019-nCoV.

****** DO NOT DISCARD: Important product-specific information ******

≥ 90% of test results should be in agreement with the expected results. If test results are less than 90% in agreement with expected results, contact CDC at respvirus@cdc.gov.

QUESTIONS

Please send questions or comments by email to respvirus@cdc.gov.

DISTRIBUTION:

Distributed to qualified laboratories by Centers for Disease Control and Prevention, 1600 Clifton Road, Atlanta, GA, 30329 USA
