# Supplementary material for: Modular micro-PCR system for the onsite rapid diagnosis of COVID-19
Source: Microsyst Nanoeng. 2022 Jul 19;8:82. doi: 10.1038/s41378-022-00400-3 (PMC9294789; doi:10.1038/s41378-022-00400-3)
Supplement: Supplementary file 6 — IFU_Resolute 2.0 Rev 1 [file 41378_2022_400_MOESM6_ESM.pdf]

RESOLUTE 2.0

SARS-CoV-2 Direct Detection Kit

Instructions for Use

REF CKFG0002
CKFG0003

Available in Σ 50 Σ 100

IMPORTANT NOTE

The instructions for use must be read carefully prior to use and followed strictly to achieve reliable results. Any deviations from the instructions will have a significant impact on the end result.

IVD

-25°C -15°C

AMT PTE LTD
3 Tuas Lane
Singapore 638612

In collaboration with:

© 2020 AMT Pte Ltd. All rights reserved.

Table of Contents

1. Introduction 3
2. Intended Use 3
3. Summary and Explanation 3
4. Principles of Procedure 3
5. Materials Required (Provided) 4
6. Equipment and Consumables Required (Not Provided) 4
7. Warnings and Precautions 5
8. Reagent Storage, Handling and Stability 6
9. Specimen Collection, Handling and Storage 6
10. Reagent and Controls Preparation 7
11. Assay Setup 7
12. Running a Test 8
13. Data Analysis 9
14. Interpretation of Test Results and Reporting 9
15. Performance Characteristics 10
16. Limitations 15
17. Disposal 16
18. References 16
19. Contact Information and Product Support 16
20. Symbols 16
21. Trademarks and Disclaimers 16

© 2020 AMT Pte Ltd. All rights reserved.

1. Introduction

This instructions for use must be read carefully prior to use and instructions must be followed accordingly. If there are any deviations from this instructions for use (IFU), reliability of this assay results cannot be guaranteed.

RESOLUTE 2.0 is available in 100 reactions (CKFG0002) and 50 reactions (CKFG0003).

2. Intended Use

RESOLUTE 2.0 is a real-time RT-PCR test intended for the qualitative detection of nucleic acid from the SARS-CoV-2 virus in nasopharyngeal swabs specimen from individuals with signs and symptoms of infection who are suspected of COVID-19.

RESOLUTE 2.0 is intended for the direct amplification of Coronavirus SARS-CoV-2 RNA from nasopharyngeal swabs (NPS) in Universal Transport Medium (UTM). RNA extraction is not required for the use of this kit.

This assay has received Provisional Authorisation from the Health Sciences Authority in Singapore.

Results are for the detection of SARS-CoV-2 RNA. The SARS-CoV-2 RNA is generally detectable in respiratory specimens, including nasopharyngeal swabs during the acute phase of infection. A positive result is indicative of the presence of SARS-CoV-2 RNA. Correlation with clinical presentation and other clinical investigations is necessary to determine the patient's infection status. Positive results do not rule out bacterial infection or co-infection with other viruses.

A negative result does not exclude SARS-CoV-2 infection. Clinical correlation is required and if indicated, repeat sampling and use of other clinical and laboratory investigations may be warranted.

The test results from the RESOLUTE 2.0 SARS-CoV-2 detection assay should not be used as the sole basis for patient management decisions.

3. Summary and Explanation

An outbreak of pneumonia caused by a novel coronavirus (SARS-CoV-2) in Wuhan City, Hubei Province, China was initially reported to WHO on December 31, 2019. Coronavirus Disease 2019 (COVID-19) is a new infectious disease and is caused by the SARS-CoV-2 virus. Symptoms include cough, fever, sore throat, shortness of breath and pneumonia. As of 10th April 2020, WHO has reported about 1,500,000 infections with more than 90,000 deaths.

The RESOLUTE 2.0 COVID-19 Real-Time RT-PCR Tests is a molecular *in vitro* diagnostic test that detects the SARS-CoV-2 virus and is based on widely used nucleic acid amplification technology. The product contains oligonucleotide primers and fluorophore-labelled probes, control material used in RT-PCR and the enzyme mix required for the *in vitro* qualitative detection of SARS-CoV-2 RNA in respiratory specimens.

4. Principles of Procedure

RESOLUTE 2.0 is based on real-time reverse transcriptase polymerase chain reaction (RT-PCR) technology, for the qualitative detection of SARS-CoV-2 specific RNA. The RESOLUTE 2.0 SARS-CoV-2 detection assay enables the **direct amplification** of Coronavirus SARS-CoV-2 RNA from nasopharyngeal swabs (NPS) UTM.

In the RESOLUTE 2.0 SARS-CoV-2 detection assay, forward and reverse primers amplify SARS-CoV-2 viral RNA, and are used with fluorescent probes specific to the viral targets. The RESOLUTE 2.0 SARS-CoV-2 panel consists of three (3) assays, an E gene assay which detects members of the Sarbecovirus subgenus of coronavirus, an N gene assay which detects coronavirus type SARS-CoV-2 specifically and an endogenous housekeeping assay targeting the human RNase P gene (RP). The E gene assay amplifies a target from the virus envelope gene and the N gene assay amplifies a target from the virus nucleocapsid gene. The RP assay is included as a control to detect clinical specimen collection failure, cell lysis failure or RT-PCR failure including inhibition of reaction.

© 2020 AMT Pte Ltd. All rights reserved.

5. Materials Required (Provided)

The RESOLUTE 2.0 SARS-CoV-2 Direct Detection Kit contains sufficient reagents for 50 (REF Number CKFG0003) or 100 (REF Number CKFG0002) reactions of each of the 3 assays. Details of the kit components are provided in Table 1A and 1B below.

To prevent repeated freeze-thaw, users are strongly recommended to make aliquots of the Positive Control Template (component 3) and Negative Control (component 4) in separate DNase- and RNase-free tubes before the first use.

Table 1A: Components of RESOLUTE 2.0 – 50 reactions (CKFG0003)

Tube No	Component REF	Component Description	Number of Vials	Volume (µl/vial)
1B	COM023	R2.0 Reaction Mix	1	1158
2B	COM024	R2.0 Enzyme Mix	1	42
3B	COM025	Positive Control Template	1	80
4B	COM026	Negative Control	1	80

Table 1B: Components of RESOLUTE 2.0 –100 reactions (CKFG0002)

Tube No	Component REF	Component Description	Number of Vials	Volume (µl/vial)
1A	COM019	R2.0 Reaction Mix	2	1158
2A	COM020	R2.0 Enzyme Mix	1	84
3A	COM021	Positive Control Template	1	160
4A	COM022	Negative Control	1	160

6. Equipment and Consumables Required (Not Provided)

The equipment and consumables that is required but not provided in this kit is listed below. The names of vendors or manufacturers are provided as examples of suitable product sources. Users have to conduct verification whether using product sources provided or other product sources.

- Desktop centrifuge with a rotor for 1.5mL and 2mL reaction tubes
- Centrifuge that goes up to 1000 x *g* with a rotor for microtiter plates if using 96-well reaction plates, microcentrifuge if using PCR tubes or strips
- Vortex mixer
- Micropipettes (adjustable: 2µl, 10 µl, 100 µl, 200 µl and 1000 µl)
- Disposable pipette tips with filters and aerosol barriers
- 1.5mL microcentrifuge tubes (DNase/RNase free)
- Racks for 1.5mL microcentrifuge tubes
- 2x 96-well -20°C cold blocks for holding the RT-PCR assay set-up PCR tubes
- Disposable PPE, including powder-free gloves and surgical gowns
- Compatible options of RT-PCR platforms are provided in Table 3 below

Table 3: Options of Real-Time PCR Instrument for use with RESOLUTE 2.0

Instrument/Manufacturer	Software	Catalog No.
BioRad CFX96™ TOUCH<br>Real-Time PCR Instrument	BioRad CFX Manager Software Version 3.1	1855195

- Appropriate 96-well reaction plates or reaction tubes with corresponding (optical) closing material. Compatible options (refer to Table 4).

© 2020 AMT Pte Ltd. All rights reserved.

Table 4: PCR Plates/Reaction Tubes Compatible with the recommended qPCR platform

qPCR platform	PCR Plates/Reaction Tubes	Vendor
BioRad CFX96™ TOUCH Real-Time PCR Instrument	- Hard-Shell® PCR Plate, 96-Well, thin-wall (#HSP-9601)<br>- Microseal® 'B' seal (#MSB1001)	BioRad

USERS MUST PERFORM IN-HOUSE VERIFICATION AND VALIDATION IF OTHER PCR SYSTEMS ARE USED.

7. Warnings and Precautions

- Positive results indicate the presence of SARS-CoV-2 RNA.
- Kit has to be kept upright during storage and transportation.
- Prior to use, check the tubes in the kit for leakage or damage. Do not use if the kit box has been tampered.
- Follow standard precautions. All patient specimens should be considered potentially infectious and handled accordingly.
- Do not eat, drink, smoke, apply cosmetics or handle contact lenses in areas where reagents and human specimens are handled.
- Handle all specimens as if infectious using safe laboratory procedures. Refer to your local authority for guidelines, example include the Interim Laboratory Biosafety Guidelines for Handling and Processing Specimens Associated with 2019-nCoV https://www.cdc.gov/coronavirus/2019-nCoV/lab-biosafety-guidelines.html
- Specimen processing should be performed in accordance with national biological safety regulations.
- If infection with 2019-nCoV is suspected based on current clinical and epidemiological screening criteria recommended by public health authorities, specimens should be collected with appropriate infection control precautions.
- Performance characteristics have been determined with nasopharyngeal swabs specimen from human patients with signs and symptoms of respiratory infection.
- The instrument, pipettes and other equipment should be calibrated with defined intervals.
- Use personal protective equipment such as (but not limited to) gloves, eye protection, and lab coats when handling kit reagents while performing this assay and handling materials including samples, reagents, pipettes, and other equipment and reagents.
- Amplification technologies such as PCR are sensitive to accidental introduction of PCR product from previous amplifications reactions. Incorrect results could occur if either the clinical specimen or the real-time reagents used in the amplification step become contaminated by accidental introduction of amplification product (amplicon). Workflow in the laboratory should proceed in a unidirectional manner.
  - Maintain separate areas for assay setup.
  - Always check the expiration date prior to use. Do not use expired reagent. Do not substitute or mix reagent from different kit lots or from other manufacturers.
  - Change aerosol barrier pipette tips between all manual liquid transfers.
  - Maintain separate, dedicated equipment (e.g. pipettes, microcentrifuges) and supplies (e.g. microcentrifuge tubes, pipette tips) for assay setup and handling of clinical specimens.
  - Wear a clean lab coat and powder-free disposable gloves (not previously worn) when setting up assays.
  - Change gloves between samples and whenever contamination is suspected.
  - Keep reagent and reaction tubes capped or covered as much as possible.
  - Primers, probes (including aliquots), and enzyme master mix must be thawed and maintained on cold block at all times during preparation and use.
  - Work surfaces, pipettes, and centrifuges should be cleaned and decontaminated with cleaning products such as 20% bleach, "DNA*Zap*™" or "RNase AWAY®" to minimize risk of nucleic acid contamination. Residual bleach should be removed using 70% ethanol. Turn on UV light to disinfect working surfaces for 30 minutes.
- Dispose of unused kit reagents and human specimens according to local, state, and federal regulations.

© 2020 AMT Pte Ltd. All rights reserved.

8. Reagent Storage, Handling and Stability

- The RESOLUTE 2.0 kit is shipped in a cold chain environment, on dry ice or coolant (between -25°C and -15°C). The components of the kit should arrive cold. If the kit components are not cold upon receipt, or if vials have been compromised during shipment, contact your local distributor for assistance.
- All components are to be stored between -25°C and -15°C upon arrival.
- Protect from light.
- Before initial use, check the product and its components for:
  - Integrity
  - Completeness with respect to number, type and filling
  - Correct labelling
- Use of this product is limited to personnel specially instructed and trained in the techniques of real-time RT-PCR.
- Primer-Probes Mixes, control templates and enzyme mixes must be thawed and kept on a cold block at all times during preparation and use.
- Avoid microbial and nuclease (DNase/RNase) contamination of the specimens and the components of the Kit.
- Always use DNase/RNase-free disposable pipette tips with aerosol barriers.
- Always wear protective disposable powder-free gloves when handling kit components.
- The workflow in the laboratory should proceed in a unidirectional manner. Use separated and segregated working areas for:
  i. sample preparation
  ii. reaction setup and
  iii. amplification/detection activities
- Always wear disposable powder-free gloves in each area and change them before entering a different area.
- Dedicate supplies and equipment to the separate working areas and do not move them from one area to another.
- Store positive and/or potentially positive material separately from all other components of the Kit.
- Do not open the reaction tubes/plates post amplification, to avoid contamination with amplicons.
- Do not autoclave reaction tubes after the RT-PCR, since this will not degrade the amplified nucleic acid and risks contaminating the laboratory area.
- Do not use expired components, refer to the label for expiration date.

9. Specimen Collection, Handling and Storage

Acceptable Specimens

- Nasopharyngeal swabs in universal transport medium (UTM) or equivalent.
- Types of transport medium validated to be compatible with RESOLUTE 2.0 include the Copan Universal Transport Medium™ (UTM®, Copan Diagnostics), ASAN Transport Medium (ASANPHARM) and the BD universal viral transport (UVT) system (Becton Dickinson).
- Compatibility of other types of transport medium must be validated by the users.

Biosafety Precautions on Specimen Handling

- Specimens should always be treated as infectious and/or biohazardous in accordance with safe laboratory procedures.
- Users should handle clinical specimen in biosafety level 2+ and above, and observe the safety requirement of a biosafety level 2+ lab.
- Discard samples and assay waste according to your local safety regulations.

Specimens Preparation

- Samples to be collected into sterile, labelled tubes and shipped at 2-8°C on frozen gel packs.
- Collection of nasopharyngeal or oropharyngeal swabs: flocked swabs are preferred especially for nasopharyngeal swabs. Sterile dacron or Rayon tipped swabs may also be used for oropharyngeal swabs. Specimens in swabs are to be placed immediately in sterile tubes containing 3mL of sterile UTM.
- For delayed testing, specimens can be stored at -70°C and below.

© 2020 AMT Pte Ltd. All rights reserved.

Transporting Specimens

- Specimens must be packaged, shipped and transported according to the current edition of the International Air Transport Association (IATA) Dangerous Goods Regulations. Follow shipping regulations for UN3373 Biological Substance, Category B when sending potential SARS-CoV-2 specimens. For local land transport, follow local biosafety requirements and regulations

10. Reagent and Controls Preparation

Important Notes Before Starting:

- Plate or tube layout is dependent on the throughput of the laboratory.
- To ensure validity of each run, at least one Positive Control (component 3) and one Negative Control (component 4) must be included.
- Once thawed, all reagents should be kept cold either on ice or cool block throughout the preparation and setup.
- It is strongly recommended that users prepare and set up the reaction in segregated areas as follows:

1. **Reagent Preparation Room:**
   a) In a PCR workstation pre-cleaned with 70% ethanol and UV-irradiated, prepare the primary Master Mix.
   b) Transfer the tubes of primary Master Mix via a transfer box to the Sample Preparation Room.

2. **Sample Preparation Room:**
   a) Aliquot the completed Master Mix into the respective wells, followed by Negative Control (component 4), clinical nasopharyngeal swab UTM, and finally, Positive Control Template (component 3), into an appropriate optical 96-well reaction plate or an appropriate optical reaction tube according to a predefined layout.
   b) Seal or cap the loaded optical 96-well reaction plate or optical reaction tube, and centrifuge in a centrifuge with an appropriate rotor for 30 seconds at approximately 1000 *x g* to bring down the reaction mix to the bottom of the optical 96-well reaction plate or optical reaction tube.
   c) Transfer the sealed or capped optical 96-well reaction plate or optical reaction tube via a transfer box to the PCR Room.

3. **PCR Room:**
   a) Load the optical 96-well reaction plate or optical reaction tube in the thermal cycler preloaded with the appropriate cycling profile and start run.

11. Assay Setup

RT-PCR Reaction Master Mix Preparation

- Do not combine components of assay kits with different lot numbers.
- To prepare the master mix, thaw R2.0 reaction mix (component 1) and negative control (NC, component 4) at room temperature; thaw R2.0 enzyme mix (component 2) and positive control template (PC, component 3) on ice.
- Add the components, by pipetting, sequentially according to Table 4 below. Mix the master mix by gentle pipetting or pulse-vortexing, and centrifuge briefly before use.
- Depending on the intended number of reactions (indicated as "*R*"), the following amount is recommended:
  - o For number of reaction 20 and below, $N = R + 1$.
  - o For number of reactions above 20 and below 40, $N = R + 2$.
  - o For number of reactions above 40 and below 60, $N = R + 3$.
  - o For number of reactions above 60 and below 80, $N = R + 4$.
  - o For number of reactions above 80 to 96, $N = R + 5$.

© 2020 AMT Pte Ltd. All rights reserved.

Table 4: Master Mix Preparation

Sequence	Component Label	Master Mix	Volume per Reaction (µl)	Location
A	1 (COM019 or COM023)	R2.0 Reaction Mix	*N* x 19.30	Reagent Preparation Room
B	2 (COM020 or COM024)	R2.0 Enzyme Mix	*N* x 0.70	
		**Total**	***N* x 20.00**	

RT-PCR Setup

1) Pipette **20 µL of the Master Mix** into each required well of an appropriate optical 96-well reaction plate or an appropriate optical reaction tube.

Test Specimen or Control Template Addition

1) Prior to moving to the sample preparation room, prepare the Negative Control (NC) reaction by pipetting **5 µL of Negative Control (NC**, component 4) into the NC sample well. Ensure at least 1 NC is included per RT-PCR run.
2) Cover the entire reaction plate and move the reaction plate to the specimen handling area.
3) Gently mix the UTM samples by pulse-vortexing or gentle pipetting up and down with a pipette set at 50-80% of the volume of the UTM in the sample tube.
4) Add **5 µL of the UTM** of the first sample into all the wells labelled for that sample.
5) Change tips after each sample addition.
6) When all test UTM specimen have been added, add **5µL of Positive Control Template (PC**, component 3) to the PC sample well. Ensure at least 1 PC is included per RT-PCR run.
7) Thoroughly mix the test UTM samples or PC with the Master Mix by gently pipetting up and down.
8) Close the 96-well reaction plate with appropriate lids or optical adhesive film and the reaction tubes with appropriate lids.
9) Centrifuge the 96-well reaction plate in a centrifuge with a microtiter plate rotor for 30 seconds at approximately 1000 x *g*. If PCR tubes or strips are used instead of 96-well reaction plate, briefly centrifuge the reaction tubes for 10-15 seconds.

12. Running a Test

Setting-up of a real-time RT-PCR run on a BioRad CFX96™ TOUCH Real-Time PCR Instrument:

- To create a run template
  - Launch the Bio-Rad CFX96™ Touch Real-time PCR Instrument by double clicking on the Bio-Rad CFX96™ Touch System icon on the desktop.
  - A new window, Startup Wizard, should appear.
  - The **Startup Wizard** screen will appear. Select the following:
    - Instrument: **CFX96**
    - Run type: **User-defined**
    - After clicking User-defined, the **Run Setup** screen will appear.
    - After clicking Create New, the **Protocol Editor** screen will appear to create a new protocol.
- Set up the thermal cycling conditions as detailed in Table 5 below.

Table 5. RESOLUTE 2.0 Thermal Cycling Profile

Step	Temperature (°C)	Duration (Hr:Min:Sec)	No. of Cycles	Detection
Reverse Transcription	55	00:15:00	1	-
RT inactivation / Initial Denaturation	95	00:04:00	1	-
Denaturation	95	00:00:03	45	-
Annealing (Data Collection)	62	00:00:30		✔ All Channels

© 2020 AMT Pte Ltd. All rights reserved.

13. Data Analysis

Recommendation for data analysis on the BioRad CFX Manager Software Version 3.1:

- **Single Manual Threshold Ct**, at **default Manual Baseline**:
  - Under **Settings,**
    - go to '**Cq Determination Mode**'
    - choose '**Single Threshold**'
  - Under **Settings,**
    - go to '**Baseline Setting**'
    - choose '**Baseline Subtracted Curve Fit**' and
    - choose '**Apply Fluorescent Drift Correction**'
- Set threshold line **within the exponential phase** of the typical sigmoidal fluorescence growth curves, above any background signal.
  - Manual threshold setting of 100 based on the RFU saturation around 5000-7000 is recommended.

14. Interpretation of Test Results and Reporting

Interpretation of RESOLUTE 2.0 test results MUST take into consideration the C_T values, as well as the shape of the amplification curve.

Recommendation on the interpretation of test run validity and test result interpretation are as summarized in Table 6 and Table 7.

- qPCR result is considered **Positive** if
  - C_T values ≤ 40, and
  - there is a presence of sigmoidal growth curve

- qPCR result is considered **Negative** if
    - C_T values > 40 or undetermined, and
    - there is an absence of sigmoidal growth curve

Table 6: Expected Performance of Controls

Sample	SARS-CoV-2 E gene Assay (FAM)	SARS-CoV-2 N Gene Assay (HEX)	Control RNase P Assay (TEX)	Interpretation
Positive Control (PC)	**Positive**[a]	**Positive**[a]	**Positive**	QC passed
Negative Control (NC)	**Negative**	**Negative**	**Negative**	

a. Note that CT values for E gene and N Gene assays in PC control runs are likely within 28-31, based on the recommended threshold setting at 100 on BioRad CFX as described above. Users may perform independent calibration runs on their own instrument to establish a passing criteria. Marked deviation from the recommended criteria should be queried before releasing results.

© 2020 AMT Pte Ltd. All rights reserved.

Table 7: Interpretation of Clinical Test Sample Results

SARS-CoV-2 E gene Assay (FAM)	SARS-CoV-2 N Gene Assay (HEX)	Control RNase P Assay (TEX)	Interpretation
Positive	Positive	Positive or Negative	SARS-CoV-2 is **Detected**
Negative	Positive	Positive or Negative	
Positive	Negative	Positive or Negative	**Equivocal**[b] Sample should be re-tested with original UTM sample or new samples. For samples with a repeated equivocal result, the result should be reported as "*Equivocal*", and an alternative confirmatory assay is recommended.
Negative	Negative	Positive	SARS-CoV-2 is **Not Detected**
Negative	Negative	Negative	Test Run is **Invalid**

b. Note that as the E gene assay in RESOLUTE 2.0 detects Pan-Sarbecovirus 1, a positive result for E gene assay and a negative result for N gene assay could indicate a) low amount of virus RNA, or b) mutation in the N gene, or c) infection with some other Sarbecovirus (e.g. SARS-CoV or other animal Sarbechovirus not known to infect humans). Additional confirmatory test is recommended for samples with repeated equivocal test results.

If a false positive occurs with one or more NC reactions, reagent/NC contamination may have occurred. Repeat PC and NC test run with new tube of NC reagent.

- Clinical correlation with patient history and other diagnostic information is necessary to determine patient infection status. For results with high Ct values (37-40) for both genes, the clinician should obtain a second sample for testing if there is doubt about the diagnosis.

15. Performance Characteristics

Limit of Detection (LoD)

LoD studies determines the lowest detectable concentration (in RNA copies/µL) of the SARS-CoV-2 pseudo virus (Armoured RNA Quant SARS CoV2 Panel, Asuragen, Cat no. #52036) at which approximately 95% of all (true positive) replicates test positive.

Known concentration of serially diluted SARS-CoV-2 pseudo virus was spiked into clinically negative nasopharyngeal swab UTM, which have been verified to be SARS-CoV-2 negative by the cobas® SARS-CoV-2 assay (Roche, P/N 09175431190). Analytical LoD was determined by first establishing a preliminary LoD, then by repeating the LoD tests using 20 replicates of two-fold serial dilution of the pseudo virus spiked into the clinical specimen matrix mimic.

RESOLUTE 2.0 test was performed using 5µl of UTM per standard protocol in Table 4 and detected on a BioRad CFX 96 TOUCH Real-Time PCR Instrument.

Table 8: Limit of detection confirmation of the RESOLUTE 2.0 **(BioRad CFX96™ TOUCH PCR Instrument)**

Target	SARS-CoV-2 E Gene Assay (FAM)		SARS-CoV-2 N Gene Assay (HEX)	
RNA copies/µL	15.0	10.0	15.0	10.0
No. of Positive / Total No. of Replicates (%)	20/20 (100%)	20/20 (100%)	20/20 (100%)	19/20 (95%)
Mean C_T	34.0	35.0	35.0	35.0
Standard Deviation (C_T)	0.4	0.5	0.6	0.8

The analytical LoD for the RESOLUTE 2.0 was determined to be 10 RNA copies/µL for the RESOLUTE 2.0 E Gene and N Gene assays for the BioRad CFX96™ TOUCH Real-time PCR system.

© 2020 AMT Pte Ltd. All rights reserved.

Limit of Blank (LoB)

LoB studies determine the highest apparent analyte concentration expected to be found when replicates of a blank sample containing no analyte are tested.

To determine the LoB of RESOLUTE 2.0 SARS-CoV-2 detection assay, a 'blank sample' was created using UTM.

19 replicates of this 'blank sample' were tested using the RESOLUTE 2.0 assay per standard protocol in Table 4. Detection was performed using a BioRad CFX 96 TOUCH Real-time PCR instrument. Table 9 shows the result of the LoB study.

Table 9: Limit of Blank confirmation of the RESOLUTE 2.0

Sample	Work Conc (copies/uL)	Log Dilution	E-Gene (FAM)	N-Gene (HEX)	RNase P (TxR)		
			C_T	C_T	C_T	Ave	SD
'Blank Sample' UTM	N/A	N/A	N/A	N/A	N/A	N/A	N/A
			N/A	N/A	N/A		
			N/A	N/A	N/A		
			N/A	N/A	N/A		
			N/A	N/A	N/A		
			N/A	N/A	N/A		
			N/A	N/A	N/A		
			N/A	N/A	N/A		
			N/A	N/A	N/A		
			N/A	N/A	N/A		
			N/A	N/A	N/A		
			N/A	N/A	N/A		
			N/A	N/A	N/A		
			N/A	N/A	N/A		
			N/A	N/A	N/A		
			N/A	N/A	N/A		
			N/A	N/A	N/A		
			N/A	N/A	N/A		
			N/A	N/A	N/A		
PC	100	N/A	30.9	30.7	31.1	31	N/A
NC	N/A	N/A	N/A	N/A	N/A	N/A	N/A

Analytical Specificity

In silico analysis

Primers in the RESOLUTE 2.0 E gene and N gene assays were individually mapped to the NCBI sequences of the organisms listed in Table 10. Potential cross reactivity due to PCR amplification was noted if any of the primer pairs mapped to a sequence, with more than 50% homology, on opposite strands less than 1000 nucleotide apart.

No potential unintended cross reactivity is expected based on this *in silico* analysis.

Table 10. *In silico* analysis for RESOLUTE 2.0 SARS-CoV-2 assays

Strain	RefSeq ID	SARS-CoV-2 E Gene Pan-Sarbecovirus Assay (FAM)	SARS-CoV-2 N Gene Assay (HEX)
CoV 229E	NC_028752.1	No alignment was found	No alignment was found
CoV OC43	NC_006213.1	No alignment was found	No alignment was found
CoV HKU1	NC_006577.2	No alignment was found	No alignment was found
CoV NL63	NC_005831.2	No alignment was found	No alignment was found
SARS-CoV	NC_004718.3	*F:100%; R:100%; Distance< 100bp*	No alignment was found
MERS	NC_038294.1	No alignment was found	No alignment was found
AdV	NC_001662.1	No alignment was found	No alignment was found

© 2020 AMT Pte Ltd. All rights reserved.

Strain	RefSeq ID	SARS-CoV-2 E Gene Pan-Sarbecovirus Assay (FAM)	SARS-CoV-2 N Gene Assay (HEX)
HMPV	NC_039199.1	No alignment was found	No alignment was found
HPIV1	NC_003461.1	No alignment was found	No alignment was found
HPIV2	NC_003443.1	No alignment was found	No alignment was found
HPIV3	NC_038270.1	No alignment was found	No alignment was found
HPIV4	NC_021928.1	No alignment was found	No alignment was found
Flu A	NC_026431.1	No alignment was found	No alignment was found
Flu B	NC_002205.1	No alignment was found	No alignment was found
EV	NC_038308.1	No alignment was found	No alignment was found
RSV	NC_038235.1	No alignment was found	No alignment was found
RV	NC_038311.1	No alignment was found	No alignment was found
Chlamydia pneumoniae	NC_005043.1	No alignment was found	No alignment was found
Haemophilus influenzae	NZ_LN831035.1	No alignment was found	No alignment was found
Legionella pneumophila	NZ_LR134380.1	No alignment was found	No alignment was found
MTB Mycobacterium bovis subsp. Bovis	CP040832.1	No alignment was found	No alignment was found
Streptococcus pneumoniae	NZ_LN831051.1	No alignment was found	No alignment was found
Streptococcus pyrogenes	NZ_LN831034.1	No alignment was found	No alignment was found
Bordetella pertussis	NC_018518.1	No alignment was found	No alignment was found
Mycoplasma pneumoniae	NZ_CP010546.1	No alignment was found	No alignment was found
Influenza C	NC_006306.2	No alignment was found	No alignment was found
Parechovirus	NC_038319.1	No alignment was found	No alignment was found
Candida albicans	CP032012.1 - CP032019.1	No alignment was found	No alignment was found
Corynebacterium diphtheriae	NZ_LN831026.1	No alignment was found	No alignment was found
Legionella non-pneumophila	KU979014.1	No alignment was found	No alignment was found
Bacillus anthracosis (Anthrax)	CP014179.1	No alignment was found	No alignment was found
Moraxella cararrhalis	NC_014147.1	No alignment was found	No alignment was found
Neisseria elongate and meningitides	NZ_LR134525.1	No alignment was found	No alignment was found
Pseudomonas aeruginosa	NC_002516.2	No alignment was found	No alignment was found
Staphylococcus epidermis	NZ_CP035288.1	No alignment was found	No alignment was found
Chlamydia psittaci	NC_017287.1	No alignment was found	No alignment was found
Coxiella burneti (Q-Fever)	NC_002971.4	No alignment was found	No alignment was found
Streptococcus aureus	NC_007795.1	No alignment was found	No alignment was found

Wet-lab Specificity Testing

In addition to the *in silico* analysis, cross reactivity of the RESOLUTE 2.0 E gene and N gene assays were evaluated by testing

1. Samples from QCMD 2018 Coronavirus panel and QCMD 2019 MERS Coronavirus Panel (Qnostics Ltd), and
2. Inactivated organisms in the NATrol™ Respiratory Panel -1 (pool 3) and -2 (Zeptometrix, Catalog number: Catalog number: NATRPP1 and NATRPC2-BIO)

RESOLUTE 2.0 test was performed using 5µl of UTM per standard protocol in Table 4 and detected on a BioRad CFX 96 Real-Time PCR Instrument.

Results obtained showed that the RESOLUTE 2.0 E Gene and N Gene assays are specific and do not detect the list of pathogens tested. Details of the wet-lab specificity testing results are summarized in Table 11 – 13.

© 2020 AMT Pte Ltd. All rights reserved.

Table 11. Cross reactivity test results on Zeptometrix NATrol™ Respiratory Panel 2

Virus/Organism	Strain	Virus/organisms in the RP2 Controls		RESOLUTE 2.0 SARS-CoV-2 Detection Kit	
		RP2 Control 1	RP2 Control 2	E Gene Assay	N Gene Assay
Adenovirus Type 1	N/A	Positive	Negative	Negative	Negative
Adenovirus Type 3	N/A	Positive	Negative		
Adenovirus Type 31	N/A	Positive	Negative		
C. pneumoniae	CWL-029	Positive	Negative		
Influenza A 2009 H1N1pdm	A/NY/02/2009**	Positive	Negative		
Influenza A H3N2	A/Brisbane/10/07	Positive	Negative		
Human Metapneumovirus Type 8	Peru6-2003	Positive	Negative		
M. pneumoniae	M129	Positive	Negative		
Parainfluenza Type 1	N/A	Positive	Negative		
Parainfluenza Type 4	N/A	Positive	Negative		
Rhinovirus Type 1A	N/A	Positive	Negative		
B. parapertussis	A747	Negative	Positive		
B. pertussis	A639	Negative	Positive		
Coronavirus 229E	N/A	Negative	Positive		
Coronavirus HKU-1	Recombinant	Negative	Positive		
Coronavirus NL63	N/A	Negative	Positive		
Coronavirus OC43	N/A	Negative	Positive		
Influenza A H1N1	A/New Cal/20/99	Negative	Positive		
Influenza B	B/Florida/02/06	Negative	Positive		
Parainfluenza Type 2	N/A	Negative	Positive		
Parainfluenza Type 3	N/A	Negative	Positive		
RSV Type A	2006 Isolate	Negative	Positive		

**Please note that although similar in nomenclature, this is a 2009 H1N1 pandemic Influenza strain and does NOT correlate with the seasonal 2009 Influenza strains found in the Fludb.org database. For reference, the NCBI Taxon IDs for the seasonal Influenza strains listed in the Fludb.org database are: A/New York/01/2009 (H1N1) - 666252; B/New York/01/2009 - 664512; A/New York/02/2009 (H1N1) - 666298; and A/New York/03/2009 (H3N2) – 659637.

Table 12. Cross reactivity test results on Zeptometrix NATrol™ Respiratory Panel -1 (pool3)

Virus/Organism	Strain	RESOLUTE 2.0 SARS-CoV-2 Detection Kit	
		E Gene Assay	N Gene Assay
Influenza A H3	A/Brisbane/10/17	Negative	Negative
Respiratory Syncytial Virus B	CHH93 (18)-18		
Coronavirus OC43	N/A		
Coronavirus HKU-1	N/A		

Table 13. Cross reactivity test results on QCMD samples (MERS/Coronavirus)

Virus/Organism	Strain	RESOLUTE 2.0 SARS-CoV-2 Detection Kit	
		E Gene Assay	N Gene Assay
QCMD 2018 Coronavirus panel			
Coronavirus	NL63	Negative	Negative
Coronavirus	229E		
Coronavirus	HKU		
Coronavirus	OC43		
QCMD 2019 MERS Coronavirus panel			
MERS Coronavirus	N/A	Negative	Negative

© 2020 AMT Pte Ltd. All rights reserved.

Clinical Performance

The assays were tested with 66[1] nasopharyngeal swab specimens (31 SARS-CoV-2 positives, 2 presumptive positive, 34 SARS-CoV-2 negatives) in universal transport medium (UTM). These were specimens previously tested for presence or absence of SARS-CoV-2 by the cobas® SARS-CoV-2 assays (Roche, P/N 09175431190). Samples were randomized and blinded in the clinical performance evaluation.

RESOLUTE 2.0 test was performed using 5µl of UTM per standard protocol in Table 4 and detected on a BioRad CFX 96 Real-Time PCR Instrument.

All clinical specimens were tested positive for human nucleic acids using the RP assay.

Results of the clinical performance evaluation between RESOLUTE 2.0 SARS-CoV-2 detection Kit and the cobas® SARS-CoV-2 assays are summarized in Table 14.

TABLE 14. Clinical performance of RESOLUTE 2.0 against cobas® SARS-CoV-2 assay

		cobas SARS-CoV-2		
		**Positive**	**Presumptive Positive**	**Negative**
**RESOLUTE 2.0**	**Detected**	31	2	1[2]
	**Equivocal**	0	0	1
	**Not Detected**	0	0	32
	**Total**	31	2	34

Interfering Substances

Interference study was conducted to demonstrate that potentially interfering substances that may be found in the upper respiratory tract do not interfere with the detection of SARS-CoV-2 RNA in the RESOLUTE 2.0 assays.

Ten (10) potential interfering substances in the concentration listed below were tested in Table 15. The analytical performance of the RESOLUTE 2.0 assays was assessed in the presence and absence of each of these substances.

None of the substances listed in Table 15 tested in the interference study demonstrated interference.

Table 15. List of substances tested in interference study

Substances	Active Ingredient	Description	Concentration Tested
Afrin Nasal Spray[c]	Oxymetazoline	A selective α_1 adrenergic receptor agonist and α_2 adrenergic receptor partial agonist	5%
Avamys Nasal Spray	Fluticasone	Steroids used to treat nasal symptoms	5%
Tamiflu	Oseltamivir	An antiviral medication used to treat and prevent influenza A and influenza B	2.2 µg/mL
iliadin Nasal Spray	Saline	A mixture of sodium chloride in water and has a number of uses in medicine	14%

[1] *While cobas test was performed on fresh UTM samples, the samples underwent heat inactivation and 1-2 cycles of freeze-thaw before the RESOLUTE 2.0 clinical evaluation study. To rule out the possibility of viral RNA degradation due to heat inactivation and/or freeze-thaw effect, an independent SARS-CoV-2 detection assay, Fortitude 2.1, was used to verify RNA quality before RESOLUTE 2.0 clinical evaluation. The evaluation of RESOLUTE 2.0 clinical performance against cobas was performed after excluding cobas-positive samples that were Equivocal or Negative for Fortitude 2.1 as it indicated likelihood of sample degradation.*

[2] *This sample showed late CT on the N gene assay on RESOLUTE 2.0. When the extracted RNA from the residual UTM sample was tested on the Fortitude™ Kit 2.1, SARS-CoV-2 RNA was detected with one of the two targets in the Fortitude™ Kit 2.1 test showing late CT value.*

© 2020 AMT Pte Ltd. All rights reserved.

Substances	Active Ingredient	Description	Concentration Tested
Tobrex	Tobramycin	An aminoglycoside antibiotic derived from Streptomyces tenebrarius that is used to treat various types of bacterial infections, particularly Gram-negative infections	10% (0.3mg/mL)
Mucin	Mucin	An endogenous interfering substance	60 µg/mL
Rhinocort Allergy Spray	Budesonide	A steroid used to treat nasal symptoms such as congestion, sneezing, and runny nose caused by seasonal or year-round allergies	5%
Nasacort Allergy Nasal Spray	Triamcinolone acetonide	A nasal steroid used to treat sneezing, itching, and runny nose caused by seasonal allergies or hay fever	5%
Neo-Syephrine Nasal Spray	Phenylephrine hydrochloride	A decongestant used to treat nasal congestion and sinus pressure caused by allergies, the common cold, or the flu	5%
Zicam Cold Remedy Nasal Spray	Zinc acetate, Zinc gluconate	A homeopathic nasal spray used to help relieve cold symptoms and nasal congestion	5%

c. with 5% Afrin Nasal Spray, while there was no effect on test performance based on C_T values, we noted a reduction in the E gene RFU. The interference study reporting is limited to the percentage Afrin tested in this report.

16. Limitations

- As a comprehensive interference study was not performed for the RESOLUTE 2.0 SARS-CoV-2 Direct Detection Kit. Samples with abnormally high background signal should be repeated with RNA extraction, followed by conventional RT-PCR test.
- All users, analysts, and any person reporting diagnostic results should be trained to perform this procedure by a competent instructor. They should demonstrate their ability to perform the test and interpret the results prior to performing the assay independently.
- Performance of the RESOLUTE 2.0 SARS-CoV-2 Detection Assay has only been established in nasopharyngeal swabs specimens.
- Negative results do not preclude COVID-19 infection and should not be used as the sole basis for treatment or other patient management decisions. Optimum specimen types and timing for peak viral levels during infections caused by SARS-CoV-2 have not been determined. Collection of multiple specimens (types and time points) from the same patient may be necessary to detect the virus.
- A false negative result may occur if a specimen is improperly collected, transported or handled. False negative results may also occur if amplification inhibitors are present in the specimen or if inadequate numbers of organisms are present in the specimen.
- Do not use any reagent past the expiration date.
- Test performance can be affected because the epidemiology and clinical spectrum of infection caused by SARS-CoV-2 is not fully known. For example, clinicians and laboratories may not know the optimum types of specimens to collect, and, during the course of infection, when these specimens are most likely to contain levels of viral RNA that can be readily detected.
- Detection of viral RNA may not indicate the presence of infectious virus or that SARS-CoV-2 is the causative agent for clinical symptoms.
- The performance of this test has not been established for monitoring treatment of SARS-CoV-2 infection.
- The performance of this test has not been established for screening of blood or blood products for the presence of SARS-CoV-2.
- This test cannot rule out diseases caused by other bacterial or viral pathogens.
- RESOLUTE 2.0 COVID-19 Real-Time RT-PCR Test results shall not be used as the sole means for clinical diagnosis and treatment.
- RNA viruses in particular show substantial genetic variability. Although continuous efforts were made to monitor potential mutation in the target regions that might result in mis-match between the primers probes and the target

© 2020 AMT Pte Ltd. All rights reserved.

sequences based on available viral sequences, new mutation might result in diminished assay performance and possible false negative results.

17. Disposal

Dispose of hazardous or biologically contaminated materials in compliance to national regulations.

18. References

1. Centers for Disease Control and Prevention. Biosafety in Microbiological and Biomedical laboratories (refer to latest edition). http://www.cdc.gov/biosafety/publications/
2. Centers for Disease Control and Prevention. https://www.cdc.gov/coronavirus/2019-ncov/index.html.
3. US Department of Labor, Occupational Safety and Health Administration. 29 CFR Part 1910.1030. Bloodborne Pathogens.
4. Laboratory testing for 2019 novel coronavirus (2019-nCoV) in suspected human cases: World Health Organization; 2020.
5. Centers for Disease Control and Prevention (CDC). Interim Guidelines for Collecting, Handling, and Testing Clinical Specimens from Persons Under Investigation (PUIs) for Coronavirus

19. Contact Information and Product Support

For information and product support, contact prdt-resolute@amt-mat.com

20. Symbols

REF — Catalogue number

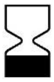
Expiry Date

LOT — Batch number

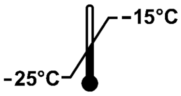

Storage temperature range

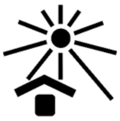
Keep away from sunlight

IVD — Product is an *In vitro* diagnostic medical device

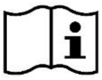
Consult instructions for use

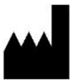
Manufacturer

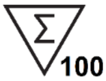

Contains sufficient for 100 tests

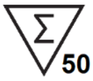

Contains sufficient for 50 tests

21. Trademarks and Disclaimers

RESOLUTE 2.0 should only be used for the intended purpose and in accordance with the Instructions for Use. AMT PTE LTD is not liable for any damage or loss that may result from the use of the test.

All other trademarks that appear in this IFU are the property of their respective owners.

© 2020 AMT Pte Ltd. All rights reserved.
